# Ligand effects, solvent cooperation, and large kinetic solvent deuterium isotope effects in gold(I)-catalyzed intramolecular alkene hydroamination

**DOI:** 10.3762/bjoc.20.43

**Published:** 2024-02-29

**Authors:** Ruichen Lan, Brock Yager, Yoonsun Jee, Cynthia S Day, Amanda C Jones

**Affiliations:** 1 Chemistry, Wake Forest University, 1834 Gulley Rd., Winston-Salem, NC, 27109, USA

**Keywords:** alkene hydroamination, general acid catalysis, gold catalysis, isotope effect, phosphine ligand effect, solvent effect

## Abstract

Kinetic studies on the intramolecular hydroamination of protected variants of 2,2-diphenylpent-4-en-1-amine were carried out under a variety of conditions with cationic gold catalysts supported by phosphine ligands. The impact of ligand on gold, protecting group on nitrogen, and solvent and additive on reaction rates was determined. The most effective reactions utilized more Lewis basic ureas, and more electron-withdrawing phosphines. A DCM/alcohol cooperative effect was quantified, and a continuum of isotope effects was measured with low KIE’s in the absence of deuterated alcoholic solvent, increasing to large solvent KIE’s when comparing reactions in pure MeOH to those in pure MeOH-*d*_4_. The effects are interpreted both within the context of a classic gold π-activation/protodeauration mechanism and a general acid-catalyzed mechanism without intermediate gold alkyls.

## Introduction

Since the seminal 1998 report by Teles et al. on the gold(I)-catalyzed addition of alcohols to alkynes [1], a multitude of gold-catalyzed reactions have been reported. Great successes in mechanistic analysis and synthetic methods have been achieved for allene and alkyne activation, while the activation of alkenes remains challenging. Advances in asymmetric catalysis [2–10], C–N [11–17] and C–C functionalization [18–19] reveal opportunities, but harsh conditions and limited substrate scope present problems. Intramolecular reactions almost invariably require geminal substitution or backbone heteroatoms, internal alkenes are often not tolerated, and intermolecular reactions require high temperatures which can lead to significant catalyst decomposition [20]. This is usually addressed by employing bulky or strong donor ligands [21–22]. Novel strategies tackle catalyst stability by changing the chloride scavenger [23] or adding other coordinating moieties [24–25].

**Scheme 1:**
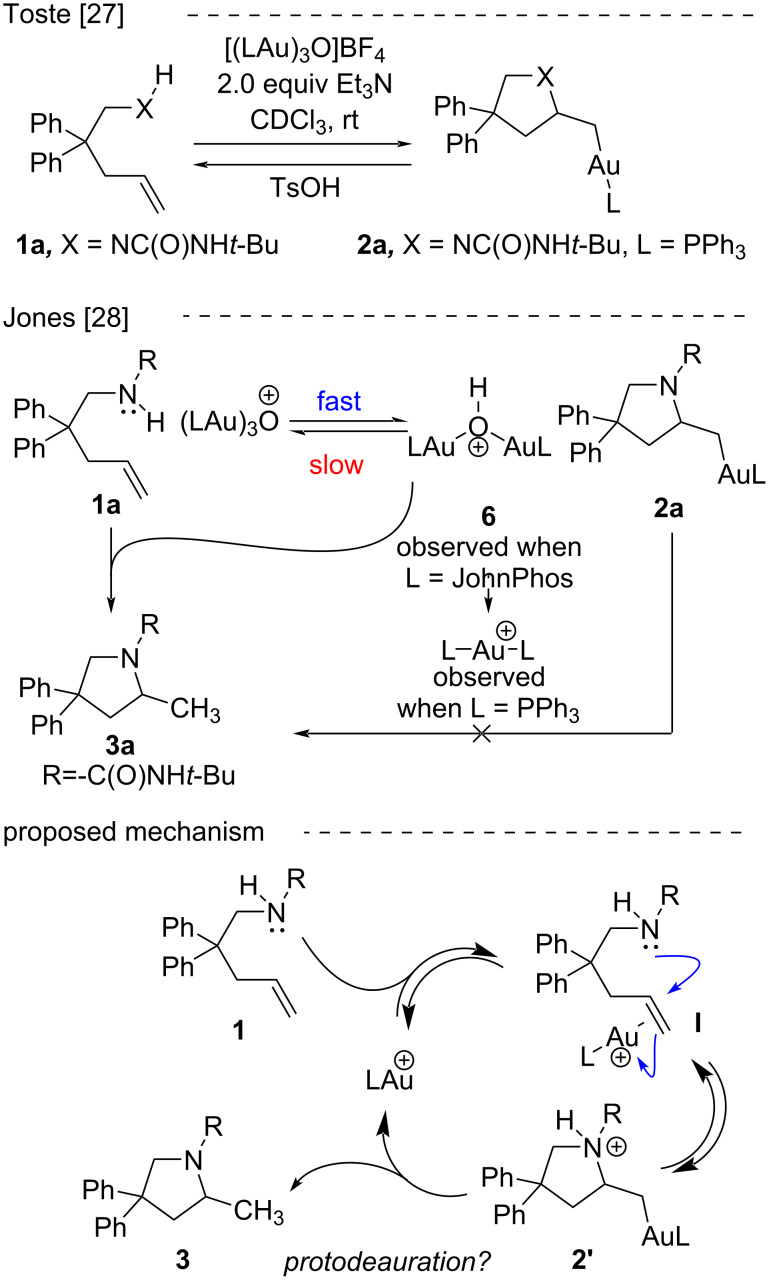
Proposed mechanism and observation of alkylgold intermediates.

Hartwig et al. have argued that a Brønsted acid generated in situ from metal triflates may be the “real” catalyst promoting some alkene functionalizations [26]. Therefore, the possibility of competing Brønsted acid catalysis in gold-catalyzed alkene functionalization remains a consideration [2], and while it is assumed that alkene activations follow the same prototypical mechanisms as allene and alkyne activations, that is (1) π-activation with nucleophilic attack followed by (2) protodeauration (Scheme 1), the depth of experimental mechanistic validation achieved for allenes and alkynes have not been reproduced with alkenes. In an important foundational study by Toste, the expected alkylgold intermediate from intramolecular alkene hydroamination was isolated, however, turnover protodeauration was not confirmed [27]. Follow-up studies in our lab revealed that the alkylgold intermediate (**2a**, Scheme 1) reacts significantly slower than observed rates for catalytic hydroamination, suggesting it is not a viable intermediate in the catalytic cycle [28]. It has been shown that C(sp^2^)-vinylgold intermediates (expected from allene/alkyne addition) are more reactive than the C(sp^3^)-alkylgold intermediates expected from alkene addition [29]. Another study demonstrated the inefficiency of protodeauration in the presence of (albeit more basic) alkylamines [30]. These studies cast doubt on protodeauration as the final step of alkene hydroamination, however, an alternative mechanistic model remains elusive.

There are significant similarities between gold- and acid-catalyzed alkene additions, further confounding easy conclusions about the operative mechanism. Early gold-catalyzed alkene hydroaminations were shown to proceed with *anti-*selectivity and that was used as support for gold catalysis [15], but mechanism studies of triflic acid catalysis showed a preference for *anti-*selectivity as well [31]. Despite similarities, control studies indicate meaningful differences in catalytic activity between gold and HOTf, however, they are not easily explained by simple either/or mechanisms [14,32–33].

Due in part to optimization challenges and in part to remaining gaps in characterizing structure–activity relationships for alkene hydroamination, we sought to obtain additional understanding by undertaking a ^1^H NMR spectroscopic kinetic survey of solvent, ligand, and substituent effects on the general reaction **1** → **3** (with a variety of *N*-protecting groups), to supplement known qualitative observations. We found that, (1) electron-withdrawing phosphines accelerate hydroamination, (2) reactions are faster with more Lewis basic urea substrates, (3) mixed solvents are uniquely able to enhance rates, with protic methanol and DCM identified as the best combination, and (4) kinetic isotope effects are variable depending on the concentration of protons in solution with small deuterium KIE’s at low concentration of deuterated species and large solvent KIE’s when performed in pure CH_3_OH versus CD_3_OD. Connections between catalyst activity and decomposition were made and a structurally interesting new bisphosphine–gold complex was isolated. Although our results do not provide conclusive evidence for or against turnover protodeauration, they indicate strong parallels to general acid catalysis. There is little doubt that gold is required for the transformation, but the combination of solvent and substrate effects suggest that instead of acting as a specific alkene activator, it may instead create generally acidic conditions that initiate cyclization [34–35]. These observations are critical for informing future discussion and experiments related to this important reaction.

## Results

### Ligand effect

To examine the catalytic activity of gold with ligands of different electronic properties, we used our recently developed series of bisbiphenylphosphine ligands, RP(*o*-biphenyl)_2_ (R = OPh, Ph, *t*-Bu) [36–37]. When urea alkene **1a** (0.1 M, CD_2_Cl_2_) was treated with 1 mol % LAuOTf, where L = PhOP(*o*-biphenyl)_2_ (**4a**), PhP(*o*-biphenyl)_2_ (**4b**), *t*-BuP(*o*-biphenyl)_2_ (**4c**), the fastest rate to form **3a** was observed with the ligand with the greatest π-acceptor character and weakest donor character, namely L = PhOP(*o*-biphenyl)_2_ (Table 1) [38]. Notable amounts of scatter and a narrow range of observed rates, however, reveal the differences to be relatively minor (Table 1, entry 1, *k*_rel(_**_4a_**_/_**_4c_**_)_ = 3.6). Nevertheless, each observed rate was outside of one standard deviation from the average of 3 trials, confirming the overall reactivity trend. In hindsight, the ligand effect here may be expected to be small since the bisbiphenyl scaffold contains 2 of 3 identical substituents. Nevertheless, these comparisons confirm the correlation of faster rate with more electron-withdrawing ligand. Experiments with other substrates display consistent results (further discussion below, Table 6, catalyst **4a** reacts faster than **4c** in the reaction of carbamate **1b** → **3b**).

**Table 1:** Relative rates of **1a** hydroamination with gold phosphine triflate catalysts (average of 3 trials each).

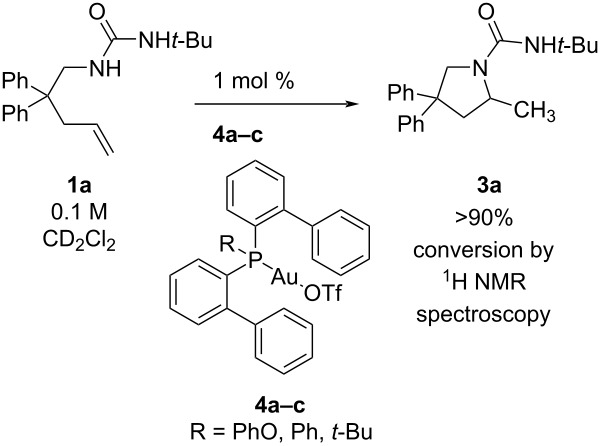			

Entry	R	10^5^∙*k*_obs_/s^−1^	*k*_rel_

1	PhO (**4a**)	206 ± 37	3.6
2	Ph (**4b**)	106 ± 13	1.8
3	*t*-Bu (**4c**)	58 ± 27	1

### Solvent effect

**Figure 1:**
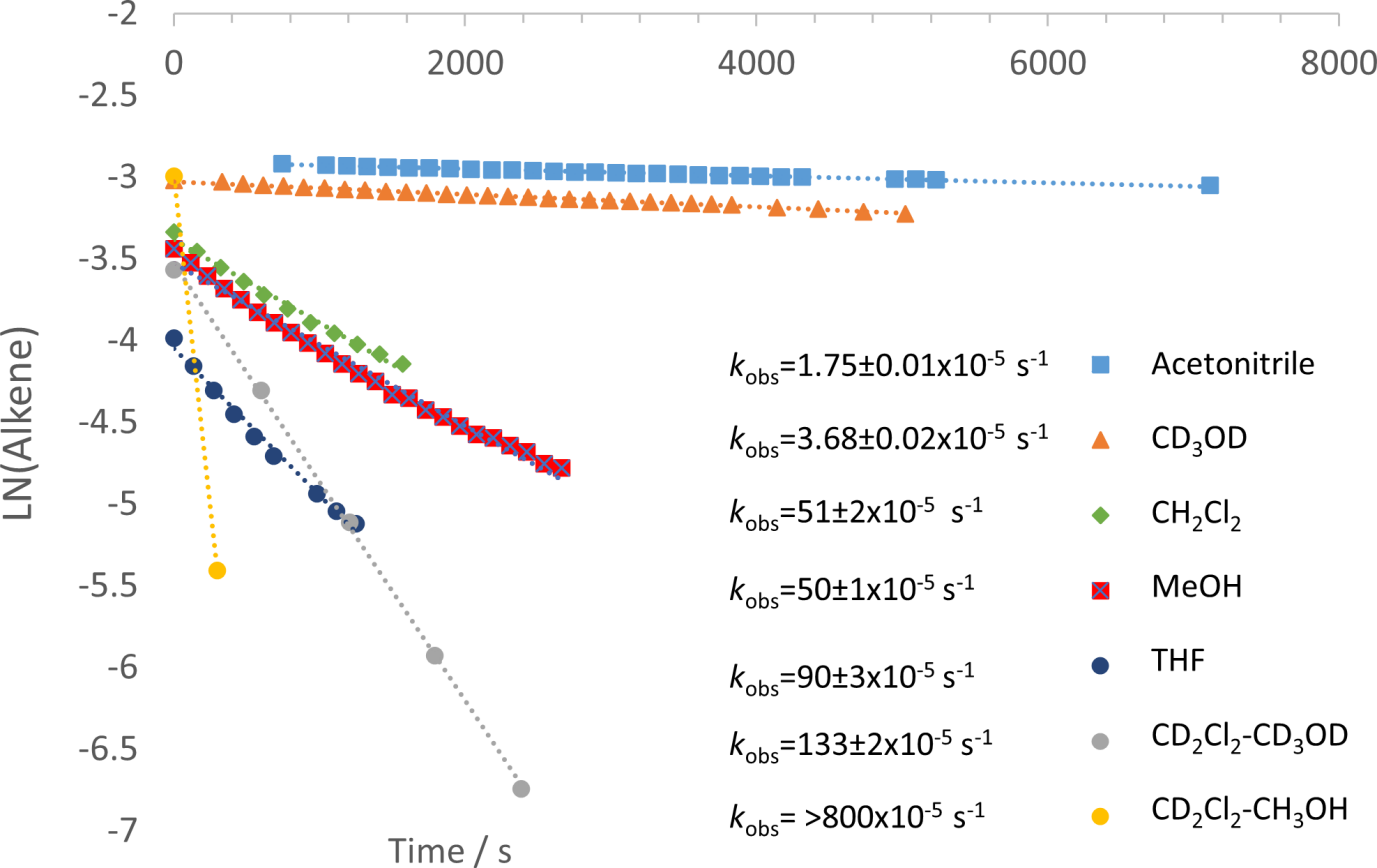
First order alkene decay for urea alkene **1a** (0.05 M) hydroamination with [JPhosAu(NCCH_3_)]SbF_6_ (**5**, 2.5 mol %) in various solvents.

For a screen of solvent effect on the rate of **1a** hydroamination to **3a**, commercially available ((acetonitrile)[(2-biphenyl)di-*tert*-butylphosphine]gold(I) hexafluoroantimonate (**5**) was used as catalyst (Figure 1), where gold is supported by the ligand commonly known as “Johnphos” or, henceforth, “JPhos”. Interestingly, there are minor differences in observed rate (first order fit in alkene decay) between THF (ε = 7.6, polarity index = 4.0), methylene chloride (ε = 8.9, polarity index = 3.1), and methanol (ε = 32.7, polarity index = 5.1) despite the large differences in solvent polarity (Table 2, entries 3, 4, and 5; rates in THF and MeOH are only 1–2 times faster than those in CD_2_Cl_2_). However, this did not hold true uniformly. When a commercially available electron-acceptor ligand known as “Jackiephos” (bis(3,5-bis(trifluoromethyl)phenyl)(2′,4′,6′-triisopropyl-3,6-dimethoxybiphenyl-2-yl)phosphine was used as the AuNTf_2_ salt (**6a**), in CD_2_Cl_2_, the reaction rate from **1a → 3a** was too fast to measure (reaction complete in *t* = 5 min, a >35 fold increase compared to MeOH, see Supporting Information File 1, Figure S12). Furthermore, the reaction rate with JackiephosAuNTf_2_ in pure MeOH was slightly slower than that of JPhosAuOTf in MeOH, suggesting a polar protic solvent minimizes ligand effects. Acetonitrile and deuterated methanol significantly decelerate the reaction (Table 2, entries 6 and 7; 10–20 times slower than in CD_2_Cl_2_), while a curious cooperative acceleration was observed when methanol or methanol-*d*_4_ were used in combination with methylene chloride (Table 2, entries 1 and 2; 4–22-fold increase in rate compared to CD_2_Cl_2_). We were unable to find any clear correlation between rate and a variety of solvent parameters; the success of mixed solvents suggests many factors are at play. Throughout our studies, we used CH_2_Cl_2_ and CD_2_Cl_2_ interchangeably; there is a slight difference in rate between the two (Table 2, entry 5, *k*_rel_ = 1.4 CH_2_Cl_2_ versus CD_2_Cl_2_). The slightly faster reaction in CH_2_Cl_2_ we believe can be attributable to different levels of H_2_O contaminant.

**Table 2:** Relative rates of hydroamination with [JPhosAu(NCCH_3_)]SbF_6_ (2.5 mol %) and urea alkene **1a** (0.05 M) in various solvents.

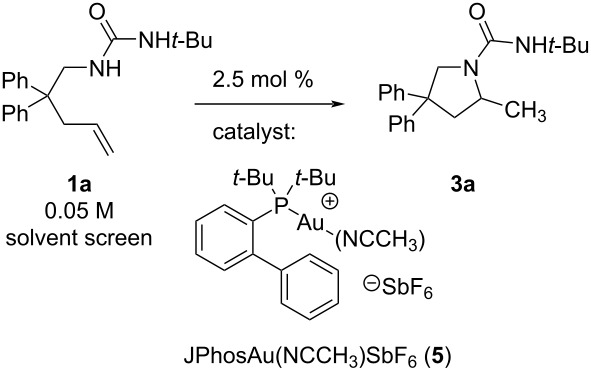			

Entry	Solvent	10^5^∙*k*_obs_/s^−1^	*k*_rel_

1	CD_2_Cl_2_/ 10% CH_3_OH	800 (estimate)	22
2	CD_2_Cl_2_/ 10% CD_3_OD	150 ± 23	4
3	THF-*d*_8_	87 ± 26	2.4
4	MeOH	50 ± 7^a^	1.4
5	CH_2_Cl_2_ CD_2_Cl_2_	51 ± 1 37 ± 1	1.4 1
6	CD_3_OD	4.2 ± 0.3	0.11
7	CD_3_CN	1.75 ± 0.01	0.05

^a^Additional trials with JPhosAuOTf.

**Figure 2:**
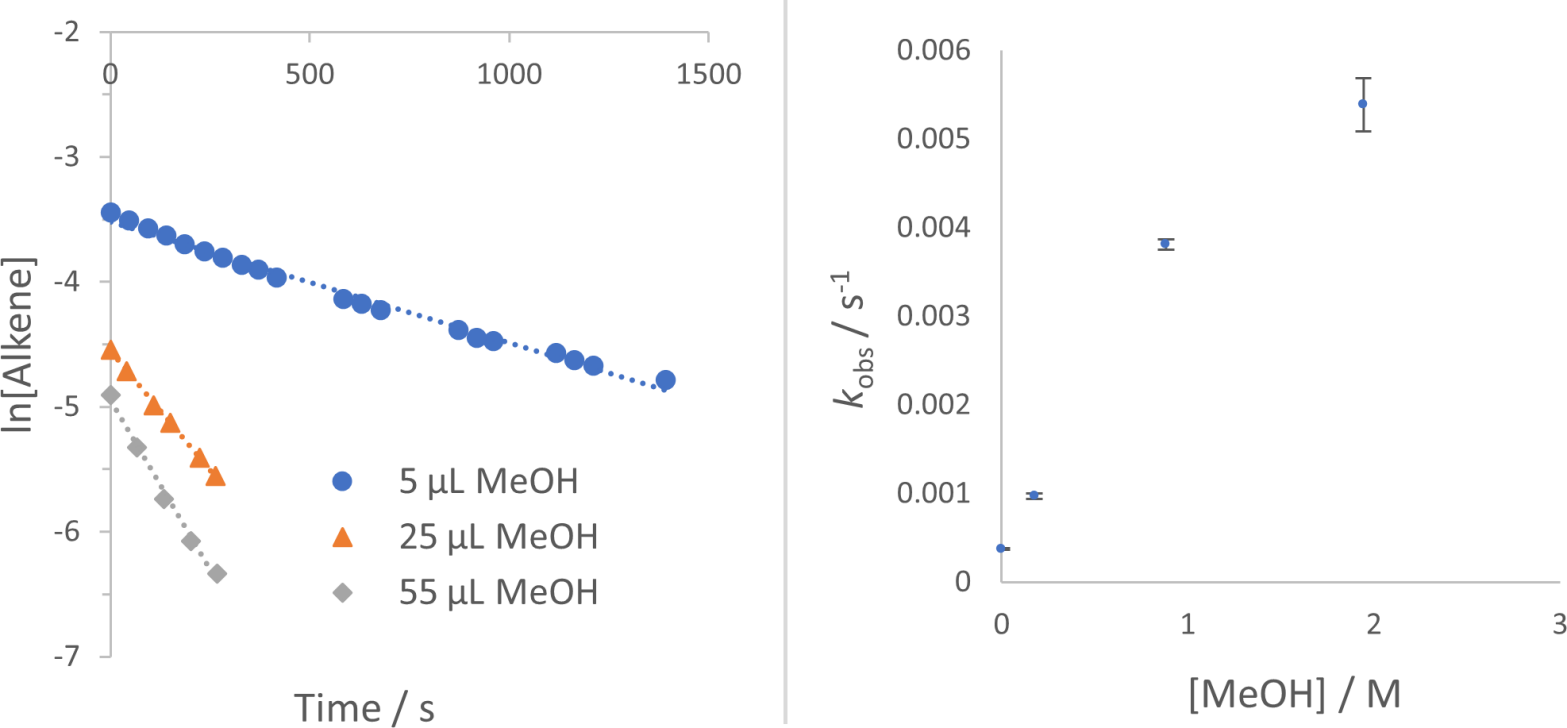
Cooperative effect of mixed CD_2_Cl_2_/MeOH on alkene **1a** → **3a** conversion with catalyst **5** (2.5 mol %). Error bars are from linear least squares analysis of raw data plots.

To quantify the cooperative accelerating effect of CH_3_OH combined with DCM in the reaction of **1a** → **3a**, we performed rate studies with titrated amounts of MeOH (Figure 2). In CD_2_Cl_2_ the rate of **1a** disappearance increases steadily with each increase of MeOH (from 0–55 μL, or 0.18 M–1.94 M). We expect rates to plateau and then decrease, since the rates in pure MeOH are slower (see above, Table 2, entry 4). We were not able to determine the maximum impact of added MeOH, because the rate of cyclization became too fast to be detectable by ^1^H NMR kinetics (*t* < 5 minutes). A LN-LN plot of MeOH concentration versus observed rate gives a slope of 0.7, indicating less than first order dependence on MeOH.

**Figure 3:**
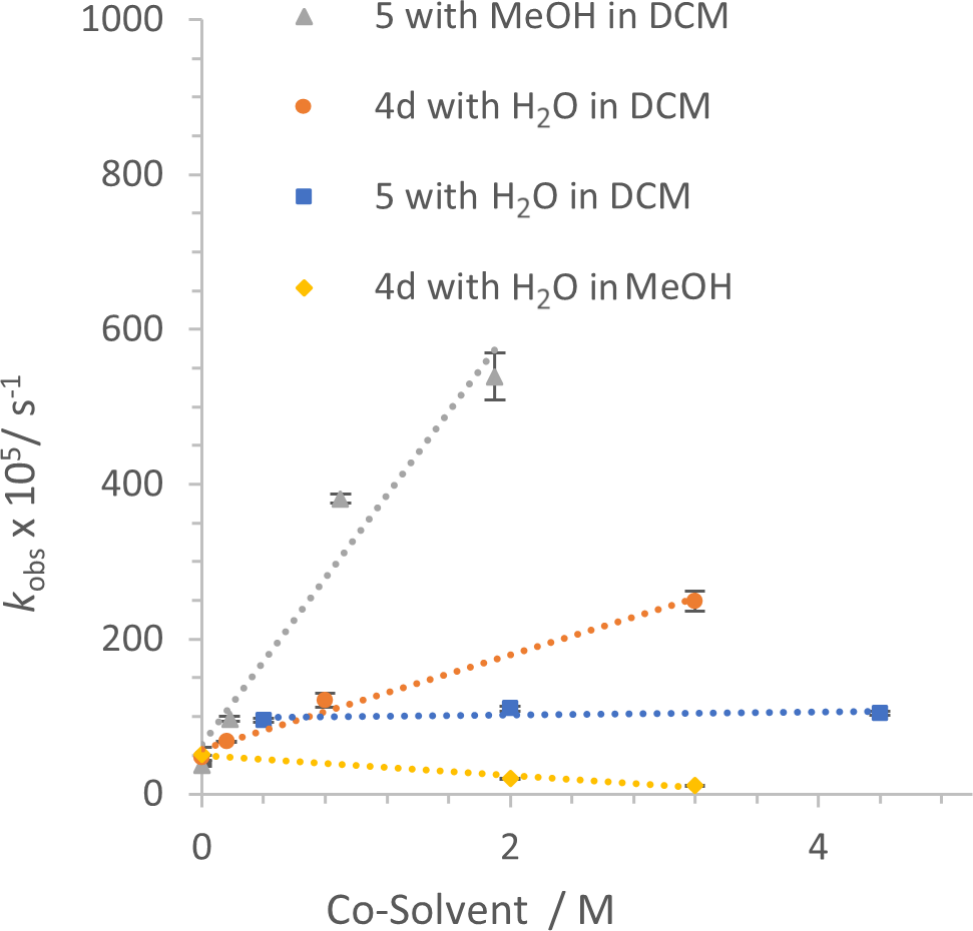
Different additive impact on rate of **1a** → **3a** depending upon catalyst and co-solvent. The data for JPhosAu(NCCH_3_)SbF_6_ with MeOH in DCM (▲) is reproduced in Figure 2. (The reaction with catalyst **5** and water in MeOH is not shown but displays similar inhibition). Error bars are from linear least squares analysis of raw data plots; where not visible they are smaller than the icon for the data point.

A similar acceleration in **1a** hydroamination is seen when water additive is combined with DCM solvent, but unexpectedly, subtle differences were observed depending upon the identity of the catalyst (Table 3, Figure 3). Early experiments used JPhosAuOTf (synthesized in our lab) and water as co-solvent, but the conditions were modified to use readily accessible commercial [JPhosAu(NCCH_3_)]SbF_6_ (**5**) and MeOH, since water posed miscibility problems at higher concentrations. Titrating increasing amounts of water into reactions with JPhosAuOTf (**4d**) led to an increase in rate, similar to that seen with MeOH and catalyst **5**, albeit smaller in magnitude (Table 3, entries 1–4 show the increasing rate with increasing water up to a 5.3-fold increase at 3.2 M water, while entries 5–8 show the increasing rate with increasing methanol up to a 14.6-fold increase at only 1.9 M methanol). In contrast, titrating increasing amounts of water into reactions of **1a** with [JPhosAu(NCCH_3_)]SbF_6_ (**5**) led to an initial boost that quickly plateaued (Table 3, entry 5 compared to entries 9–11). Furthermore, when water was added to the reaction catalyzed by JPhosAuOTf (**4d**) in MeOH, the reaction *slowed* with increasing amounts of water (Table 3, entries 12–14) [39]. The same decelerating effect of water in MeOH solvent was seen with [JPhosAu(NCCH_3_)]SbF_6_ (**5**) (Table 3, entries 15–17).

**Table 3:** Impact of titrated water or methanol into CD_2_Cl_2_ or CH_3_OH.

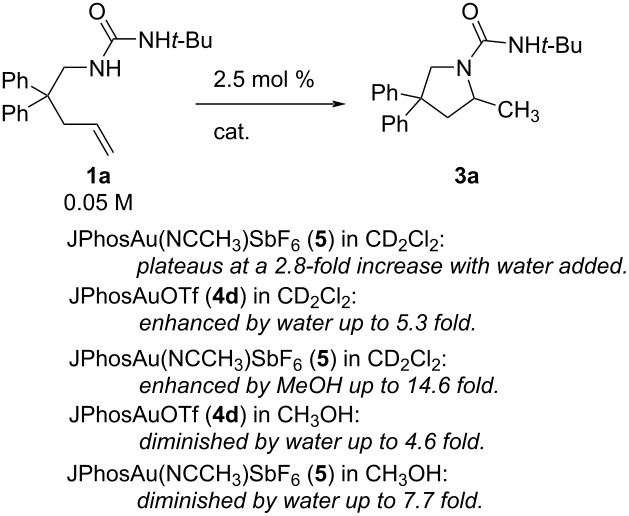			

	Cat.	Additive	*k*_obs_∙10^5^/s^−1^

Solvent CD_2_Cl_2_			

1	**4d**	0 M water	47 ± 3
2	**4d**	0.16 M water	67.4 ± 0.6
3	**4d**	0.8 M water	121 ± 9
4	**4d**	3.2 M water	249 ± 13
5	**5**	0 M	37 ± 1
6	**5**	0.18 M MeOH	97 ± 3
7	**5**	0.9 M MeOH	381 ± 6
8	**5**	1.9 M MeOH	539 ± 30
9	**5**	0.4 M water	95 ± 3
10	**5**	2 M water	110 ± 3
11	**5**	4.4 M water	104 ± 3

Solvent MeOH			

12	**4d**	0 M water	48.2 ± 0.8
13	**4d**	2 M water	19.5 ± 0.2
14	**4d**	3.2 M water	11.1 ± 0.4
15	**5**	0 M water	50 ± 1
16	**5**	2 M water	11.5 ± 0.3
17	**5** ^a^	6 M water	6.5 ± 0.4

^a^At 75 μL water in CH_3_OH precipitates begin to form and the sample is turbid.

### Substrate effect

The rate of hydroamination to cyclized **3a–c** was measured for three substrates, *tert*-butylurea **1a**, *tert*-butyl carbamate **1b**, and benzamide **1c** (Table 4). Under standard conditions (2.5 mol % [JPhosAu(NCCH_3_)]SbF_6_, 0.05 M alkene in DCM) benzamide and carbamate hydroamination were too slow to measure, so the reactions were done with 55 μL MeOH promoter but still only an estimated rate constant was obtained for **1c** (14% conversion after 24 h, estimated *t*_1/2_ = 96 h, *k*_obs_ = 1.4 × 10^−6^ s^−1^). With 55 μL MeOH in DCM, the relative rates for each substrate are 3,850:50:1 with urea **1a** > carbamate **1b** > benzamide **1c**. The analogous toluene sulfonamide substrate **1d** did not react on measurable timescales at room temperature (no product with up to 10 mol % JPhosAu(NCCH_3_)SbF_6_ in CD_2_Cl_2_ after 48 hours with and without added CH_3_OH) despite common use of sulfonamides in alkene hydroamination reports, albeit at higher temperatures.

**Table 4:** Relative rates of hydroamination with different protecting groups on nitrogen; (**1a**–**c**, 0.05 M) with catalyst **5** (2.5 mol %) in DCM and 55 μL CH_3_OH promoter (1.9 M).

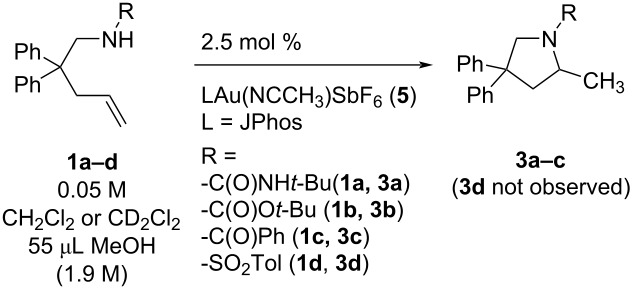		

Entry	Substrate	*k*_obs_∙10^5^/s^−1^

1	**1a**/CD_2_Cl_2_	539 ± 30
2	**1b**/CH_2_Cl_2_	6.9 ± 0.2
3	**1c**/CH_2_Cl_2_	≤0.14
4	**1d**/CD_2_Cl_2_	n.d.*

*No reaction with 10 mol % catalyst **5**.

Based on our ligand survey above, we proposed to improve rates of cyclization with slower reacting substrates by identifying a more Lewis acidic gold. To determine whether benzamide (**1c**) cyclization could be made efficient with appropriate combination of ligand and MeOH we surveyed rates with (PhO)P(*o*-biphenyl)_2_AuOTf (**4a**) and JackiephosAuNTf_2_ (**6a**, Table 5). Benzamide rates remain slow but gains can be achieved – benzamide (**1c**) cyclization with Jackiephos and 55 μL MeOH promoter matches the rate of cyclization of carbamate (**1b**) with JPhosAu(NCCH_3_)SbF_6_ with only 5 μL MeOH promoter (see Supporting Information File 1, Figure S18). Increasing to 100 μL MeOH did not increase the benzamide cyclization rate any further (see Supporting Information File 1, Figure S19). This comparison reveals that the Jackiephos supported gold accelerates the reaction more than (PhO)P(*o*-biphenyl)_2_ does, although this may be in part an anion effect [7]. The bis(trifluoromethyl)aryl substituent is expected to be more electron withdrawing than an *o*-biphenyl, so presumably Lewis acidity is boosted here.

**Table 5:** Benzamide (**1c**, 0.05 M) hydroamination with 2.5 mol % of two different catalysts in CH_2_Cl_2_ with 55 μL CH_3_OH.

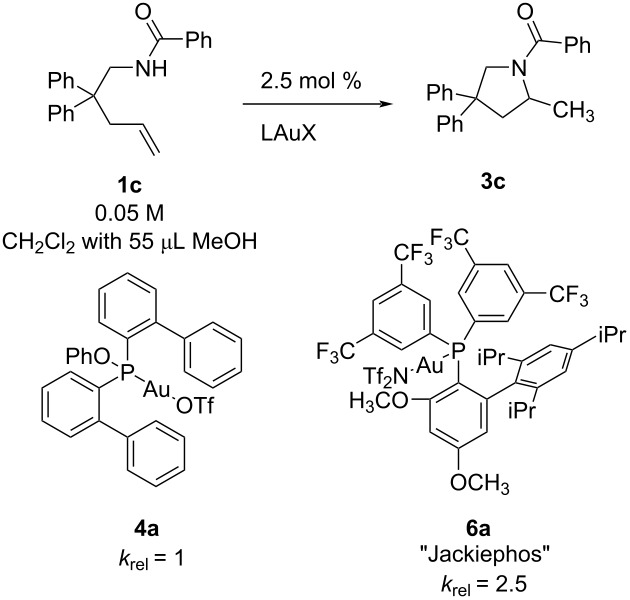		

Entry	Catalyst	10^5^∙*k*_obs_/s^−1^

1	**4a**	0.37 ± 0.02
2	**6a**	1.02 ± 0.09

### Observations on ligand effect and decomposition

Ligand effects on rates of hydroamination are amplified with slower reacting substrates and lower amounts of MeOH (higher relative amounts of bulk solvent methylene chloride). With the carbamate substrate **1b**, the rate of cyclization is 10× faster with PhO(*o*-biphenyl)_2_PAuOTf (**4a**) compared to *t*-Bu(*o*-biphenyl)_2_PAuOTf (**4c**) when no MeOH additive is used, and only ≈2× faster with 5 and 25 μL of MeOH (see Table 6). This contrasts the 3.6 fold difference in rate depending upon catalyst identity (*k*_(_**_4a_**_/_**_4c_**_)_) for urea substrate **1a** → **3a** and no MeOH (see Table 1).

**Table 6:** Influence of increasing MeOH and catalyst (2.5 mol %) on carbamate **1b** (0.05 M in CH_2_Cl_2_) reactivity.

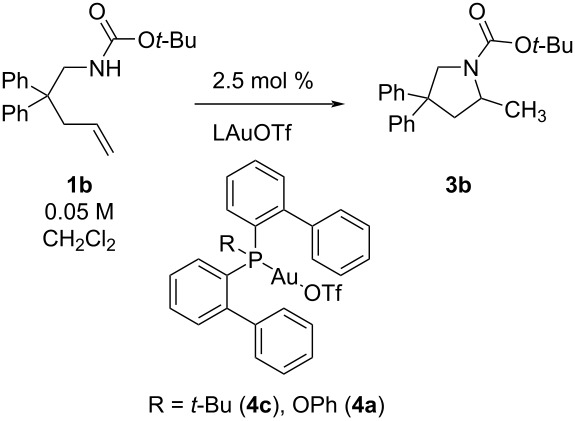			

Entry	Additive	Cat.	10^5^∙*k*_obs_/s^−1^

1	none	**4a** ^a^	1.981 ± 0.007
2	5 μL MeOH	**4a** ^a^	17.01 ± 0.08
3	25 μL MeOH	**4a** ^a^	21.7 ± 0.1
4	none	**4c**	0.205 ± 0.009
5	5 μL MeOH	**4c**	8.58 ± 0.01
6	25 μL MeOH	**4c**	12.68 ± 0.07

^a^Decomposition observed.

Hydroaminations with slower reacting substrates revealed another important factor. ^31^P NMR spectra during the cyclization of carbamate **1b** indicated significant amounts of catalyst decomposition when PhOP(*o*-biphenyl)_2_AuOTf (**4a**) was used, however, the appearance of decomposed catalyst was not sufficiently detrimental to halt the reaction altogether. Catalyst decomposition is typically detected in two ways, either by the appearance of diagnostic [L–Au–L]^+^ in the ^31^P NMR spectrum, or by observation of a kinetic plateau in the reaction rate [40]. In the reactions monitored here, the observed extent of decomposition is dependent on ligand and substrate. For example, in the cyclization of the less reactive carbamate substrate, with catalyst **4c** the decomposed product [L–Au–L]^+^ was not observed on the timescale of the reaction, whereas with catalyst **4a**, the decomposed product was observed. Also noteworthy, despite the higher amount of decomposition, ligand **4a** still created a more effective catalyst system (twice as reactive, see Table 6). Furthermore, observed decomposition did not correlate with a reaction plateau; when in the presence of 5 μL MeOH, the first order plots of alkene decay retained linearity up to 80% conversion, and separate ^31^P NMR experiments indicated significant decomposition at about 50% conversion. Independently prepared [L–Au–L]^+^ has been shown by others to be inactive catalysts (L = Ph_3_P) [41] and we confirmed this also with L = (*t-*Bu)_2_P(*o*-biphenyl). When the more reactive urea alkene **1a** is examined, the reaction with **4a** is efficient enough that reaction completion occurs prior to noticeable decomposition. In contrast, when a LAuNTf_2_ catalyst was prepared where L = tris(2,4-di-*tert*-butylphenyl)phosphite (**6b**), a supporting ligand that would be predicted to create more electrophilic gold due to its high π-acceptor properties, major decomposition was observed for the slower substrates (**1b** and **1c**) and the fast urea (**1a**), indicating catalysts that are much more prone to decomposition, and preventing any meaningful determination of an actual ligand effect on reactivity (see Supporting Information File 1, Figures S25 and S26). These experiments indicate that with highly reactive catalysts, productive reactions can take place despite a drop in concentration of non-decomposed catalyst. The true reactivity of such catalysts may thus be anticipated to be much higher than the actual rates measured.

**Figure 4:**
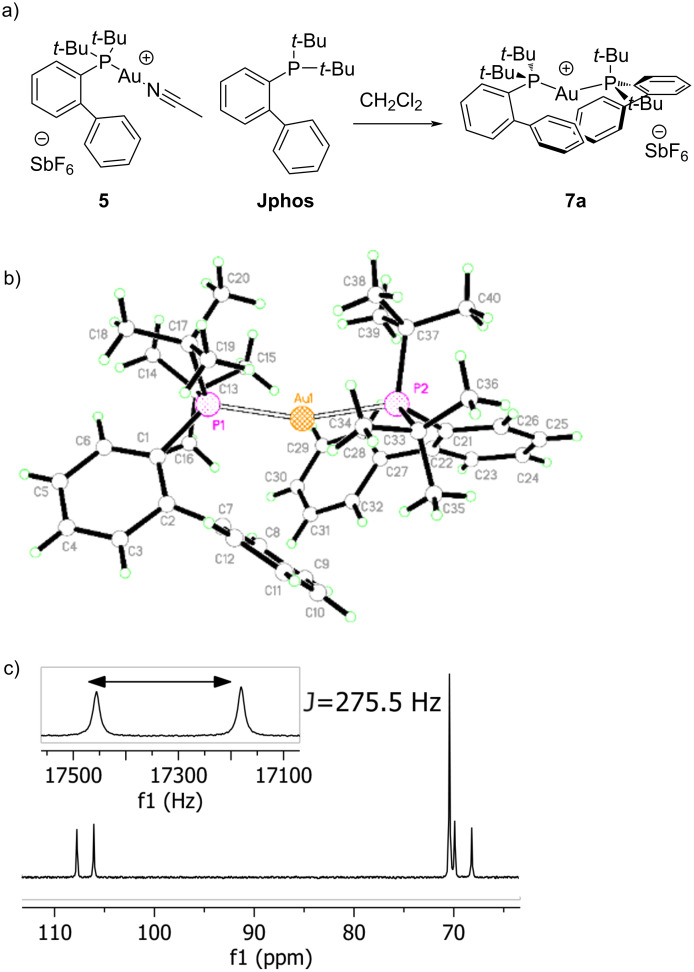
(a) Schematic for synthesis of [L–Au–L]SbF_6_ where L = JPhos. (b) Perspective drawing of the cation in crystalline [Au(P(C_4_H_9_)_2_(C_12_H_9_))_2_](SbF_6_)CH_2_Cl_2_ where P are represented by dotted spheres, Au atoms are represented by cross-hatched spheres, and carbon and hydrogen atoms are represented by medium and small open spheres, respectively and all nonhydrogen atoms are labeled [43]. (c) ^31^P NMR spectrum (161.98 MHz, CDCl_3_).

We noted recently that while the [Ph_3_P–Au–Ph_3_P]^+^ decomposition product makes a multitude of appearances in the literature, the corresponding complex has not be reported for JPhos, therefore we sought to independently prepare it. The structure ended up being unique and puzzling. When [LAu(NCCH_3_)]SbF_6_ was treated with an equivalent of free ligand, a complicated ^31^P NMR spectrum was acquired which we at first believed to be a result of erroneous choice of free ligand! Three signals were observed, a set of doublets at 107 and 69 ppm, with *J* = 275.5 Hz, and a singlet at 70.5 ppm (Figure 4c) [42]. In all attempts to prepare bisphos **7a**, the singlet and set of doublets were observed, and always in the same approximate ratio. X-ray crystallographic analysis shows C2 rotational symmetry and confirms the identity of complex **7a**. Our preliminary hypothesis is that two conformations exist in solution, one of which is symmetrical (presenting as a singlet), one of which is not. A lack of symmetry in one conformation would mean that each phosphorous is magnetically inequivalent, and thus shows splitting to the other in the NMR spectrum. Although we observed reaction inhibition in a number of instances with [JPhosAu(NCCH_3_)]SbF_6_ we have never yet observed the formation of this byproduct. On the one hand, this is a testament to the high stability of gold supported by JPhos, on the other hand, it suggests as yet undetermined deactivation pathways. A similar ^31^P spectrum was obtained for the bisphoshine complex of Jackiephos, but in contrast no symmetrical singlet was observed (see Supporting Information File 1, Figure S23).

### Kinetic deuterium isotope effect

**Figure 5:**
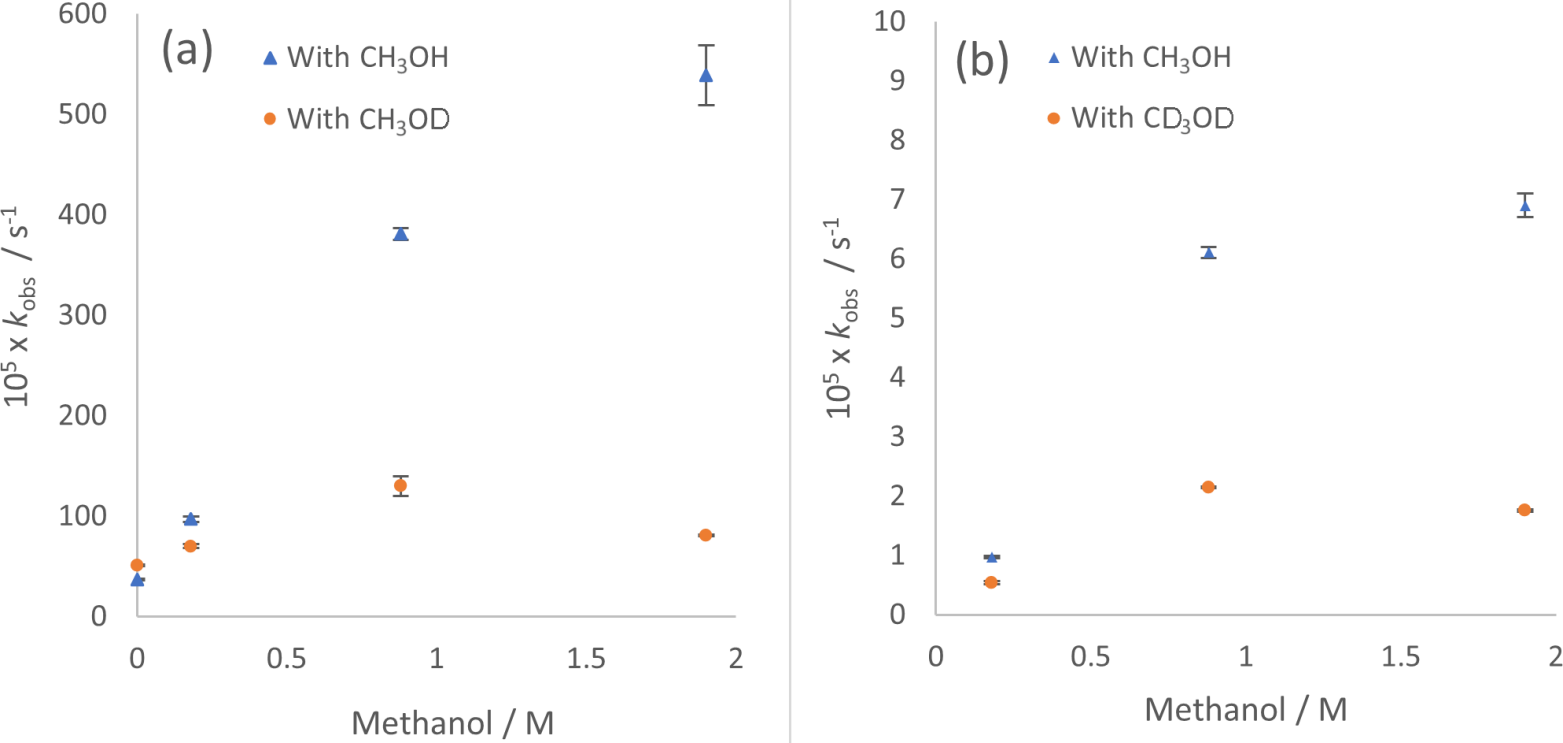
(a) *k*_obs_ for reaction of urea **1a** (0.05 M) in DCM with catalyst **5** and titrated CH_3_OH/CH_3_OD. Data for CH_3_OH reproduced in Figure 2, Table 3. (b) *k*_obs_ for reaction of carbamate **1b** (0.05 M) in DCM with catalyst **5** and titrated CH_3_OH/CD_3_OD. Error bars are from linear least squares analysis of raw data plots; where not visible they are smaller than the icon for the data point.

Monodeuterated methanol (CH_3_OD) was tested as an additive on the cyclization rate of alkene urea **1a** with JPhosAu(NCCH_3_)SbF_6_ (**5**) in CH_2_Cl_2_ (Table 7, Figure 5). In this case an initial boost in reactivity was observed with 5 and 25 μL of CH_3_OD, followed by a drop-off in reactivity at 55 μL (even slower than in pure CH_2_Cl_2_). In all three experiments, rapid H/D exchange (*t* < 5 minutes) reduced the N–CH_2_ doublet (δ 3.84 ppm in CD_2_Cl_2_) to a singlet, indicating high incorporation of deuterium into the substrate and corresponding in situ generation of CH_3_OH. Furthermore, both NH signals appear absent in the ^1^H NMR spectrum (δ 3.93/3.66). Comparing the rates of reactivity to those with CH_3_OH as additive provides a range of KIE values, *k*_(H/D)_ = 1.4 to 6.6. When the bulk solvent was changed from pure CH_3_OH to CD_3_OD a solvent isotope effect of 11.9 was measured.

**Table 7:** Influence of MeOH/MeOD on urea and carbamate reactivity.

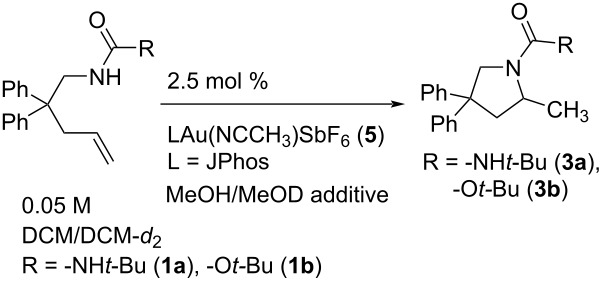			

Entry	Substrate/ solvent	Additive	*k*_obs_∙10^5^/s^−1^

1	**1a**/CH_2_Cl_2_**1a**/CD_2_Cl_2_	n/a	51 ± 1 37 ± 1
2	**1a**/CH_2_Cl_2_	0.18 M MeOD	70 ± 2
3	**1a**/CH_2_Cl_2_	0.88 M MeOD	130 ± 10
4	**1a**/CH_2_Cl_2_	1.9 M MeOD	81 ± 1
5	**1a**/CD_2_Cl_2_	0.18 M MeOH	97 ± 3
6	**1a**/CD_2_Cl_2_	0.88 M MeOH	381 ± 6
7	**1a**/CD_2_Cl_2_	1.9 M MeOH	539 ± 30
8	**1b**/CH_2_Cl_2_	0.18 M CD_3_OD	0.54 ± 0.02
9	**1b**/CH_2_Cl_2_	0.88 M CD_3_OD	2.15 ± 0.01
10	**1b**/CH_2_Cl_2_	1.9 M CD_3_OD	1.76 ± 0.02
11	**1b**/CH_2_Cl_2_	0.18 M MeOH	0.97 ± 0.02
12	**1b**/CH_2_Cl_2_	0.88 M MeOH	6.11 ± 0.09
13	**1b**/CH_2_Cl_2_	1.9 M MeOH	6.9 ± 0.2

Attempts to prepare deuterated urea alkene **1a**-*N*-d_2_, resulted in only partially deuterated material. Treatment of partially deuterated alkene urea **1a**-*N*-d_2_ (0.05 M in CD_2_Cl_2_) with JPhosAu(NCCH_3_)SbF_6_ (2.5 mol %) gave a *k*_obs_ = 48 ± 8 × 10^−5^ s^−1^ (which compared to *k*_obs_ = 37 ± 1 × 10^−5^ with non-deuterated **1a** under the same conditions) showed no primary isotope effect (*k*_(H/D)_ = 0.8). Known sensitivities to trace water in solution and observed scatter with impure **1a**-*N*-d_2_ suggests that this is not a true inverse effect. The observed KIE correlates with the concentration of protons in solution, and a potential slowing from D-incorporation may be tempered by trace water (H_2_O). In the MeOD titration experiments with **1a**, rapid N–H/D exchange results in MeOH generation in situ which would definitely counterbalance any deceleration from deuteron incorporation (see above). When rates of hydroamination of carbamate **1b** were measured with CD_3_OD, incorporation of deuterium was slower and less complete. With 5 μL CD_3_OD (0.18 M) 70% deuterium incorporation occurred whereas at 25 and 55 μL CD_3_OD (0.88 M and 1.94 M) 90–95% deuterium incorporation was observed (as detected by NH integration in the ^1^H NMR spectrum). Moderate KIE values were measured for **1b** (by comparing to experiments with titrated CH_3_OH), from *k*_(H/D)_ = 1.8 at 5 μL methanol to *k*_(H/D)_ = 3.9 at 55 μL methanol. Interestingly, faster overall reactions with urea **1a** correlate with faster nitrogen D/H exchange (*t* < 5 minutes), whereas the slower overall reaction with carbamate **1b**, correlates with slower D/H exchange (*t* ≈ 30–50 minutes).

Similarly, large solvent isotope effects are observed with different ligands and different substrates (Table 8). When gold supported by the more electron-withdrawing ligand Jackiephos, in the form of the NTf_2_ salt (**6a**), is used, the reaction is 7.4 times faster in CH_3_OH compared to CD_3_OD. When the slower reacting carbamate **1b** is used with JPhosAu(CH_3_CN)SbF_6_ (**5**), the reaction is 6.3 times faster in CH_3_OH compared to CD_3_OD. These results demonstrate very consistently large solvent isotope effects that appear to be independent of both ligand and substrate.

**Table 8:** Measured solvent isotope effects.^a^

Entry	Substrate	Cat.	*k*_(H/D)_

1	carbamate **1b**	**5**	6.3
2	urea **1a**	**5**	11.9
3	urea **1a**	**6a**	7.4

^a^Comparison of first order rate constants for disappearance of alkene **1a** or **1b** (0.05 M) in either CH_3_OH or CD_3_OD with different catalysts.

### Other additives

**Figure 6:**
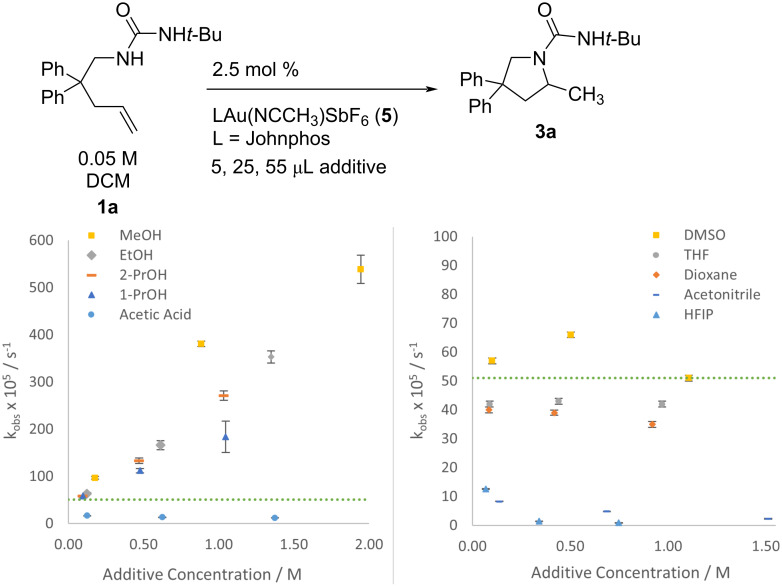
Rate of urea **1a** (0.05 M) hydroamination with JPhosAu(NCCH_3_)SbF_6_ (2.5 mol %) in CH_2_Cl_2_ with 5, 25, and 55 μL of additive; dotted line shows rate in pure CH_2_Cl_2_. Numerical observed rates are given in Supporting Information File 1. Error bars are from linear least squares analysis of raw data plots; where not visible they are smaller than the icon for the data point.

We sought to determine whether the accelerating effect of MeOH co-solvent on the **1a** → **3a** transformation was due to its role as a hydrogen bonding donor (proton source), or due to its role as a hydrogen bonding acceptor (Lewis base) [44]. To this end, we examined the impact of different alcohols (varied acidity and polarity) and different non-protic Lewis bases as additive to the bulk CH_2_Cl_2_ solvent (Figure 6) and compared them to the baseline rate in the absence of additive. The rates of formation of **3a** are mildly sensitive to alcohol structure with MeOH outperforming EtOH and propanol. For the set of linear alcohols, the shorter the chain, the faster the reaction. With a more strongly Bronsted acidic additive (acetic acid), rates are diminished with increasing additive. With a more polar non-protic additive (DMSO) the rate initially increases, then decreases with possible catalyst decomposition observed. Ether additives THF and dioxane have a slight inhibitory effect that does not change significantly with concentration. Addition of 5 μL hexafluoroisopropanol (HFIP) slows the reaction. Additional HFIP disrupts catalytic reactivity almost completely; none of the expected product **3a** was detectable after 1.6 hours in the presence of 55 μL HFIP. Acetonitrile is similarly detrimental to reaction rates. As discussed above, decomposition with catalysts supported by Ph_3_P show a diagnostic peak in the ^31^P NMR spectrum for (Ph_3_P)_2_Au^+^ (45 ppm). Deactivation with HFIP does not reveal a peak for the bisphosphine complex **7a**, so we are uncertain of the mechanism of deactivation here.

### Order in substrate and catalyst, activation parameters

**Figure 7:**
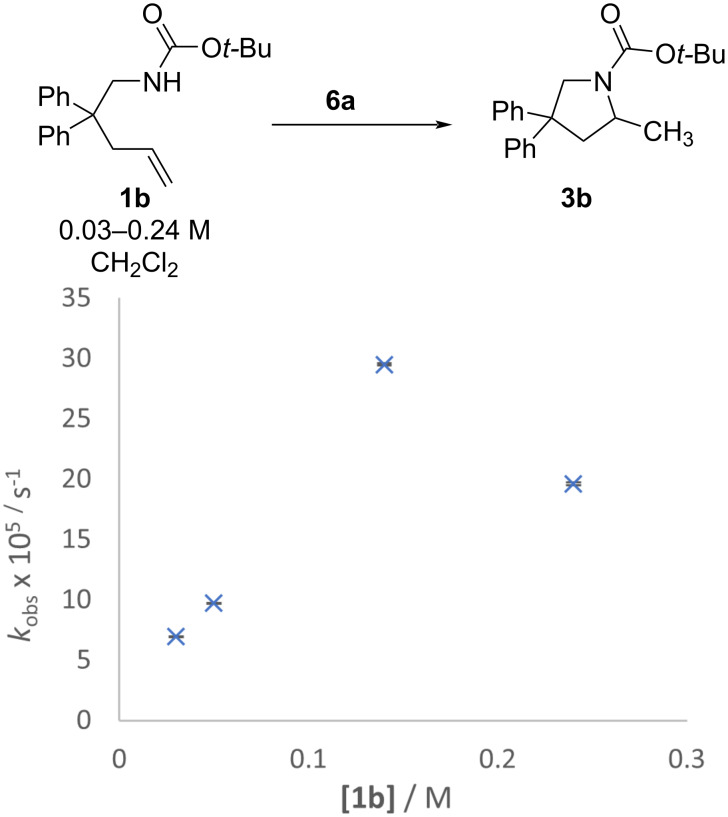
Observed rates for the reaction of carbamate **1b** (0.03–0.24 M) with JackiephosAuNTf_2_ (0.0013 M, **6a**) in CH_2_Cl_2_ to form **3b**. Error bars are from linear least squares analysis of raw data plots; where not visible they are smaller than the icon for the data point.

Attempts to determine reaction orders and full kinetic details for **1** → **3** were challenging. With respect to substrate concentration, all above rates are reported as first order rate constants for disappearance of alkene (*k*_obs_). All of our reactions fit linear plots of alkene decay, but almost invariably, a slow-down period was observed at high conversions. For example, a typical experiment with urea alkene **1a** will show first-order plots that are linear up to 60–80% conversion, and first order plots in the early linear period show faster observed rates than in the later slower period. A similar effect was attributed to catalyst decomposition in propargylamide cyclization [45]. We found this behavior mimicked second order decay (see Supporting Information File 1, Figure S38). Separate experiments where the concentration of alkene urea **1a** was varied with constant concentration of JPhos catalyst **5** do not support the conclusion that the reaction is second order in alkene. Unfortunately, inconsistent results were obtained and data indicated 0 to <1st order dependence on the concentration of **1a**. Furthermore, at high concentrations of **1a**, solutions became turbid as the substrate became less soluble, thus limiting the range of concentrations that could be used for relative rate plots. Switching to carbamate **1b** alleviated the solubility problem, but the significantly slower reactions required switching to a more reactive catalyst. With the more reactive Jackiephos-based catalyst **6a**, first order dependence on alkene concentration was observed when carbamate **1b** concentration was in the 0.03–0.133 M range, but then the rate dropped at 0.24 M carbamate (Figure 7).

**Figure 8:**
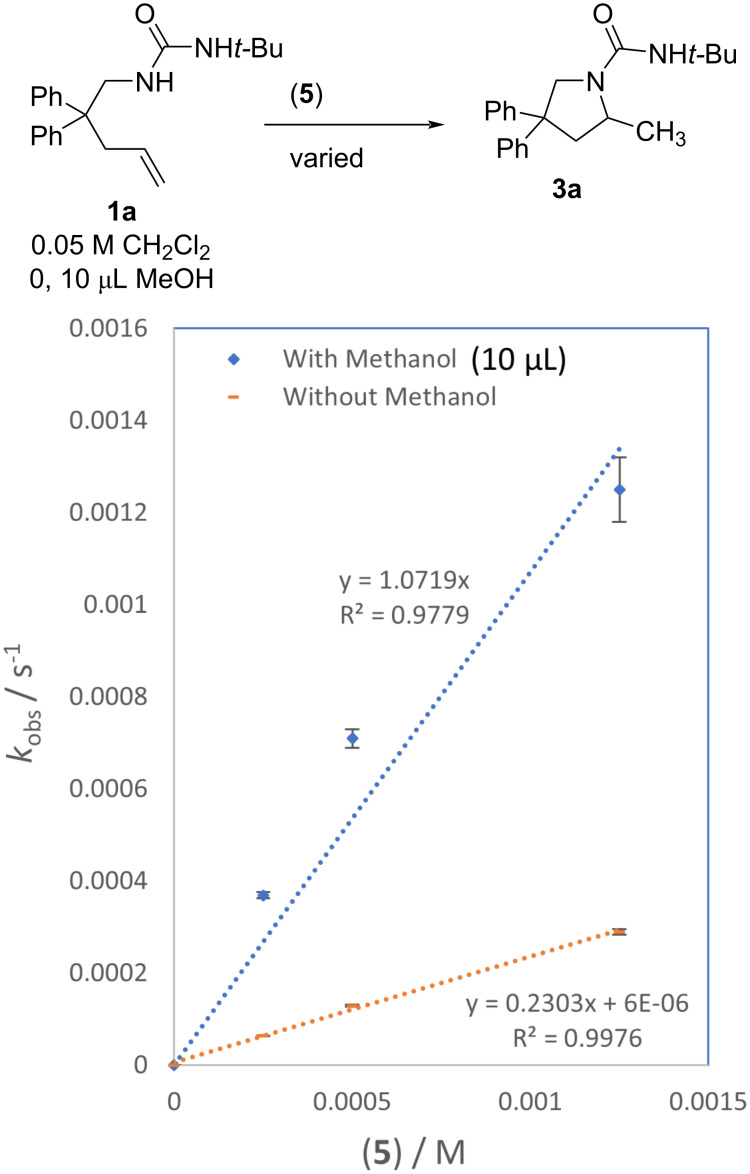
Influence of catalyst **5** concentration on rate of **1a** (0.05 M in CH_2_Cl_2_ with 0, 10 μL MeOH). Error bars are from linear least squares analysis of raw data plots; where not visible they are smaller than the icon for the data point.

The impact of catalyst concentration on the cyclization of urea alkene **1a** (0.05 M, CH_2_Cl_2_) was tested with no added MeOH and with 10 μL of added MeOH (Figure 8). The catalyst loading was varied from 0.5 mol % to 2.5 mol %. In the absence of MeOH, the reaction showed a first-order dependence on catalyst concentration. In the presence of MeOH, the dependence was non-linear and less than first order in catalyst. The impact of catalyst concentration on cyclization of **1a** was also determined in CD_3_OD (0.05 M) with 2.5, 10 and 20 mol % catalyst **5**; first order dependence on catalyst concentration was determined (see Supporting Information File 1, Figure S37).

Under the typical relatively dilute conditions in this study for **1a** (0.05 M) and in the absence of added MeOH, the data is consistent with a second order rate law rate = *k*_2_[alkene][gold catalyst]. To determine activation parameters rates of urea **1a** hydroamination were measured with 1 mol % [JPhosAu(NCCH_3_)]SbF_6_ (**5**) catalyst in DCM (0.05 M) at −5, 5, 15, and 30 °C. Second order rate constants were calculated by dividing *k*_obs_ by the catalyst concentration, as previous experiments indicated first order dependence on catalyst concentration. An Eyring plot of these data provided activation parameters Δ*H*^‡^ = 5.4 ± 1.1 kcal/mol and Δ*S*^‡^ −42.1 ± 3.9 cal/mol·K. Non-linear behavior in the presence of methanol indicated a more complex rate law (less than first order in methanol and catalyst), precluding determination of activation parameters under those conditions at this time.

## Discussion

The first reports of gold(I)-catalyzed alkene hydroamination were intermolecular additions by He [15–16] and intramolecular additions by Widenhoefer [12–13] in 2006, each catalyzed by phosphine ligand supported gold triflate (Ph_3_PAuOTf). Shortly after, arguments were made that reaction profiles were indistinguishable from those catalyzed by triflic acid and the gold π-activation pathway was questioned [26]. Nevertheless, advancements in gold-catalyzed reactions continued to be achieved. In particular, successful asymmetric methods were reported in short time after initially reported non-asymmetric methods, specifically Kojima’s tropos BIPHEP-gold(I)-catalyzed hydroamination of alkenylureas in 2012 [9]. Michon [5–8,10] and Widenhoefer continued to make advancements in asymmetric intra- and intermolecular variants, and unique solvent and anion dependencies continue to be examined from a theoretical standpoint.

For example, comparing intermolecular to intramolecular hydroamination, Widenhoefer and co-workers found opposite trends for efficient asymmetric induction: the intermolecular variant requires non-polar aromatic solvents while the intramolecular variant requires polar solvents [3]. Agbossou-Niedercorn, Michon, and co-workers found a reversal of enantioselectivity that can be controlled by choice of solvent, which they rationalized by invoking a solvent induced change in ion-pairs [6]. In another study by Agbossou-Niedercorn, Michon, and co-workers, they found that chiral alcohols do not impact enantioselectivity [7]. Protic additives are widely accepted to “facilitate proton transfer,” but they can also influence the aggregation of charged intermediates.

Most mechanistic discussions incorporate protodeauration of alkylgold intermediates and consider a continuum from rate or enantio-determining nucleophilic attack/alkene π-activation to rate or enantio-determining protodeauration (see Scheme 1). A mechanism study from Navarro and co-workers reinforce the need for bulky phosphine ligands to stabilize cationic gold, and in the intermolecular hydroamination of ethylene with imidazolidine-2-one they observed zero-order dependence on alkene, first-order dependence on catalyst, and second-order dependence on the imidazolidinone nucleophile [22]. They observed a primary kinetic isotope effect (*k*_(H/D)_ = 3.14) when deuterated amine was used. The second order dependence is rationalized by a mechanism where a second molecule of amine delivers a proton to the alkylgold intermediate (for protodeauration). In a platinum-catalyzed hydroamination, a protodemetalation pathway is supported by kinetics and reactivity studies on generated platinum alkyl intermediates [46]. In a palladium-catalyzed hydroamination, a protodemetalation pathway is also supported by kinetics and reactivity studies on generated palladium alkyl intermediates [47]. Formation of alkylgold intermediates is known to proceed with *anti*-attack [27] and the stereospecificity observed in deuterium-labeled intermediates was used as an argument for protodeauration pathways [15]. Widenhoefer, however, then showed that even HOTf acid catalyzed hydroaminations proceed with *anti*-selectivity [31]. In contrast, additions of water and indoles to alkenes are proposed to proceed via a Lewis acid-assisted Bronsted acid mode and computations suggest that gold is not electrophilic enough to activate alkenes toward the attack of pyrroles [34–35].

**Scheme 2:**
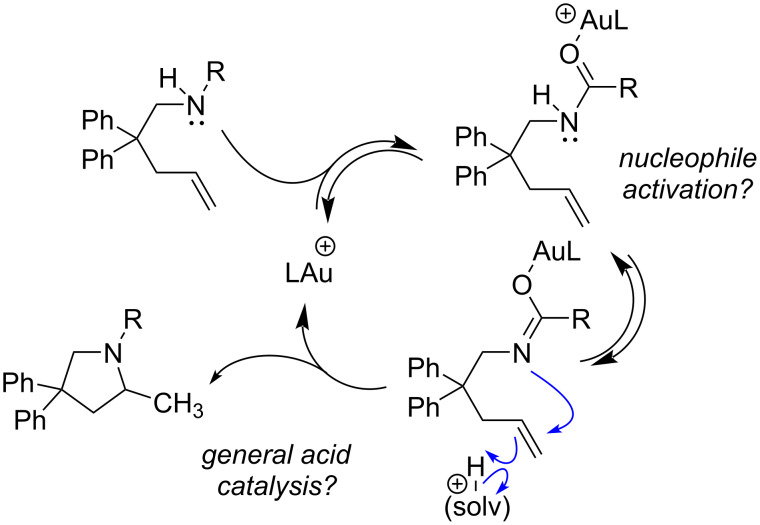
Proposed alternate mechanism.

Gold-catalyzed reactions are known to be sensitive to subtle anion and media effects [48], and within the binary of rate determining π-activation versus protodeauration, trends do not always provide obvious conclusions. The unique solvent and anion effects add complexity to mechanistic interpretation, and the reluctance of alkylgold complexes to undergo protodeauration under similar conditions give us pause [28]. While computational studies support protodeauration, the significantly lower reactivity of alkenes compared to alkynes and allenes continues to seek explanation [49]. Some of the observations reported here are not consistent with others while some are consistent and add quantitative detail and each aspect is summarized individually below [6–7]. Although the results may be interpreted within the π-activation/protodeauration paradigm for gold catalysis, we propose the data is also consistent with a mechanism involving gold-mediated tautomerization to release a proton, and concerted nucleophilic attack/proton transfer to the alkene (Scheme 2).

**Substrate effects:** Substrate trends in 5-*exo-trig* alkene hydroamination may have been masked by the high temperatures and long reaction times of early reports. For example, Widenhoefer engaged carbamates and amides in dioxane at temperatures >80 °C [12–13]. Later work showed that ureas could be engaged at room temperature when a NHC–gold catalyst system was used, outpacing acetamides, which in turn outpaced carbamates [14]. In work by Michon and co-workers, a substrate survey in TCE at 80 °C revealed that tosylsulfonamide **1d** was reactive, while the corresponding acetamide was not, and carbamates were seen as especially reactive, privileged substrates [8]. Our results are not fully consistent with either previously reported trend. Urea **1a** reacts faster than carbamate **1b**, which in turn reacts faster than benzamide **1c**. One qualitative interpretation is that rates are correlated with the availability of the attacking nitrogen lone pair. The urea nitrogen is expected to be most localized due to the strong competitive carbonyl resonance donation of the non-attacking nitrogen. Weaker competitive donation from the more electronegative oxygen, and the less electron-donating phenyl, would lead to decreasing nucleophilicity, respectively, for the carbamate and benzamide attacking nitrogen. Thus, the rate of intramolecular hydroamination is enhanced by a more nucleophilic nitrogen. Another qualitative interpretation is that rates are enhanced by carbonyl basicity. Gas-phase basicity measurements indicate that ureas are more basic than amides [47,50], and here the most Lewis base substrate is the most active. In 2012, Kojima and Mikami utilized bimetallic tropos BIPHEP [bis(phosphino)biphenyl]–digold complexes for enantioselective intramolecular hydroamination of *N*-alkenylureas [9], and they hypothesized that *N*-alkenylureas could be activated through bimetallic coordination not only with alkene but also the urea carbonyl. The Bronsted acidity of the urea would be increased by coordination to gold, and if such coordination is key to enabling reactivity, this would confirm the higher reactivity of urea **1a**. The divergent behavior of sulfonamide **1d** does not find an easy explanation; there are similarities and differences in the way a sulfonamide or carbonyl impacts a neighboring nitrogen. Sulfonamides have different steric profiles from carbonyls [51]. According to Roush et al. the electron-withdrawing capability of the S(O_2_)Ph group is in between that of the C(O)Me and CO_2_Me groups as measured by rates of Michael addition [52]. This suggests that in fact nitrogen nucleophilicity alone is not the most relevant factor since sulfonamides react much slower in hydroamination. Sulfonamide N–H bonds are significantly more acidic than urea, amide and carbamate N–H bonds (16.1 versus 23–27) [53], thus rates do not correlate with N–H acidity either (the least acidic urea is the most active).

Interaction of gold(I) with the two functional group types (carbonyl versus sulfonamide) is predicted to differ in a key aspect. Lithiated carbanions alpha to carbonyl functional groups are O-centered while being C-centered in analogous anions alpha to sulfonamides [51]. Sulfonamides are known to protonate at nitrogen [31]. Gold coordination to carbonyls would be predicted to retain N-centered nucleophilicity, while gold coordination to the sulfonamide nitrogen would not. Sulfonamides are less effective in reactions that depend on hydrogen bonding activation [54], and the substrate effects observed here find some similarities in other Bronsted acid-catalyzed processes. In disulfonimide-catalyzed asymmetric intramolecular hydroamination of alkenyl thioureas, sulfonamides are unreactive compared to thioureas and the reaction is favored at lower concentrations, an impact proposed to be due to hydrogen bonding driven self-association of the substrate [55]. In other Bronsted acid-catalyzed processes, thioureas and ureas are both effective substrates, with higher reactivity associated with thioureas. Carbamates, sulfonamides, and amides display significantly reduced reactivity, if any [56–57].

Titration experiments with MeOH-*d*_4_ (further discussion below) reveal that overall reaction rates for urea **1a** and carbamate **1b** appear to correlated with rates of gold-mediated N–H/D exchange which may further support the argument that faster reactions are associated with greater kinetic interaction with the carbonyl nucleophile. Sulfonamide hydroamination has also distinguished itself from urea and carbamate hydroamination in demonstrating productive reactivity with HOTf as a catalyst [31,58]. A few things are apparent from others’ HOTf control studies. First, a “simple” Bronsted acid-catalyzed mechanism that is possible with sulfonamides (i.e., with HOTf catalyst) is not operating here. Second, more complex acid-mediated rearrangements observed with ureas (authors propose a hetero-ene mechanism with concerted attack and protonation [59]) may be being mimicked by the gold-catalyzed processes here, further supporting the viability of a mechanism like that shown in Scheme 2. Finally, at its extreme, increasing nitrogen basicity inhibits gold catalytic activity, but within this low basicity regime, the opposite effect is observed [60].

**Ligand effects.** Early studies identified two key strategies for improving alkene hydroamination, switching from an electron-withdrawing catalyst (Ph_3_PAuCl/AgOTf) to an electron-donating catalyst (IPrAuCl/AgOTf (IPr = 1,3-bis(2,6-diisopropylphenyl)imidazol-2-ylidene), or by utilizing a dinuclear gold phosphine. These enhancements are likely correlated with increased catalyst stability. The earliest reports of alkene hydroamination revealed NHC’s and JPhos (*t*-Bu_2_)P(*o*-biphenyl) as the most active ligands [12–14], but this was in comparison to Ph_3_P which has since been understood to be especially prone to decomposition [20]. Further enhancement of stability can be achieved by utilizing tris(biphenyl)phosphines, thus enabling efficient intermolecular hydroamination [22]. Our results reveal the optimal ligand demands for alkene hydroamination – phosphines with electron-withdrawing substituents accelerate the reaction. Within the binary paradigm of gold-catalyzed mechanisms, where either π-activation or protodeauration is rate limiting, this electronic effect would suggest rate limiting π-activation [40], since protodeauration is fastest with strong donor ligands (*tert*-butylphosphines and NHC’s) [61], however, this qualitative interpretation is not necessarily diagnostic. Electron-withdrawing ligands would also support proton release from the nucleophile, by leading to higher concentrations of gold-coordinated carbonyl and a more acidified nucleophile (consistent also with the mechanism in Scheme 2).

**Solvent effects:** Michon and co-workers reported the beneficial assistance of water on enantioselective hydroamination, with the perchlorate being the best performing counterion. They propose that the anion is acting as a salting-in agent to contribute to better solvation of catalyst and organic molecules [6]. Our results here are consistent, but the dynamics of mixed solvent systems are complex – water in DCM enhances rates, while water in MeOH nearly shuts them down. We presume these are consistent with the solubility effects proposed by Michon. In DCM, protic co-solvents were uniquely beneficial – no other additives worked as well. Strategies to improve gold catalysis often center on enhancing protodeauration, and in studies of a vinylgold intermediate, HFIP was capable of mediating protodeauration while acetic acid was not [62]. Neutral alcohols are not acidic enough to protodeaurate alkyl or vinylgold intermediates [29]. HFIP has been shown to facilitate reactions with cationic intermediates and is proposed to activate by promoting formation of hydrogen bonding clusters with a catalyst [63]. Nolan and co-workers have shown that HFIP can be used to activate L–Au–Cl catalysts, precluding the need to use silver salts to generate a cationic catalyst with a weakly coordinating anion [64]. It is increasingly being tested as an additive in gold reactions, sometimes to beneficial effect [65], sometimes to neutral effect [22]. We are somewhat surprised by the detrimental effects seen here. Previous discussions of solvent effects focus on the ability to support or disrupt substrate metal interactions. Acetonitrile was shown to coordinate to gold more strongly than C–C multiple bonds, thus highly disfavoring the necessary π-coordination to initiate reactivity, and acetonitrile behaves accordingly here [66].

Specific gold–oxygen interactions are typically not invoked in mechanistic discussions, though a gold alcohol complex has been proposed in silyl enol ether protonation [67]. Equilibrium studies by Maier et al. indicate that methanol is more weakly coordinating than alkynes, acetonitrile, and the triflate counterion [66]. Theoretical calculation of binding constants predict that esters coordinate more strongly than alkynes, and water [68]. Alkenes have been shown in some cases to coordinate more strongly than alkynes, but the trend depends on substitution pattern (alkynes can coordinate more strongly) [69]. A more recent study of alkyne activation demonstrates that esters coordinate relatively weakly compared to alkynes, while amides coordinate relatively strongly [70]. Analysis of ^31^P chemical shift of the catalyst under reaction conditions does not provide strong conclusions about what is coordinating to gold in solution as chemical shifts vary only slightly compared to that of the original catalyst, but that does not preclude a kinetically effective catalyst cycle being initiated by gold coordination to functional groups in solution other than the alkene itself, thus deviating from classic π-activation. Solvent effects that subtly impact solubility and proton transfer processes are likely to mask the impact of individual mechanistic steps involving gold and are not adequate to distinguish between the contrasting mechanisms under consideration here (Scheme 1 vs Scheme 2). Further, we do not have an explanation for the special ability of protic co-solvents to accelerate hydroamination; THF should serve as a proton transfer agent in the same manner as MeOH according to the mechanism in Scheme 2; this suggests that hydrogen bonding clusters are involved in proton delivery.

**Reaction order and activation parameters.** Ureas are known to self-assemble, so their rates may be particularly sensitive to concentration and solvent variations and this may explain the range observed for order in substrate (from 0 to 1st). Furthermore, increasing amounts of MeOH also appear to shift the mechanism to a <1^st^-order dependence on catalyst, which could indicate a competing alcohol or H-bonding driven mechanism. In CD_3_OD, first-order-catalyst dependence is maintained. The entropy of activation reveals an organized transition state (−42.1 cal/mol·K) which would be consistent with the mechanism in Scheme 2. It is similar to that of HOTf-catalyzed intramolecular sulfonamide hydroamination (−34 ± 5 cal/mol·K) [31] and different from that measured for intramolecular pyridone hydroamination (−6.2 ± 0.8 cal/mol·K) [11].

**Isotope effects.** The presence or absence of kinetic deuterium isotope effect is usually interpreted within the context of two rate limiting regimes, protodeauration (which would show a primary KIE) or nucleophilic attack/π-activation (which would be presumed not to). A range of values have been observed for gold-catalyzed reactions in the literature, from 1 or close to 1 [11,71–72], to 3–5 [22,73–74]. The lack of KIE measured here for deuterated **1a** thus presumes fast protodeauration*.* Deuterium KIE’s for intramolecular alkene hydroamination with *N*-protected amines such as the ones in this study, had not previously been quantified, but in Michon’s study of carbamate hydroamination (**1b**) a KIE of 2.4 is estimated based on their statement of percent conversions in MeOH (61%) compared to MeOH-*d*_4_ (25%) after 20 hours at 50 °C [7]. Large isotope effects (>7) have been seen in a number of organometallic reactions [75] and tunneling need not be invoked if proton transfer involves linear or nearly linear transition states [76]. Isotope effects can also be maximized when the acid strength is the same on both sides of the reaction [77]. On the one hand, small variations observed here with different substrates and ligands could reflect the modulation of acidity from the presence of more Lewis acidic gold (Jackiephos/catalyst **6a**) or more weakly nucleophilic substrates (carbamate/**1b**) (Table 8). However, complicating analysis is the clear difference between substrate KIE and solvent isotope effect; solvent isotope effects cannot be neatly interpreted in the context of solvent involvement in proton transfer at the transition state [78]. In systems where protodemetalation is rate determining, a metal-carbon bonded intermediate is allegedly characterized, as in the case of a related palladium-catalyzed hydroamination [47], and a propargylamide cyclization [44]. In our studies, the gold alkyl was not detected, even under these conditions where an isotope effect is observed or the solvent effect significantly slows the reaction. It is possible that incorporation of deuterated solvent is shifting the mechanism toward rate determining protodeauration, but this does not correspond to a buildup of alkylgold intermediates. Even at 10 and 20 mol % catalyst with urea **1a** (0.05 M) in CD_3_OD, where gold intermediates would be significantly more visible, no alkylgold was detected. In our previous report where alkylgold buildup is observed*,* we know a faster catalytic process is possible to form **3a** that is not consistent with a protodeauration pathway (see Scheme 1); so while deuterated methanol may be perturbing the rates of protodeauration, we propose such a pathway is not necessary to explain the results.

Small kinetic isotope effects can also arise when bond breaking is more or less than half complete at the transition state [79]. In foundational work on alkene hydration by Evans and Kirby, a general acid-catalyzed mechanism is concluded, and small isotope effects are observed despite H–O bond breaking in the transition state [80]. In an excellent study by Borhan and co-workers, it was found that in chlorination reactions, activation of alkenes is not driven by the electrophilicity of the reagent being attacked by an alkene*,* but instead depends on nucleophile assistance: the alkene becomes more nucleophilic upon interaction with the pendant attacking nucleophile [81]. In our earlier studies with basic gold reagents, the alkylgold intermediate is observed only in the presence of base further supporting the importance of nucleophile activation for gold π-activation. A mechanism that involves gold-carbonyl coordination via oxygen would serve to release of a proton in a way that nitrogen nucleophilicity is maintained, and in fact, enhanced. Ultimately, drawing analogy to non-gold-catalyzed reactions, the lack of observed primary KIE in the hydroamination of **1a**-*N*-d need not be explained by a fast (or any) protodeauration step.

## Conclusion

In summary, in efforts to reconcile the theoretical and experimental evidence that π-activation is challenging for alkenes with gold and the fact that there remains a lack of support for the kinetic viability of protodeauration in instances where gold alkyl is observed, we propose an alternate mechanism (Scheme 2) that may be better characterized as general acid catalysis (without invoking gold alkyl intermediates). Although many facets of this reaction remain to be understood, there are many practical implications of our work. For example, future catalyst optimization should focus on designing ligands that create more electrophilc gold without sacrificing stability, while future design of chiral ligands may depend on a better understanding of the chiral space generated when gold is coordinated to carbonyl functionality, rather than the alkene. Key observations include the following:

• Mixed solvent effects support the involvement of MeOH within the transition state; rate enhancements with protic co-solvents point to the importance of H-bonding clusters.

• Significant slow-down and primary isotope effects observed with MeOH versus MeOD; such KIE’s are typically used to support rate determining protodeauration, but in this case do not correspond to alkylgold buildup. In protic solvents perhaps the strength of the H-bonding network controls the rate of reaction [82].

• Stronger donor ligands that would enhance protodeauration do not increase the rate of reaction.

• Rates correlate most significantly with nucleophile effects; more basic carbonyls react faster and undergo N–H/D exchange faster in the presence of gold suggesting gold nucleophile interactions drive reactivity.

• Rate inhibition is observed at the highest concentrations of urea **1a** and amide **1b**; the increasing basicity would slow down any acid mediated processes.

• At low incorporation of deuterium, there appears to be a balance between the influence on rate from N–H/N–D exchange and any release of H/DOR which also participates in the reaction. Partially deuterium-labeled urea **1a** exhibits no primary kinetic isotope effect, however, trace amounts of protic water, which boosts reaction rates, may be counterbalancing any expected slowdown.

• Activation parameters suggest an ordered transition state. Reaction orders indicate an approach to zero-order dependence on gold catalyst when in the presence of MeOH, while first-order dependence on gold catalyst is maintained in DCM and MeOD.

Our experiments show that alkene hydroamination is accelerated by simple hydrogen bonding additives (water and alcohols), acceptor ligands (arylphosphines with or without electron-withdrawing substituents) and demonstrates a continuum of primary deuterium isotope effects from insignificant to significant. The reaction demonstrates features of being driven by both Lewis acidity of gold and proton transfer, instead of being localized to one of the two regimes. Furthermore, substituent effects (more reactive with more Lewis basic substrates) hint at gold carbonyl interactions being important to initiate reactivity [60,83]. It is becoming increasingly clear that homogeneous gold-catalyzed reactions can be influenced in complex and subtle ways by the reaction conditions, particularly, additive and counterion [48]. Factors that influence proton transfer will affect gold-mediated processes, regardless of whether gold alkyl intermediates are involved [44]. Continued understanding of how media impacts this particular reaction, and continued discussion of alternate gold-catalyzed mechanisms should help in the development of more efficient and powerful reaction methodologies.

## Supporting Information

File 1 Experimental procedures, kinetic data, and relevant spectra for kinetics reactions and intermediates.

File 2 Crystallographic experimental details.

File 3 X-ray crystallographic CIF file for **7a**.

## Data Availability

All data that supports the findings of this study is available in the published article and/or the supporting information to this article. Any additional data is available from the corresponding author upon reasonable request.

## References

[1] Teles J H, Brode S, Chabanas M (1998). Angew Chem, Int Ed.

[2] Kanno O, Kuriyama W, Wang Z J, Toste F D (2011). Angew Chem, Int Ed.

[3] Lee S D, Timmerman J C, Widenhoefer R A (2014). Adv Synth Catal.

[4] Zhang Z, Lee S D, Widenhoefer R A (2009). J Am Chem Soc.

[5] Michon C, Abadie M-A, Medina F, Agbossou-Niedercorn F (2017). J Organomet Chem.

[6] Abadie M-A, Trivelli X, Medina F, Capet F, Roussel P, Agbossou‐Niedercorn F, Michon C (2014). ChemCatChem.

[7] Abadie M-A, Trivelli X, Medina F, Duhal N, Kouach M, Linden B, Génin E, Vandewalle M, Capet F, Roussel P (2017). Chem – Eur J.

[8] Michon C, Abadie M-A, Medina F, Agbossou-Niedercorn F (2014). Catal Today.

[9] Kojima M, Mikami K (2012). Synlett.

[10] Dixit R, Sharma H, Agbossou-Niedercorn F, Vanka K, Michon C (2022). Catalysts.

[11] Timmerman J C, Laulhé S, Widenhoefer R A (2017). Org Lett.

[12] Han X, Widenhoefer R A (2006). Angew Chem, Int Ed.

[13] Bender C F, Widenhoefer R A (2006). Chem Commun.

[14] Bender C F, Widenhoefer R A (2006). Org Lett.

[15] Zhang J, Yang C-G, He C (2006). J Am Chem Soc.

[16] Brouwer C, He C (2006). Angew Chem, Int Ed.

[17] Serrano‐Becerra J M, Maier A F G, González‐Gallardo S, Moos E, Kaub C, Gaffga M, Niedner‐Schatteburg G, Roesky P W, Breher F, Paradies J (2014). Eur J Org Chem.

[18] Xiao Y-P, Liu X-Y, Che C-M (2011). Angew Chem, Int Ed.

[19] Timmerman J C, Schmitt W W, Widenhoefer R A (2016). Org Lett.

[20] Kumar M, Jasinski J, Hammond G B, Xu B (2014). Chem – Eur J.

[21] Hu X, Martin D, Melaimi M, Bertrand G (2014). J Am Chem Soc.

[22] Navarro M, Alférez M G, de Sousa M, Miranda-Pizarro J, Campos J (2022). ACS Catal.

[23] Guérinot A, Fang W, Sircoglou M, Bour C, Bezzenine‐Lafollée S, Gandon V (2013). Angew Chem, Int Ed.

[24] Chen Y, Yan W, Akhmedov N G, Shi X (2010). Org Lett.

[25] Bartolomé C, Ramiro Z, Peñas-Defrutos M N, Espinet P (2016). ACS Catal.

[26] Rosenfeld D C, Shekhar S, Takemiya A, Utsunomiya M, Hartwig J F (2006). Org Lett.

[27] LaLonde R L, Brenzovich W E, Benitez D, Tkatchouk E, Kelley K, Goddard W A, Toste F D (2010). Chem Sci.

[28] Zhu Y, Zhou W, Petryna E M, Rogers B R, Day C S, Jones A C (2016). ACS Catal.

[29] Roth K E, Blum S A (2010). Organometallics.

[30] Gubler J, Radić M, Stöferle Y, Chen P (2022). Chem – Eur J.

[31] McKinney Brooner R E, Widenhoefer R A (2011). Chem – Eur J.

[32] Taylor J G, Adrio L A, Hii K K (Mimi) (2010). Dalton Trans.

[33] Li Z, Zhang J, Brouwer C, Yang C-G, Reich N W, He C (2006). Org Lett.

[34] Asgari M, Hyland C J T, Hashmi A S K, Yates B F, Ariafard A (2019). Catal Sci Technol.

[35] Mehrabi T, Ariafard A (2016). Chem Commun.

[36] Griebel C, Hodges D D, Yager B R, Liu F L, Zhou W, Makaravage K J, Zhu Y, Norman S G, Lan R, Day C S (2020). Organometallics.

[37] Malhotra D, Mashuta M S, Hammond G B, Xu B (2014). Angew Chem, Int Ed.

[38] Tolman C A (1977). Chem Rev.

[39] Stein P M, Rudolph M, Hashmi A S K (2021). Adv Synth Catal.

[40] Wang W, Hammond G B, Xu B (2012). J Am Chem Soc.

[41] Corma A, Ruiz V R, Leyva‐Pérez A, Sabater M J (2010). Adv Synth Catal.

[42] Tárkányi G, Király P, Pálinkás G, Deák A (2007). Magn Reson Chem.

[R43] 43CCDC Deposition Number 2215205; Summary of Data; Data Block Name: data_b13a5; Unit Cell Parameters: *a* 13.9857(9), *b* 10.9855(7), *c* 15.0464(10) Pn.

[44] Wang W, Kumar M, Hammond G B, Xu B (2014). Org Lett.

[45] Bárta O, Císařová I, Schulz J, Štěpnička P (2019). New J Chem.

[46] Bender C F, Brown T J, Widenhoefer R A (2016). Organometallics.

[47] Cochran B M, Michael F E (2008). J Am Chem Soc.

[48] Lu Z, Li T, Mudshinge S R, Xu B, Hammond G B (2021). Chem Rev.

[49] Sciortino G, Muñoz-López S, Lledós A, Ujaque G (2020). Organometallics.

[50] Wang F, Ma S, Zhang D, Cooks R G (1998). J Phys Chem A.

[51] Wilden J D (2010). J Chem Res.

[52] Reddick J J, Cheng J, Roush W R (2003). Org Lett.

[53] (2022). Reich Collection, Bordwell pKa Table.

[54] Sladojevich F, Fuentes de Arriba Á L, Ortín I, Yang T, Ferrali A, Paton R S, Dixon D J (2013). Chem – Eur J.

[55] Takagi R, Duong D T, Ichiki T (2021). Tetrahedron.

[56] Yu Z-L, Cheng Y-F, Jiang N-C, Wang J, Fan L-W, Yuan Y, Li Z-L, Gu Q-S, Liu X-Y (2020). Chem Sci.

[57] Lin J-S, Yu P, Huang L, Zhang P, Tan B, Liu X-Y (2015). Angew Chem, Int Ed.

[R58] 58Analogous carbamates show no reaction with HOTf in MeOH at 50 °C [6] and analogous ureas show no reaction with HOTf in MeOH at room temperature [14].

[59] Wang N, Fan L-W, Zhang J, Gu Q-S, Lin J-S, Chen G-Q, Liu X-Y, Yu P (2022). Org Chem Front.

[60] Asao N, Asano T, Yamamoto Y (2001). Angew Chem, Int Ed.

[61] BabaAhmadi R, Ghanbari P, Rajabi N A, Hashmi A S K, Yates B F, Ariafard A (2015). Organometallics.

[62] Zhu Y, Yu B (2011). Angew Chem, Int Ed.

[63] Pozhydaiev V, Power M, Gandon V, Moran J, Lebœuf D (2020). Chem Commun.

[64] Tzouras N V, Gobbo A, Pozsoni N B, Chalkidis S G, Bhandary S, Van Hecke K, Vougioukalakis G C, Nolan S P (2022). Chem Commun.

[65] Li W, Shi R, Chen S, Zhang X, Peng W, Chen S, Li J, Xu X-M, Zhu Y-P, Wang X (2022). J Org Chem.

[66] Zhdanko A, Ströbele M, Maier M E (2012). Chem – Eur J.

[67] Cheon C H, Kanno O, Toste F D (2011). J Am Chem Soc.

[68] Marion N, Carlqvist P, Gealageas R, de Frémont P, Maseras F, Nolan S P (2007). Chem – Eur J.

[69] Brooner R E M, Widenhoefer R A (2013). Angew Chem, Int Ed.

[70] Epton R G, Unsworth W P, Lynam J M (2022). Organometallics.

[71] Harris R J, Carden R G, Duncan A N, Widenhoefer R A (2018). ACS Catal.

[72] Harris R J, Nakafuku K, Duncan A N, Carden R G, Timmerman J C, Widenhoefer R A (2021). Chem – Eur J.

[73] Brown T J, Weber D, Gagné M R, Widenhoefer R A (2012). J Am Chem Soc.

[74] Weber S G, Zahner D, Rominger F, Straub B F (2013). ChemCatChem.

[75] Truong P T, Miller S G, McLaughlin Sta. Maria E J, Bowring M A (2021). Chem – Eur J.

[76] Koszinowski K, Stephenson D S (2018). J Org Chem.

[77] Anslyn E V, Dougherty D A (2004). Experiments Related to Thermodynamics and Kinetics. Modern Physical Organic Chemistry.

[78] D’Souza M J, Kevill D N (2022). Beilstein J Org Chem.

[79] Carey F A, Sundberg R J (2007). Structural Effects on Stability and Reactivity. Advanced Organic Chemistry Part A: Structure and Mechanisms.

[80] Evans C M, Kirby A J (1984). J Chem Soc, Perkin Trans 2.

[81] Ashtekar K D, Vetticatt M, Yousefi R, Jackson J E, Borhan B (2016). J Am Chem Soc.

[82] Scheiner S, Čuma M (1996). J Am Chem Soc.

[83] Kovács G, Ujaque G, Lledós A (2008). J Am Chem Soc.

