# Supplementary material for: Ligand effects, solvent cooperation, and large kinetic solvent deuterium isotope effects in gold(I)-catalyzed intramolecular alkene hydroamination
Source: Beilstein J Org Chem. 2024 Feb 29;20:479–96. doi: 10.3762/bjoc.20.43 (PMC10910400; doi:10.3762/bjoc.20.43)
Supplement: File 1 — Experimental procedures, kinetic data, and relevant spectra for kinetics reactions and intermediates. [file Beilstein_J_Org_Chem-20-479-s001.pdf]

BEILSTEIN JOURNAL OF ORGANIC CHEMISTRY

Supporting Information

for

Ligand effects, solvent cooperation, and large kinetic solvent deuterium isotope effects in gold(I)-catalyzed intramolecular alkene hydroamination

Ruichen Lan, Brock Yager, Yoonsun Jee, Cynthia S. Day and Amanda C. Jones

Beilstein J. Org. Chem. **2024,** *20,* 479–496. doi:10.3762/bjoc.20.43

Experimental procedures, kinetic data, and relevant spectra for kinetics reactions and intermediates

License and Terms: This is a supporting information file under the terms of the Creative Commons Attribution License (https://creativecommons.org/licenses/by/4.0). Please note that the reuse, redistribution and reproduction in particular requires that the author(s) and source are credited and that individual graphics may be subject to special legal provisions.
The license is subject to the *Beilstein Journal of Organic Chemistry* terms and conditions: (https://www.beilstein-journals.org/bjoc/terms)

Table of Contents

General Experimental S3
Ligand Effect with Urea (Table 1 in Paper) S3
Solvent Effect (Table 2 in Paper) S6
MeOH-d4 .... S6
Acetonitrile S7
THF-D8 .... S7
DCM-d2 with 10% CD3OD (v/v) S8
DCM-d2 with 10% CH3OH (v/v) S9
CH_2Cl_2 .... S9
CD_2Cl_2 .... S10
CH_3OH S11
JackiphosAuNTf$_2$ in MeOH versus CD_2Cl_2 .... S12
CD_2Cl_2 .... S12
CH_3OH S12
Rate with Mixed DCM/Methanol and [JPhosAu(NCCH3)]SbF6 (Figure 2 in Paper) S13
Variable Impact of Additive Depending on Catalyst and Solvent (Table 3/Figure 3 In paper) S14
JPhosAu(NCCH3)SbF6 in CD_2Cl_2 Rate boosted than plateaus with Water S14
JPhosAuOTf in CD_2Cl_2 Enhanced by Water S15
JPhosAuOTf in MeOH Diminished by Water S16
JPhosAu($NCCH_3$)SbF_6 in MeOH Diminished by Water S17
Reactions with Various Substrates (Table 4 in Paper) S18
Reaction with Sulfonamide (**1d**) and [JphosAu($NCCH_3$)]SbF_6 .... S18
Reaction with Carbamate (**1b**) and [JphosAu($NCCH_3$)]SbF_6 .... S18
Reactions with Benzamide (**1c**)(Table 5 in Paper) S19
Examining Decomposition in Reaction with carbamate (**1b**) and Catalysts **4c** and **4a** S21
Bisphos Syntheses, ^{31}P Chemical Shifts for Decomposition Byproducts S23
Reactions with tris(2,4-di-tert-butylphenyl)phosphite ligand. S25
Kinetic Isotope Effect Measurements (Table 7 and 8 In paper) S27
Urea with Titrated CH_3OD (Compare to Figure S13 for KIE) S27
Preparation of Partially Deuterated Urea and Rate S28
Reaction of Partially Deuterated Urea with [JphosAu($NCCH_3$)]SbF_6 .... S28

Carbamate with Titrated CD_3OD (Compare to Figure S18 for KIE) S29
Solvent Deuterium Isotope Effects (Carbamate/Johnphos) S31
Solvent Deuterium Isotope Effects (Urea/Jackiephos) S32
Other Additives (Figure 6 in Paper) S33
Order in Catalyst (Figure 8 in Paper) S34
Order in Catalyst in CD_3OD S37
Order in Urea S37
Order in Carbamate (Figure 7 in Paper) S39
Activation Parameters S40
References S42

General Experimental

Unless otherwise noted commercial materials were used without further purification. Complexes were prepared in an oxygen-free, moisture-free glovebox or under Argon using Schlenk techniques. Deuterated solvents were purchased from Cambridge Isotope Laboratories. Unless otherwise stated, the deuterated solvents for kinetics experiments were used as received. All other solvents were purchased anhydrous. NMR spectra were obtained on Bruker spectrometers operating at a proton frequency of either 300, 400 or 500 MHz. Unless otherwise noted, spectra were collected at 25 °C. For kinetic plots, the total concentrations of alkene starting material and cyclized product were normalized, assuming that the sum of the signals for each were constant.

Observed rates and corresponding errors for individual experiments were calculated using the LINEST function in EXCEL. Where multiple trials were conducted, standard deviation on the average was calculated.

Ligand Effect with Urea (Table 1 in Paper)

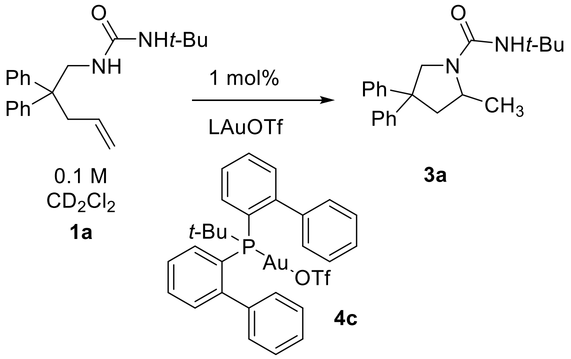

The urea starting material was prepared according to literature procedure[1] and the cyclized product was characterized by ^{1}H NMR spectroscopy in situ.

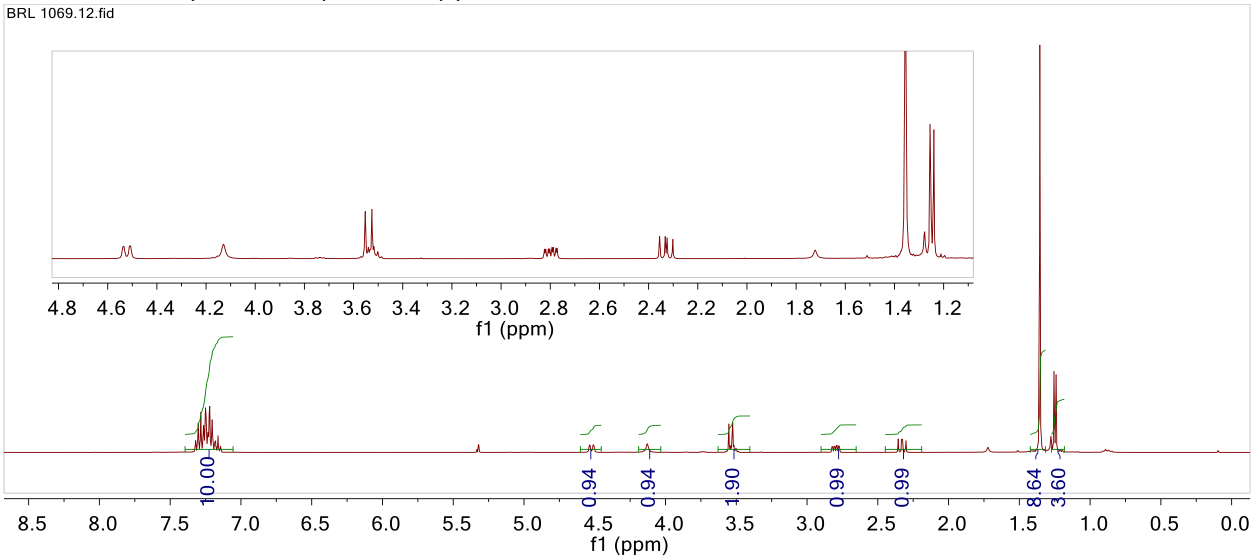

^{1}H NMR Spectrum of 3a: (CD_2Cl_2, 400.17 MHz): 7.33−7.11 (m, 10H), 4.52 (dd, J=10.8, 2.1 Hz, 1H), 4.13 (s, 1H), 3.54 (d, J=10.8 Hz, 1H), 3.51 (m, 1H), 2.80 (ddd, J=12.3, 6.4, 2.2 Hz, 1H), 2.33 (dd, J=12.3, 9.2 Hz, 1H), 1.36 (s, 9H), 1.25 (d, J=6.1 Hz, 3H).

A stock solution of catalyst (0.0524 M) was prepared by dissolving **4c** (740.53 g/mol, 77.6 mg, 0.10 mmol) in 1.94 mL of CD_2Cl_2. Urea alkene **1a** (336.48 g/mol, 24 mg, 0.071 mmol) was dissolved 0.687 mL of CD_2Cl_2 and transferred to an NMR tube. Catalyst solution (13 µL, 0.0007 mmol) was injected to initiate the reaction and to bring the total volume to 0.7 mL (the substrate was at a concentration of 0.1 M); the sample was transferred to the NMR spectrometer and ^{1}H NMR spectra collected. The time from injection to spectrum #1 was approximately 5 minutes. Pseudo first order plots were linear up to at least ≈85% conversion.

	CD_2Cl_2	Catalyst Solution	Catalyst Amount	Catalyst Loading	$10^5 \times K_{obs}/s^{-1}$	
BRL 1065	0.687 mL	0.013 mL	0.0007 mmol	1 mol %	28 ± 1	average: 58 ± 27
BRL 1067	0.687 mL	0.013 mL	0.0007 mmol	1 mol %	79 ± 6	
BRL 1069	0.687 mL	0.013 mL	0.0007 mmol	1 mol %	67 ± 7	

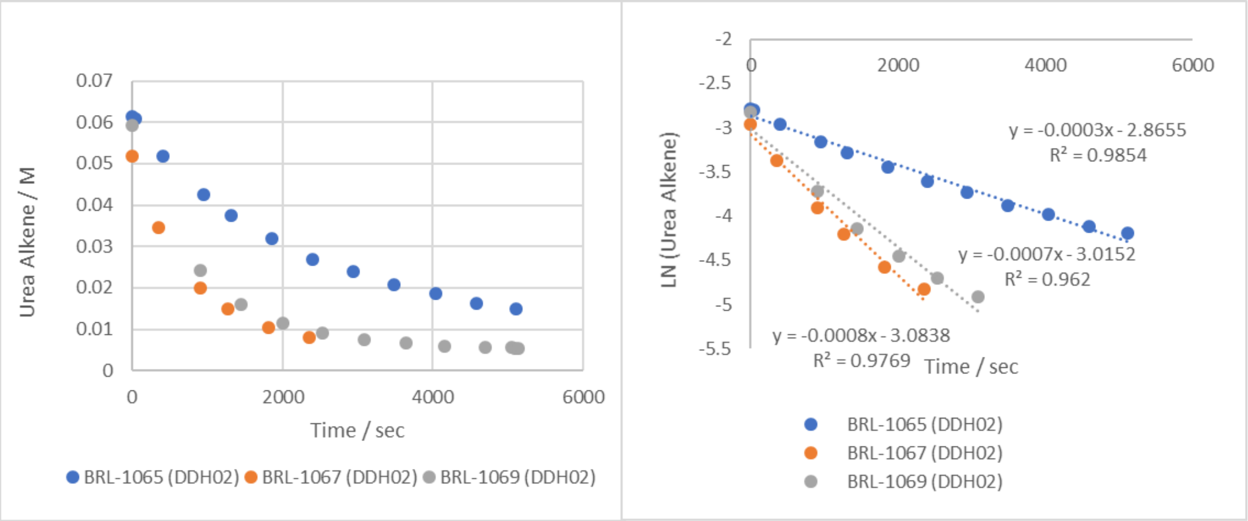

Figure S1. Raw plots of urea alkene decay (0.1 M) in 0.7 mL of CD_2Cl_2 with 0.0007 mmol **4c** (1 mol %) and corresponding first order plots.

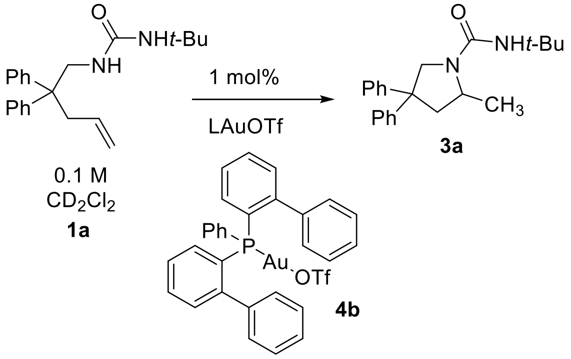

A stock solution of catalyst (0.054 M) was prepared by dissolving **4b** (760.53 g/mol, 120.6 mg, 0.159 mmol) in 2.94 mL of CD_2Cl_2. Urea alkene **1a** (336.48 g/mol, 24 mg, 0.071 mmol) was dissolved 0.687 mL of CD_2Cl_2 and transferred to an NMR tube. Catalyst solution (13 µL, 0.0007 mmol) was injected to initiate the

reaction and to bring the total volume to 0.7 mL (the substrate was at a concentration of 0.1 M); the sample was transferred to the NMR spectrometer and ^{1}H NMR spectra collected. The time from injection to spectrum #1 was approximately 5 minutes. Pseudo first order plots were linear up to at least ≈94% conversion.

	CD_2Cl_2	Catalyst Solution	Catalyst Amount	Catalyst Loading	$10^5 \times k_{obs}/s^{-1}$	
BRL 1081	0.687 mL	0.013 mL	0.0007 mmol	1 mol %	93 ± 7	Average 106 ± 13
BRL 1083	0.687 mL	0.013 mL	0.0007 mmol	1 mol %	107 ± 6	
BRL 1085	0.687 mL	0.013 mL	0.0007 mmol	1 mol %	119 ± 9	

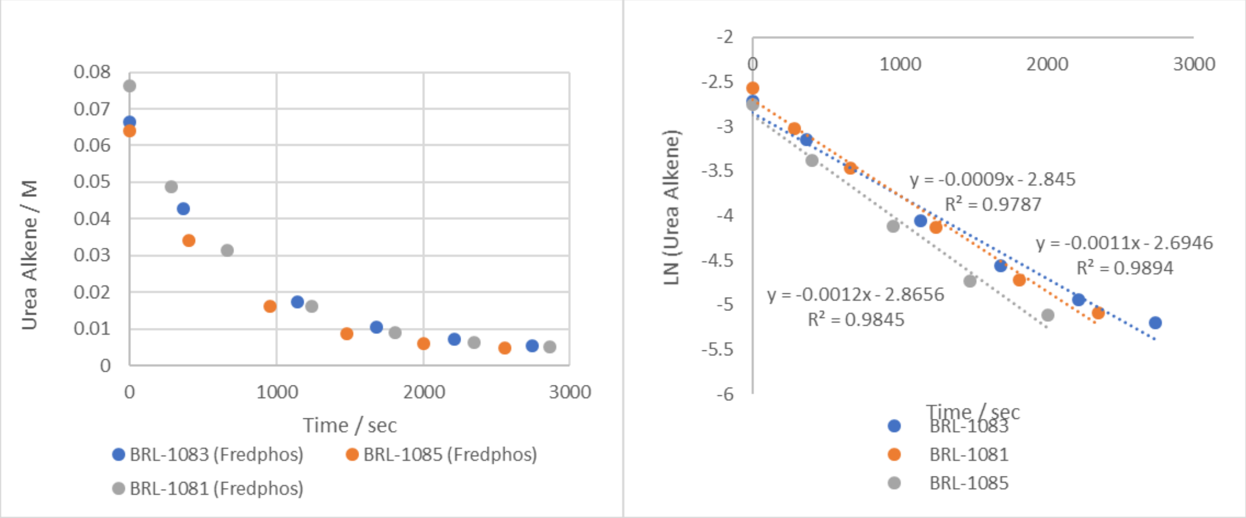

Figure S2. Raw plots of urea alkene **1a** decay (0.1 M) in **0.7 mL** of CD_2Cl_2 with 0.0007 mmol of **4b** (1 mol %) and corresponding first order plots.

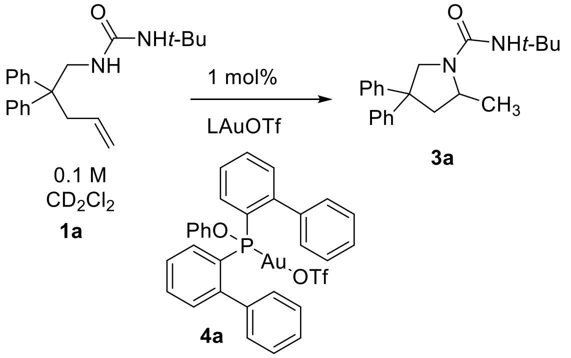

A stock solution of catalyst (0.028M) was prepared by dissolving **4a** (776.52 g/mol, 85.3 mg, 0.11 mmol) in 3.95 mL of CD_2Cl_2. Urea alkene **1a** (336.48 g/mol, 25 mg, 0.074 mmol) was dissolved 0.675 mL of CD_2Cl_2 and transferred to an NMR tube. Catalyst solution (25 µL, 0.0007 mmol) was injected to initiate the reaction and to bring the total volume to 0.7mL (the substrate was at a concentration of 0.1 M); the sample was transferred to the NMR spectrometer and ^{1}H NMR spectra collected. The time from injection to spectrum #1 was approximately 5 minutes. Pseudo first order plots were linear up to at least ≈92% conversion.

	CD_2Cl_2	Catalyst Solution	Catalyst Amount	Catalyst Loading	$10^5 \times k_{obs}/s^{-1}$	
BRL 1031	0.675 mL	0.025 mL	0.0007 mmol	1 mol %	192 ± 10	

BRL 1035	0.675 mL	0.025 mL	0.0007 mmol	1 mol %	178 ± 18	average:
BRL 1037	0.675 mL	0.025 mL	0.0007 mmol	1 mol %	247 ± 12	206 ± 37

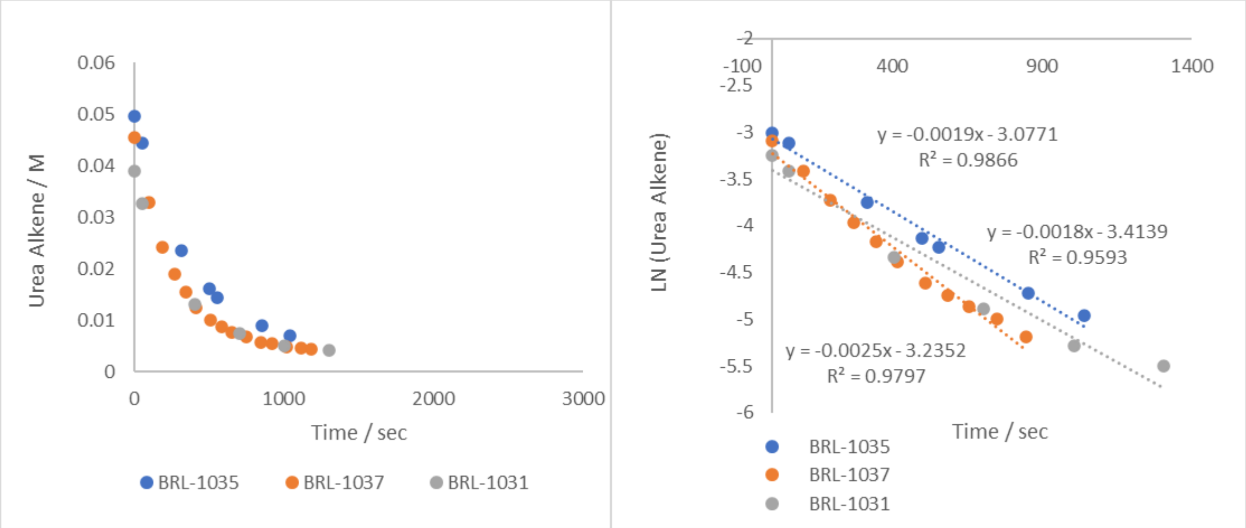

Figure S3. Raw plots of urea alkene decay **1a** (0.1 M) in 0.7 mL of CD_2Cl_2 with 0.0007 mmol of **4c** (1 mol %) and corresponding first order plots.

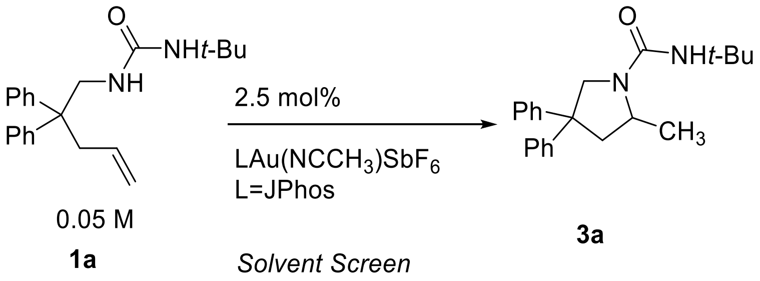

Solvent Effect (Table 2 in Paper)

A representative procedure is as follows. A stock solution of catalyst (0.036 M) was prepared by dissolving JPhosAu($NCCH_3$)SbF_6 (772.17 g/mol, 68.9 mg, 0.0892 mmol) in 2.5 mL of CH_2Cl_2. Urea alkene **1a** (336.48 g/mol, 12 mg, 0.036 mmol) was dissolved in 0.675 mL of CH_2Cl_2 and transferred to an NMR tube. The catalyst solution (25 µL, 0.000892 mmol) was injected to initiate the reaction and to bring the total volume to 0.7 mL (the substrate was at a concentration of 0.05 M). The sample was transferred to the NMR spectrometer and ^1H NMR spectra collected. Similar procedures were followed with variation of the reaction solvent.

MeOH-d4

	$10^5 \times k_{obs}/s^{-1}$
BRR-2039	3.68 ± 0.02
BRL-4031	4.42 ± 0.03
BRL-4033	4.24 ± 0.02

BRL-4035	4.42 ± 0.06
Average	4.2 ± 0.3

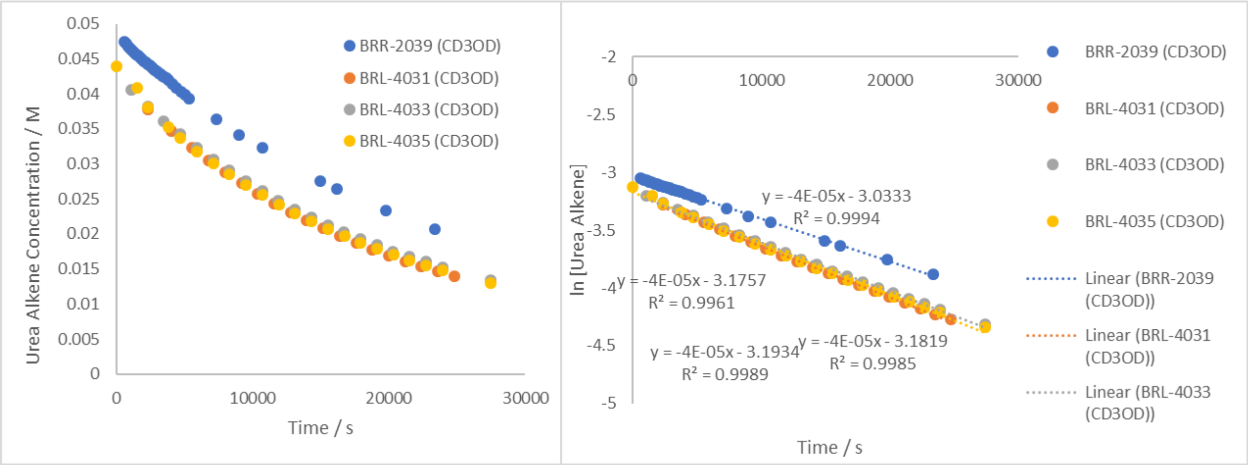

Figure S4. Raw plots of urea alkene decay (0.05 M) in 0.7 mL of solvent with JPhosAu($NCCH_3$)SbF_6 (2.5 mol %) and corresponding first order plots.

Acetonitrile

	$10^5 \times k_{obs}/s^{-1}$
BRR-2033	1.75 ± 0.01

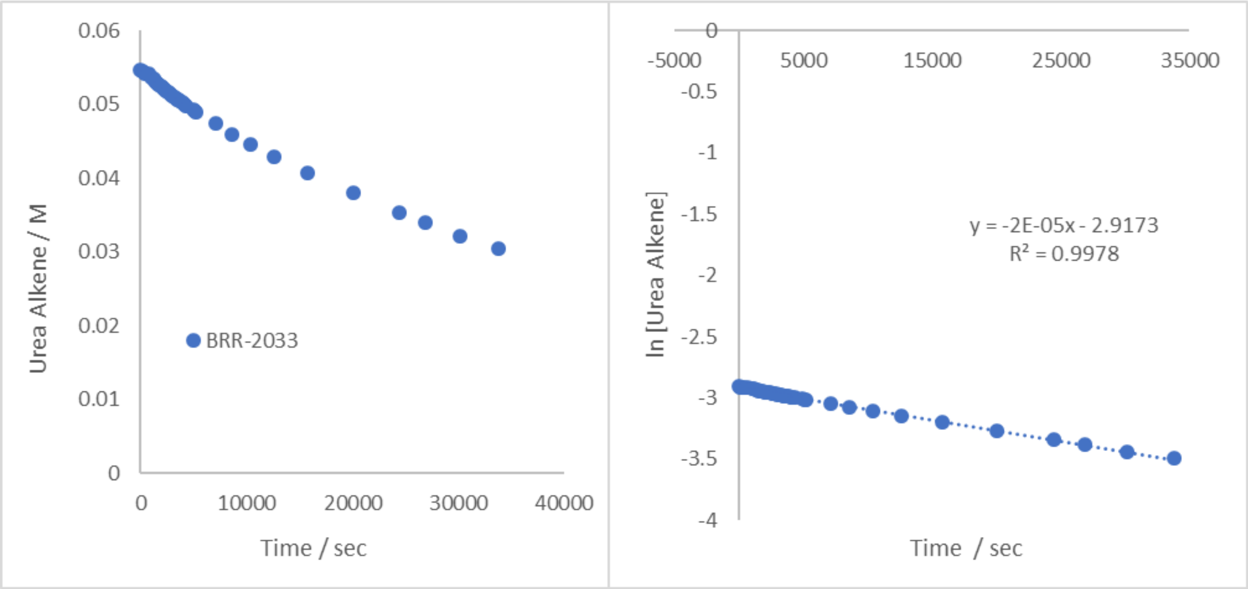

Figure S5. Raw plots of urea alkene decay (0.05 M) in 0.7 mL of solvent with JPhosAu($NCCH_3$)SbF_6 (2.5 mol %) and corresponding first order plots.

THF-D8

	$10^5 \times k_{obs}/s^{-1}$
BRL-6021	90 ± 3

BRL-6023	41.9 ± 0.9
BRL-6025	105 ± 4
BRL-6033	95 ± 4
BRL-6035	105 ± 5
Average	87 ± 26

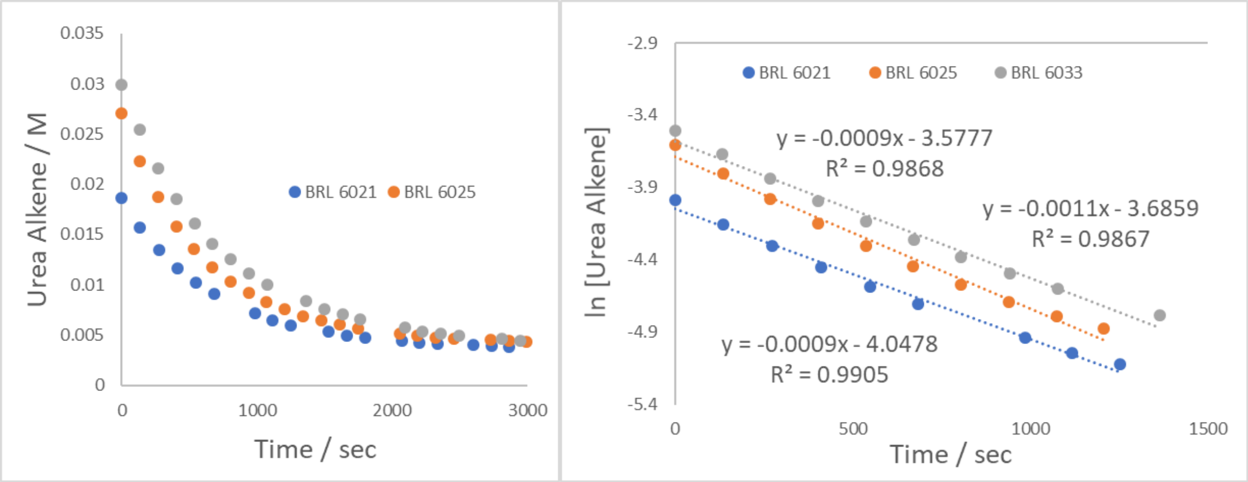

Figure S6. Raw plots of urea alkene decay (0.05 M) in 0.7 mL of THF-d8 with JPhosAu($NCCH_3$)SbF_6 (2.5 mol %) and corresponding first order plots.

DCM-d2 with 10% CD3OD (v/v)

	$10^5 \times k_{obs}/s^{-1}$
BRR-6117	166 ± 13
BRR-6119	133 ± 2
Average	150 ± 23

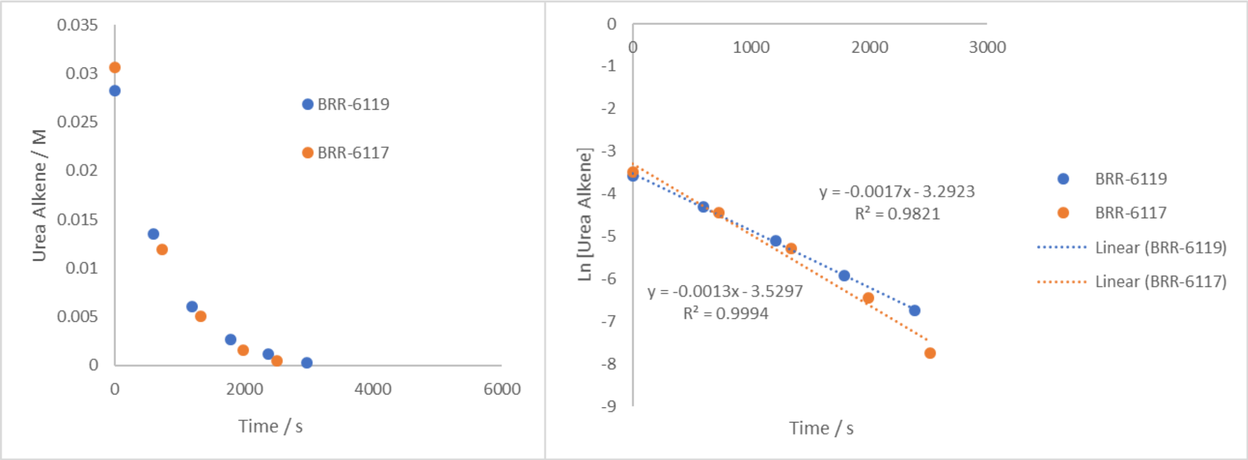

Figure S7. Raw plots of urea alkene decay (0.05 M) in 0.7 mL solvent (540 μL CD_2Cl_2 with 60 μL CD_3OD) withJPhosAu($NCCH_3$)SbF_6 (2.5 mol %) and corresponding first order plots.

DCM-d2 with 10% CH3OH (v/v)

This procedure utilized 60 μL CH_3OH with 540 μL CD_2Cl_2. A rate constant of 0.008 s^{-1} was estimated based on the observed 90% conversion by the collection of the 1st NMR spectrum obtained (time ca 300 seconds).

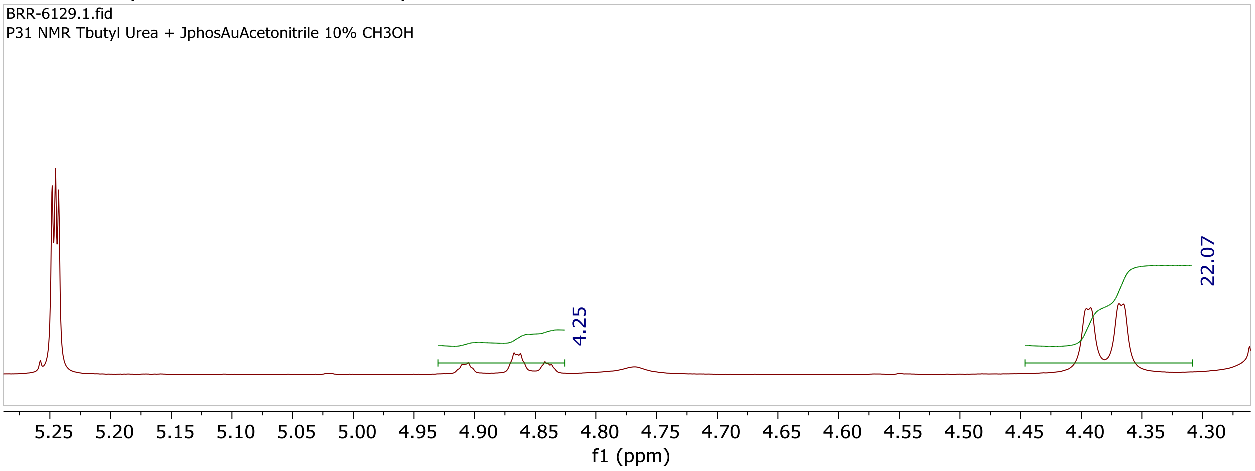

Figure S8. ^{1}H NMR spectrum collected within 5 minutes of catalyst addition, for reaction of **1a** with [JPhosAu($NCCH_3$)]SbF_6 in CD_2Cl_2 with 10% CH_3OH (v/v).

CH_2Cl_2

First order plots were linear up to ≈66% conversion.

	$10^5 \times k_{obs}/s^{-1}$
BRL-2033	51 ± 2
BRL-2035	52 ± 2
BRL-2037	50 ± 2
Average	51 ± 1

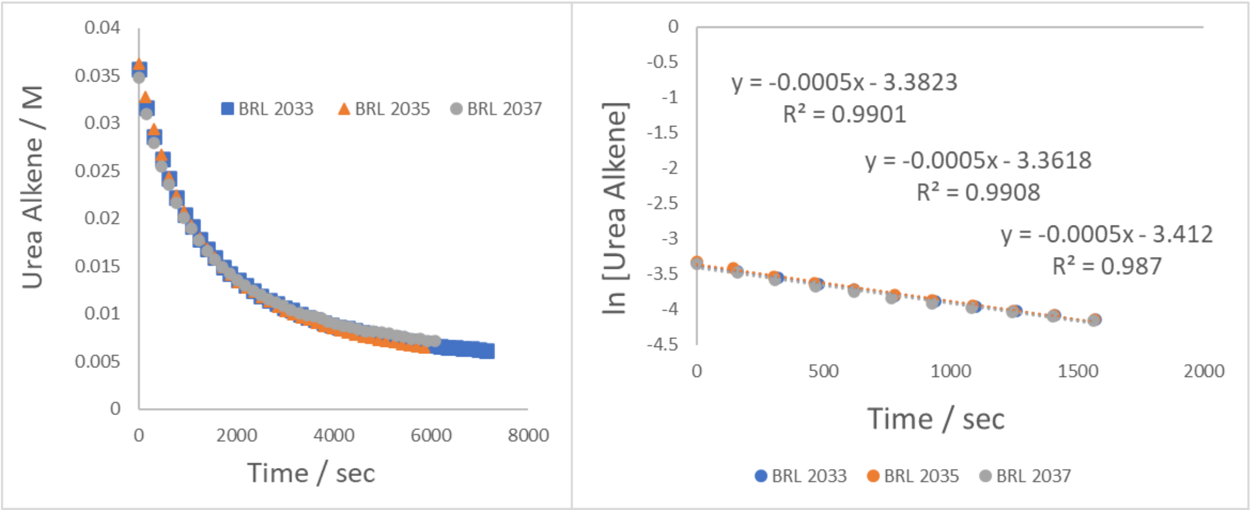

Figure S9. Raw plots of urea alkene decay (0.05 M) in 0.7 mL of CH_2Cl_2 with 0.0009 mmol of [JPhosAu($NCCH_3$)]SbF_6 (2.5 mol %) and corresponding first order plots.

CD_2Cl_2

First order plots were linear up to 70% conversion.

	$10^5 \times k_{obs}/s^{-1}$
BRL-4139	35.9 ± 0.8
BRL-4141	35.8 ± 0.9
BRL-4143	38 ± 1
Average	37 ± 1

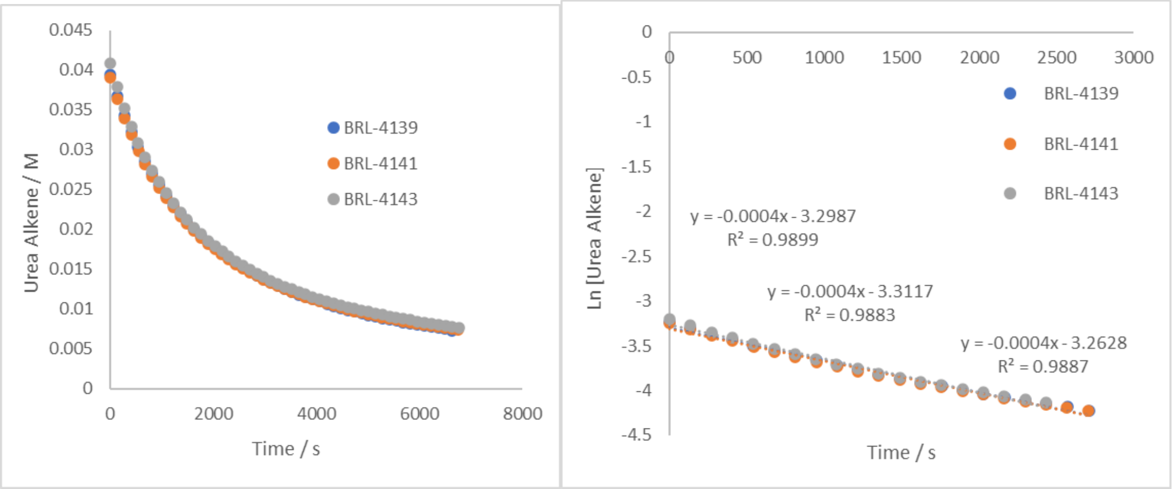

Figure S10. Raw and first order plots of urea alkene decay (0.05 M) in 0.7 mL of CD_2Cl_2 with [JPhosAu($CNCH_3$)]SbF_6 (2.5 mol %).

CH_3OH

Negligible differences in rate were seen when using JPhosAuOTf versus [JPhosAu($NCCH_3$)]SbF6. Plots were linear up to ≥80% conversion.

	**Catalyst Solution**	**Catalyst Amount**	**Catalyst Loading**	**$10^5 \times k_{obs}/s^{-1}$**	
BRL-2039*	50 µL	0.00089 mmol	2.5mol %	50 ± 1	Average 50 ± 7
BRL-1127	25 µL	0.00089 mmol	2.5 mol %	46 ± 1	
BRL-1129	25 µL	0.00089 mmol	2.5 mol %	61.5 ± 0.7	
BRL-1131	25 µL	0.00089 mmol	2.5 mol %	45 ± 1	

*With [JPhosAu($NCCH_3$)]SbF_6.

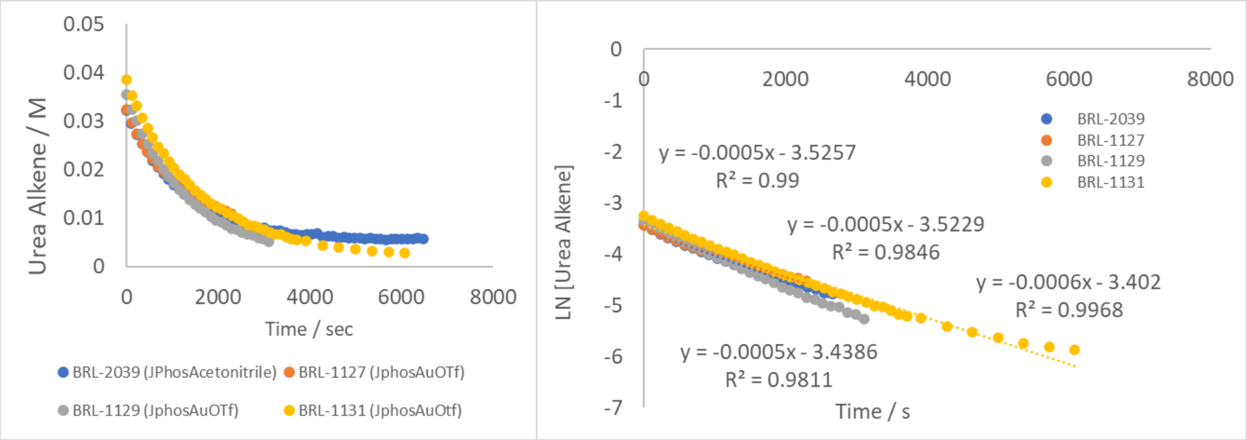

Figure S11. Raw plots of urea alkene decay (0.05 M) in 0.7 mL of CH_3OH with 0.00089 mmol of [JPhosAu($CNCH_3$)]SbF_6 (2.5 mol %) or JPhosAuOTf and corresponding first order plots

JackiphosAuNTf$_2$ in MeOH versus CD$_2$Cl$_2$

When JackiephosAuNTf$_2$ was used, rates were not the same in DCM and MeOH. Also reactions of **1a** with JackiephosAuNTf$_2$ are slightly slower than with JphosAuOTf when in pure MeOH.

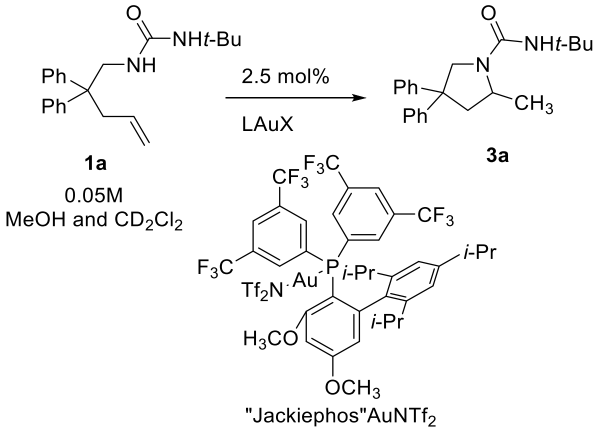

CD$_2$Cl$_2$

A stock solution of catalyst (0.016 M) was prepared by dissolving Jackiephos-Au-(NTf)$_2$ (1273.78 g/mol, 28.4 mg, 0.022 mmol) in 1.38 mL of CD$_2$Cl$_2$. Urea alkene (336.48 g/mol, 12 mg, 0.0356 mmol) was dissolved in 0.645 mL (645µL) of CD$_2$Cl$_2$ and transferred to an NMR tube. Catalyst solution (55µL, 0.00089 mmol) was injected to initiate the reaction and to bring the total volume to 0.7 mL (the substrate was at a concentration of 0.05 M); the sample was transferred to the NMR spectrometer and ^{1}H NMR spectra collected. The time from injection to spectrum #1 was approximately 5 minutes. However, these two reactions afforded the product in full conversion too fast for the NMR spectrometer to detect any changes. If we assume 95% conversion at 5 minutes this corresponds to a k_{obs} = 0.01 s^{-1}.

CH$_3$OH

A stock solution of catalyst (0.0178 M) was prepared by dissolving JackiephosAu(NTf$_2$) (1273.78 g/mol, 114 mg, 0.089 mmol) in 5 mL of CH$_3$OH. Urea alkene (336.48 g/mol, 12 mg, 0.0356 mmol) was dissolved in 0.650 mL (650 µL) of CH$_3$OH and transferred to an NMR tube. Catalyst solution (50 µL, 0.00089 mmol) was injected to initiate the reaction and to bring the total volume to 0.7 mL (the substrate was at a concentration of 0.05 M); the sample was transferred to the NMR spectrometer and ^{1}H NMR spectra collected. The time from injection to spectrum #1 was approximately 5 minutes. Pseudo first order plots were linear up to at least 66% conversion.

	**CH$_3$OH**	**Catalyst Solution**	**Catalyst Amount**	**Catalyst Loading**	**$10^5 \times k_{obs}$/s^{-1}**	
BRL 4057	0.65 mL	0.05 mL	0.00089 mmol	2.5 mol %	26.2 ± 0.6	Average 23 ± 5
BRL 4059	0.65 mL	0.05 mL	0.00089 mmol	2.5 mol %	16.7 ± 0.2	
BRL 4061	0.65 mL	0.05 mL	0.00089 mmol	2.5 mol %	25.9 ± 0.6	

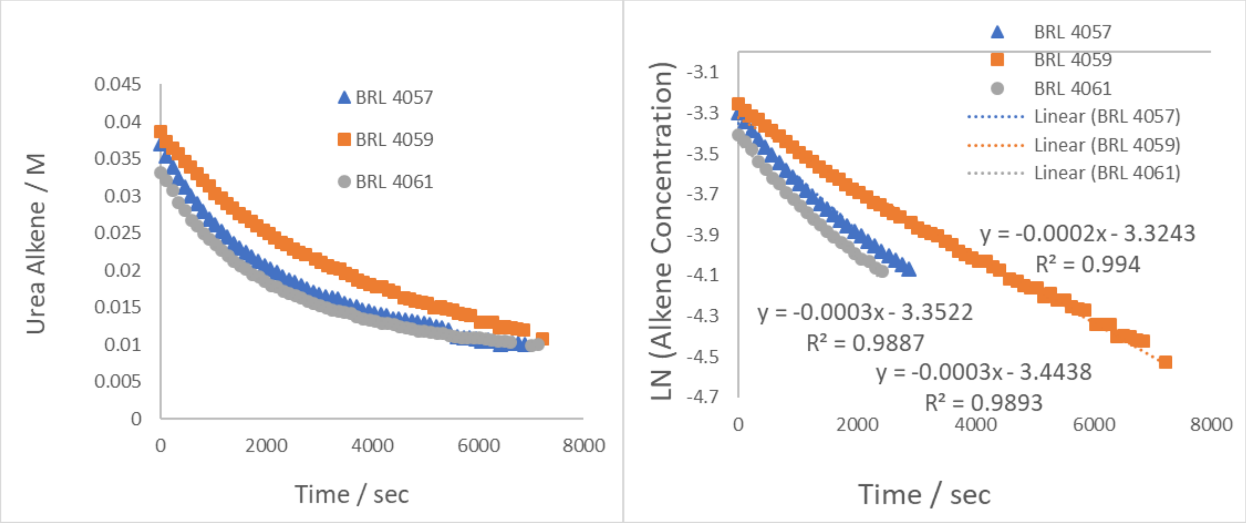

Figure S12. Raw and first order plots of urea alkene (0.05 M) decay in 0.7 mL of CH_3OH with 0.00089 mmol of JackiephosAuNTf$_2$ (2.5 mol %).

Rate with Mixed DCM/Methanol and [JPhosAu(NCCH3)]SbF6 (Figure 2 in Paper)

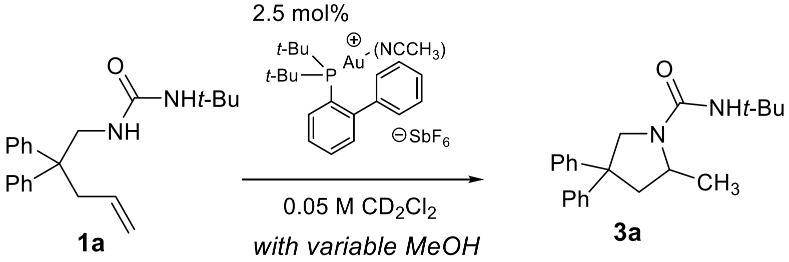

Procedure in CD_2Cl_2 with variable amounts of MeOH. A stock solution of catalyst (0.036 M) was prepared by dissolving JPhosAu(NCCH$_3$)SbF$_6$ (772.17 g/mol, 68.9 mg, 0.0892 mmol) in 2.5 mL of CD_2Cl_2. Urea Alkene (336.48 g/mol, 12 mg, 0.036 mmol) was dissolved in 0.67 mL, 0.65 mL or 0.62 mL of CD_2Cl_2 with variable amount of methanol and then the solution was transferred to an NMR tube. Catalyst solution (25 µL, 0.000892 mmol) was injected to initiate the reaction after bringing the total volume to 0.7 mL (substrate was at a concentration of 0.05 M). The sample was immediately transferred to the NMR spectrometer and ^{1}H NMR spectra collected. The time from injection to spectrum #1 was approximately 5 minutes. Pseudo first order plots were linear up to at least 84%.

	Methanol	Catalyst Solution	Catalyst Amount	Catalyst Loading	$10^5 \times k_{obs}/s^{-1}$
BRL 2019	5 µL	0.025 mL	0.0009 mmol	2.5 mol %	97 ± 3
BRL 2021	25 µL	0.025 mL	0.0009 mmol	2.5 mol %	381 ± 6
BRL 2023	55 µL	0.025 mL	0.0009 mmol	2.5 mol %	539 ± 30

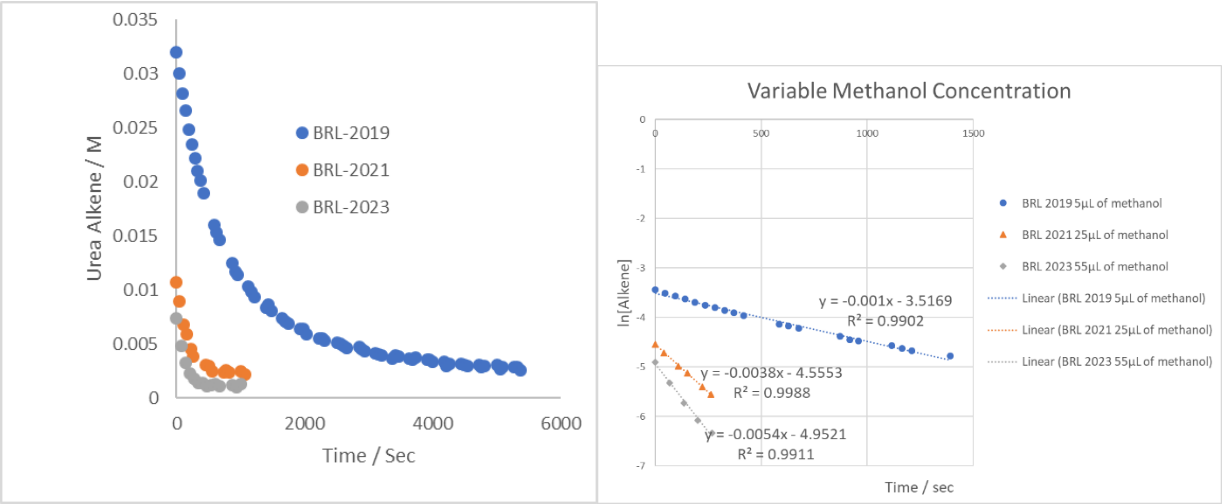

Figure S13. Raw plots of urea alkene decay (0.05 M) in variable amount of CD_2Cl_2 and CH_3OH (total volume 0.7 mL) with 0.00089 mmol of [JPhosAu($NCCH_3$)]SbF_6 (2.5 mol %) , and the corresponding first order plots.

Variable Impact of Additive Depending on Catalyst and Solvent (Table 3/Figure 3 In paper)

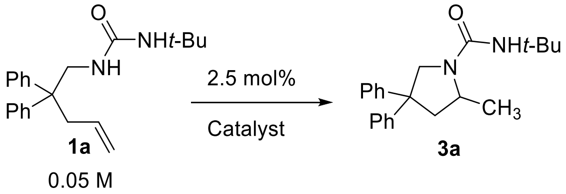

JPhosAu($NCCH_3$)SbF_6 (**5**) in DCM-d_2, *Rate boosted than plateaus with water additive.*

JPhosAu(NCCH3)SbF6 in CD_2Cl_2 Rate boosted than plateaus with Water

A stock solution of catalyst (0.0356 M) was prepared by dissolving [JohnphosAu(acetonitrile)]SbF_6 (772.17 g/mol, 68.9 mg, 0.089 mmol) in 2.50 mL of CD_2Cl_2. Urea alkene (336.48 g/mol, 12 mg, 0.0356 mmol) was dissolved in variable amount of CD_2Cl_2 and variable amount of H_2O, and then transferred to an NMR tube. Catalyst solution (25 µL, 0.00089 mmol) was injected to initiate the reaction and to bring the total volume to 0.7 mL (the substrate was at a concentration of 0.05 M); the sample was transferred to the NMR spectrometer and ^{1}H NMR spectra collected. The time from injection to spectrum #1 was approximately 5 minutes. Pseudo first order plots were linear up to at least 76% conversion.

	CD_2Cl_2	H_2O	Catalyst Solution	Catalyst Amount	Catalyst Load	$10^5 \times k_{obs}/s^{-1}$
BRL 2013	0.675 mL	5 µL	0.025 mL	0.00089 mmol	2.5 mol %	95 ± 3
BRL 2015	0.650 mL	25 µL	0.025 mL	0.00089 mmol	2.5 mol %	110 ± 3
BRL 2017	0.620 mL	55 µL	0.025 mL	0.00089 mmol	2.5 mol %	104 ± 3

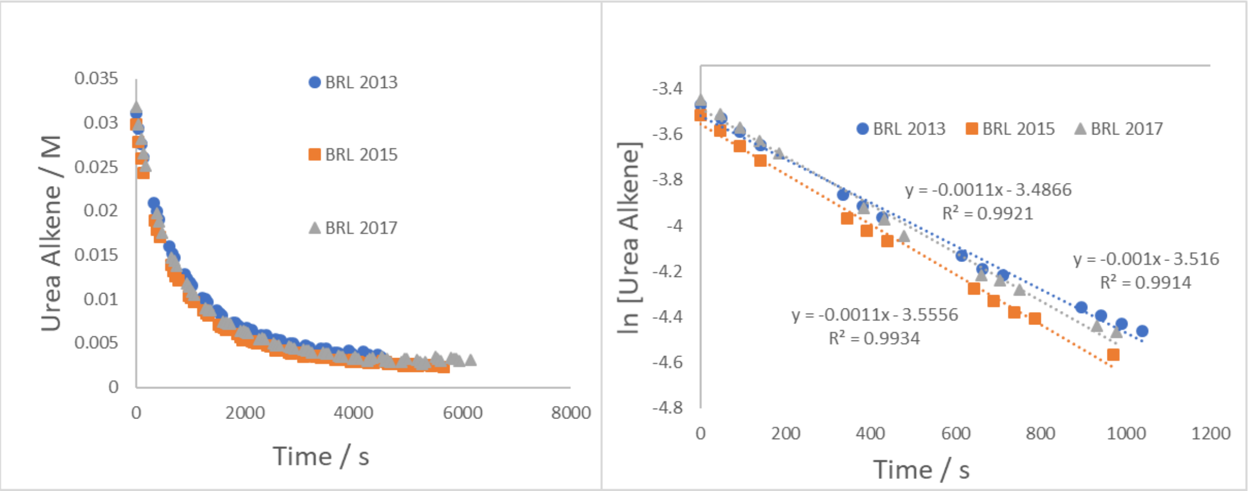

Figure S14. Raw and first order plots of urea alkene decay (0.05 M) in variable amount of CD_2Cl_2 and H_2O (total volume 0.7 mL) with 0.00089 mmol of [JPhosAu(acetonitrile)]SbF_6 catalyst (2.5 mol %).

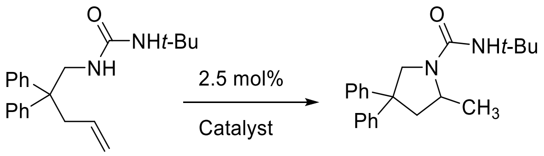

0.05 M

JPhosAuOTf (**4d**) in DCM, *Enhanced by Water*

JPhosAuOTf in CD_2Cl_2 Enhanced by Water

A stock solution of catalyst (0.0356 M) was prepared by dissolving JPhosAuOTf (664.44 g/mol, 36.2 mg, 0.054 mmol) in 1.53 mL of CD_2Cl_2 or by 73.2 mg (0.11 mmol) in 3.09 mL. Urea alkene (336.48 g/mol, 12 mg, 0.0356 mmol) was dissolved in variable amount of CD_2Cl_2 and variable amount of H_2O, and then transferred to an NMR tube. Some reactions utilized CD_2Cl_2 that was pre-treated with molecular sieves. Catalyst solution (25 µL, 0.00089 mmol) was injected to initiate the reaction and to bring the total volume to 0.7 mL (the substrate was at a concentration of 0.05 M); the sample was transferred to the NMR spectrometer and ^{1}H NMR spectra collected. The time from injection to spectrum #1 was approximately 5 minutes. Pseudo first order plots were linear up to at least 80% conversion.

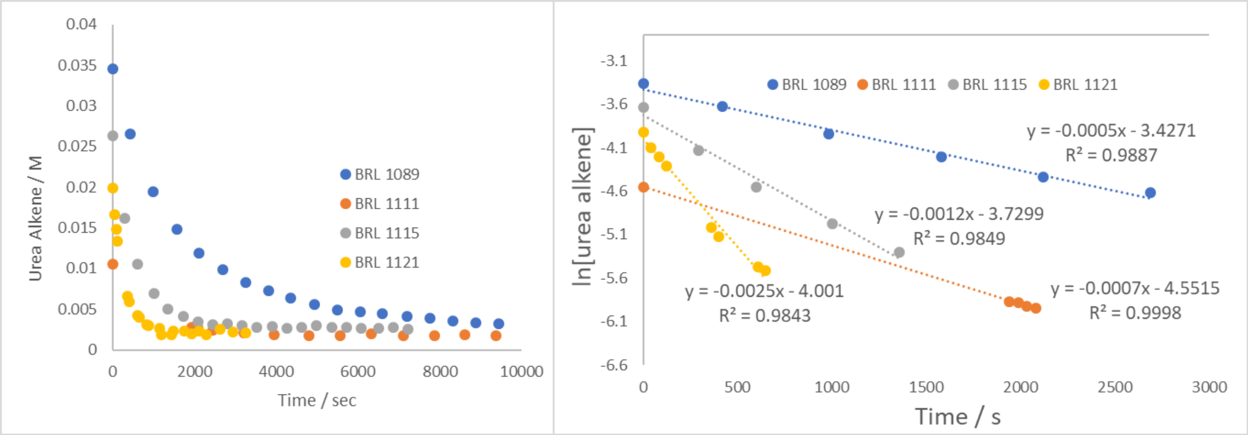

Figure S15. Raw and first order plots of urea alkene decay (0.05 M) in variable amount of CD_2Cl_2/H_2O (total volume 0.7 mL) with 0.00089 mmol of JPhosAuOTf catalyst (2.5 mol %).

	CD_2Cl_2	H_2O	Catalyst Solution	Catalyst Amount	Catalyst Loading	$10^5 \times k_{obs}/s^{-1}$
BRL 1089	0.675mL	none	0.025 mL	0.00089 mmol	2.5 mol %	47 ± 3
BRL 1111	0.675mL*	2μL	0.025 mL	0.00089 mmol	2.5 mol %	67.4 ± 0.6
BRL 1115	0.665mL*	10μL	0.025 mL	0.00089 mmol	2.5 mol %	121 ± 9
BRL 1121	0.635mL*	40μL	0.025 mL	0.00089 mmol	2.5 mol %	249 ± 13

*Solvent pre-treated with molecular sieves.

JPhosAuOTf in MeOH Diminished by Water

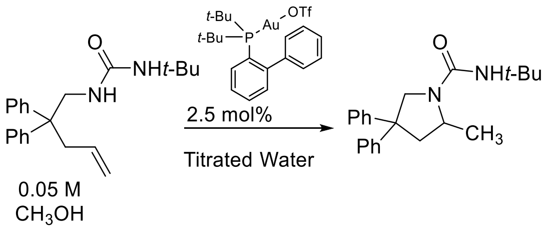

JPhosAuOTf (**4d**) in MeOH, *Diminished by Water*

A similar procedure was followed as in the previous section.

	MeOH	Water	Catalyst Solution	Catalyst Amount	Catalyst Loading	$10^5 \times k_{obs}/s^{-1}$
BRL 1143	0.675 mL	0 μL	0.025 mL	0.00089 mmol	2.5 mol %	48.2 ± 0.8
BRL 1141	0.65 mL	25 μL	0.025 mL	0.00089 mmol	2.5 mol %	19.5 ± 0.2
BRL 1145	0.635 mL	40 μL	0.025 mL	0.00089 mmol	2.5 mo l%	11.1 ± 0.4

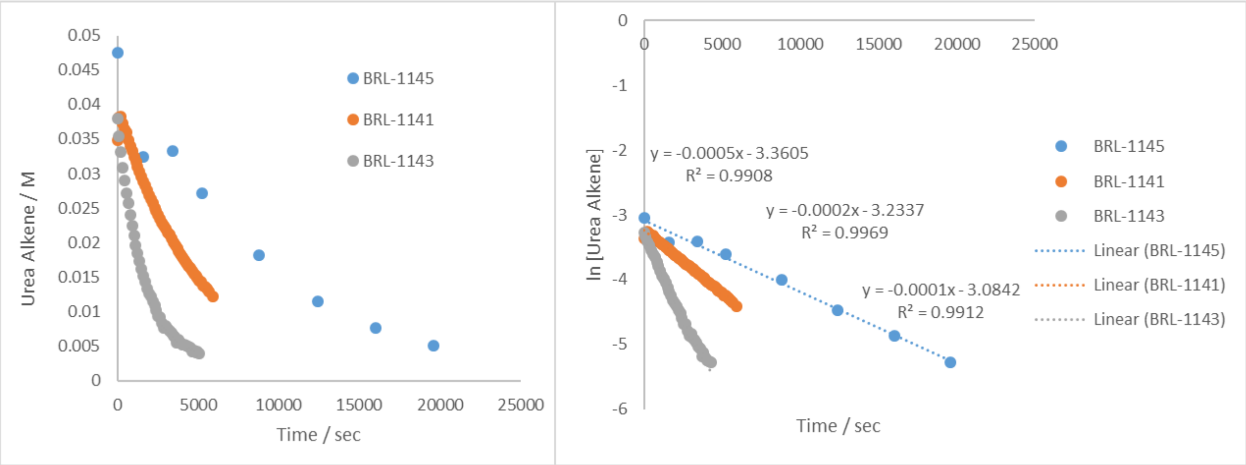

Figure S16. Raw and first order plots of urea alkene decay (0.05 M) in variable amount of CH_3OH/H_2O (total volume 0.7 mL) with 0.00089 mmol of JPhosAuOTf catalyst (2.5 mol %).

JPhosAu(NCCH$_3$)SbF$_6$ in MeOH Diminished by Water

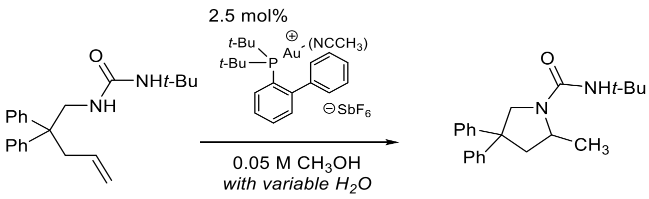

Diminished by water.

A similar procedure was followed. At 75 uL water, precipitates began to form, and the sample was turbid.

	MeOH	Water	Catalyst Solution	Catalyst Amount	Catalyst Loading	$10^5 \times k_{obs}/s^{-1}$
BRL 2039	0.65 mL	0 µL	0.05 mL	0.00089 mmol	2.5 mol %	50 ± 1
BRL 2041	0.625 mL	25 µL	0.05 mL	0.00089 mmol	2.5 mol %	11.5 ± 0.3
BRL 2043	0.575 mL	75 µL	0.05 mL	0.00089 mmol	2.5 mol %	6.5 ± 0.4

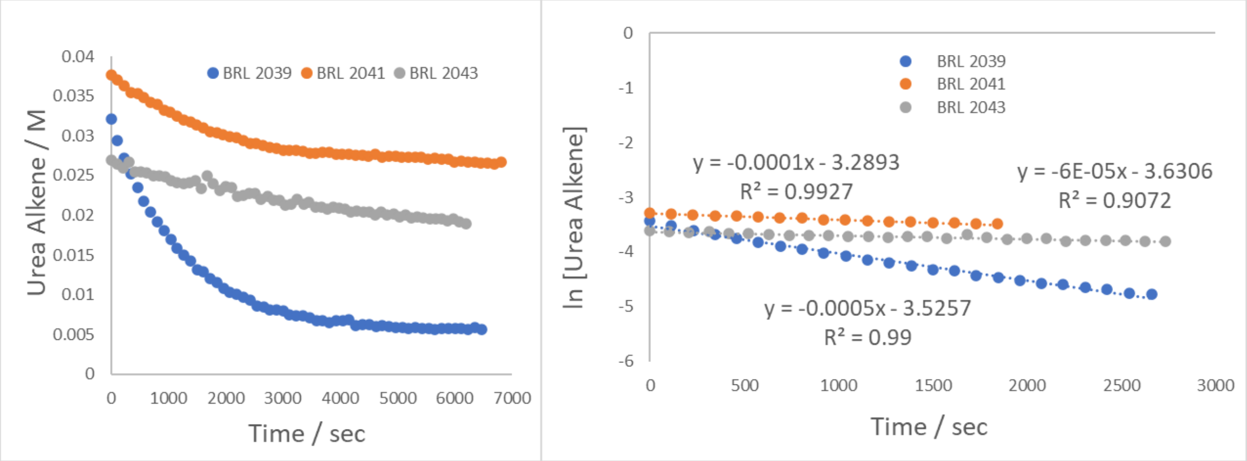

Figure S17. Raw and first order plots of urea alkene decay (0.05 M) in variable amount of CH_3OH/H_2O (total volume 0.7 mL) with 0.00089 mmol of JPhosAu($NCCH_3$)SbF_6 catalyst (2.5 mol %).

Reactions with Various Substrates (Table 4 in Paper)

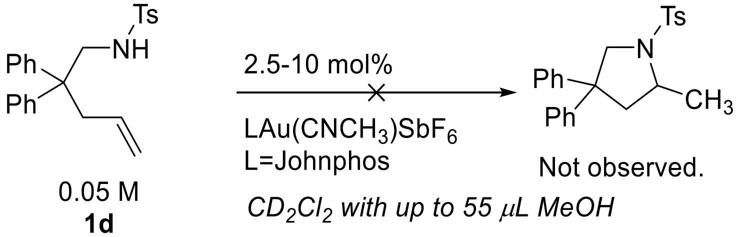

0.05 M
1d

Reaction with Sulfonamide (**1d**) and [JphosAu($NCCH_3$)]SbF_6

Sulfonamide **1d** (0.05 M, 0.7 mL CD_2Cl_2) did not react on measurable timescales at room temperature. No product was observed with up to 10 mol % JPhosAu($NCCH_3$)SbF_6 after 48 hours with and without added CH_3OH (up to 55 μL). The expected product was characterized previously.[S2]

Reaction with Carbamate (**1b**) and [JphosAu($NCCH_3$)]SbF_6

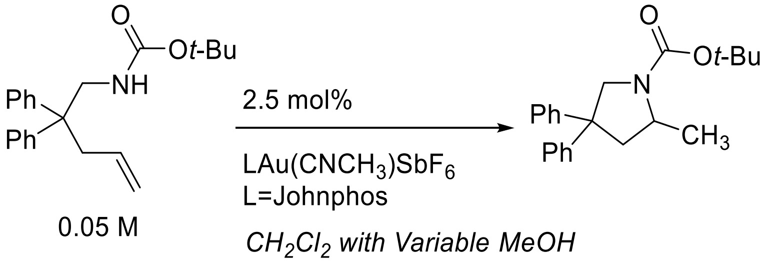

0.05 M

The carbamate starting material was prepared according to literature procedure[S3] and the cyclized carbamate product was characterized previously.[S2]

A stock solution of catalyst (0.036 M) was prepared by dissolving JPhosAu($NCCH_3$)SbF_6 (772.17 g/mol, 68.9 mg, 0.0892 mmol) in 2.5 mL of CH_2Cl_2. Carbamate alkene **1b** (337.46 g/mol, 12 mg, 0.036 mmol) was dissolved in variable amounts of CH_2Cl_2 with variable amounts of CH_3OH and transferred to an NMR tube. Catalyst solution (25 μL, 0.000892 mmol) was injected to initiate the reaction and to bring the total volume

to 0.7 mL (the substrate was at a concentration of 0.05 M); the sample was transferred to the NMR spectrometer and ^{1}H NMR spectra collected. The time from injection to spectrum #1 was approximately 5 minutes. Pseudo first order plots were linear up to at least ≈26% conversion, the reactions were not followed to completion. The cyclized product was characterized previously.

	CH_2Cl_2	CH_3OH	Catalyst Solution	Catalyst Amount	Catalyst Loading	$10^5 \times k_{obs}/s^{-1}$
BRL 2061	0.67 mL	5 µL	0.025 mL	0.0009 mmol	2.5 mol %	0.97 ± 0.02
BRL 2063	0.65 mL	25 µL	0.025 mL	0.0009 mmol	2.5 mol %	6.11 ± 0.09
BRL 2065	0.62 mL	55 µL	0.025 mL	0.0009 mmol	2.5 mol %	6.9 ± 0.2

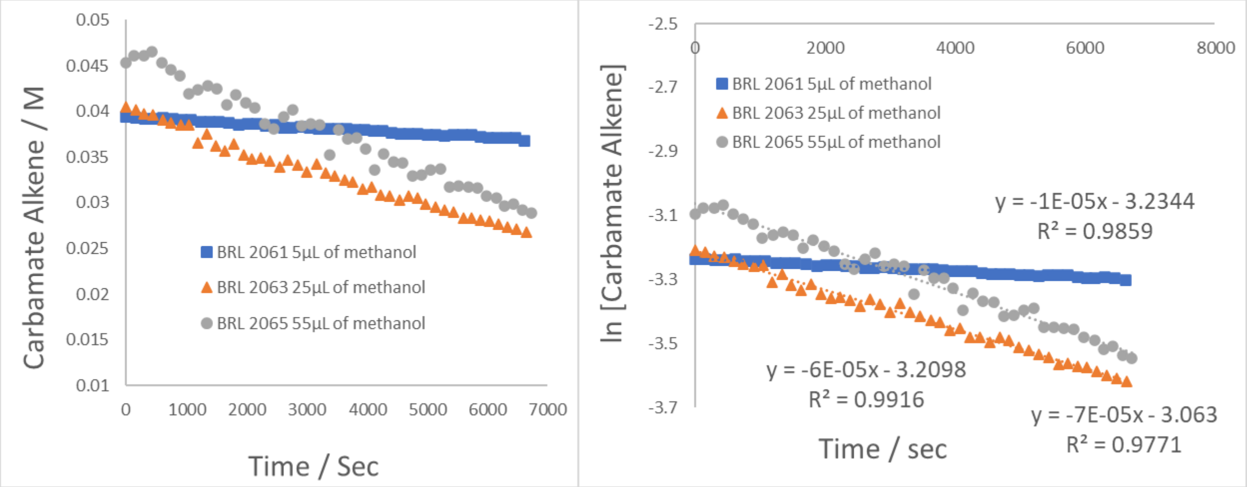

Figure S18. Raw plots of alkene carbamate decay (0.05 M) in variable amounts of CH_2Cl_2 and variable amounts of CH_3OH (total volume 0.7 mL) and with 0.0009 mmol of [JPhosAu($NCCH_3$)]SbF6 (2.5 mol %) and corresponding first order plots.

Reactions with Benzamide (**1c**)(Table 5 in Paper)

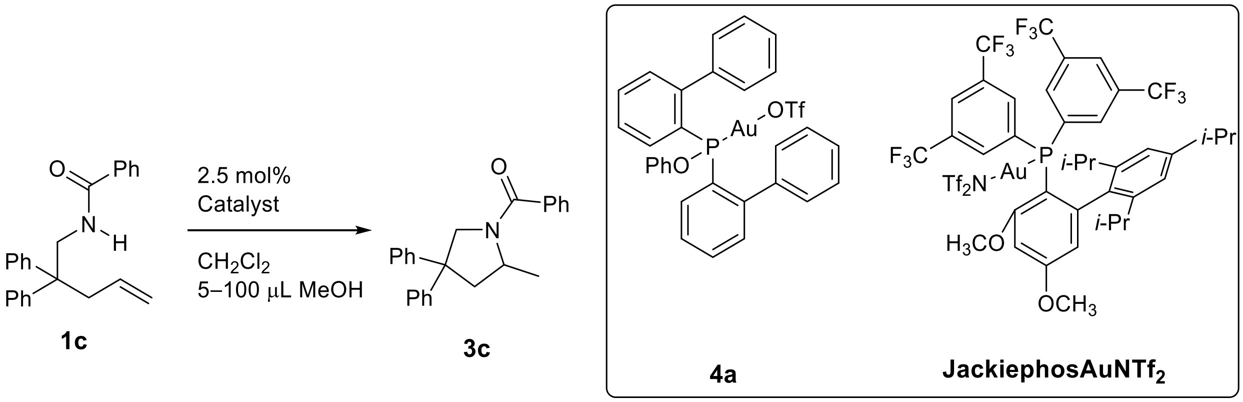

The benzamide starting material (**1c**) was prepared according to literature procedure, and the product (**3c**) was characterized previously.[S4]

Representative Procedure. Stock solutions of catalyst (0.035 M) were prepared in CH_2Cl_2. Alkene benzamide **1c** (341.45 g/mol, 12 mg, 0.0351 mmol) was dissolved in variable amounts of CH_2Cl_2 and variable amounts of CH_3OH, and then transferred to an NMR tube. Catalyst solution (25 µL, 0.00088 mmol) was injected to initiate the reaction and to bring the total volume to 0.7 mL (the substrate was at a concentration of 0.05 M); the sample was transferred to the NMR spectrometer and ^1H NMR spectra collected. The time from injection to spectrum #1 was approximately 5 minutes. Pseudo first order plots were linear up to the end of acquisition of data (40-70% for **4a**). Pseudo first order plots were linear up to at least 52% conversion for JackiphosAuNTf2.

	CH_2Cl_2	CH_3OH	Catalyst Solution	Catalyst Amount	Catalyst Loading	$10^5 \times K_{obs}/s^{-1}$
BRL-2113 (Catalyst **5**)	0.595 mL	55 µL	0.050 mL	0.00088 mmol	2.5 mol %	Plot not shown. 14% Conversation at 24 hours
BRL 3033 (Catalyst **4a**)	0.670 mL	5 µL	0.025 mL	0.00088 mmol	2.5 mol %	0.158 ± 0.001
BRL 3035 (Catalyst **4a**)	0.650 mL	25 µL	0.025 mL	0.00088 mmol	2.5 mol %	0.36 ± 0.02
BRL 3041 (Catalyst **4a**)	0.620 mL	55 µL	0.025 mL	0.00088 mmol	2.5 mol %	0.37 ± 0.02
BRL 3055 (JackiephosAuNTf$_2$)	0.620 mL	55 µL	0.025 mL	0.00088 mmol	2.5 mol %	1.02 ± 0.09
BRL3059 (JackiephosAuNTf$_2$)	0.575 mL	100 µL	0.025 mL	0.00088 mmol	2.5 mol %	0.82 ± 0.07 (Plot not shown)

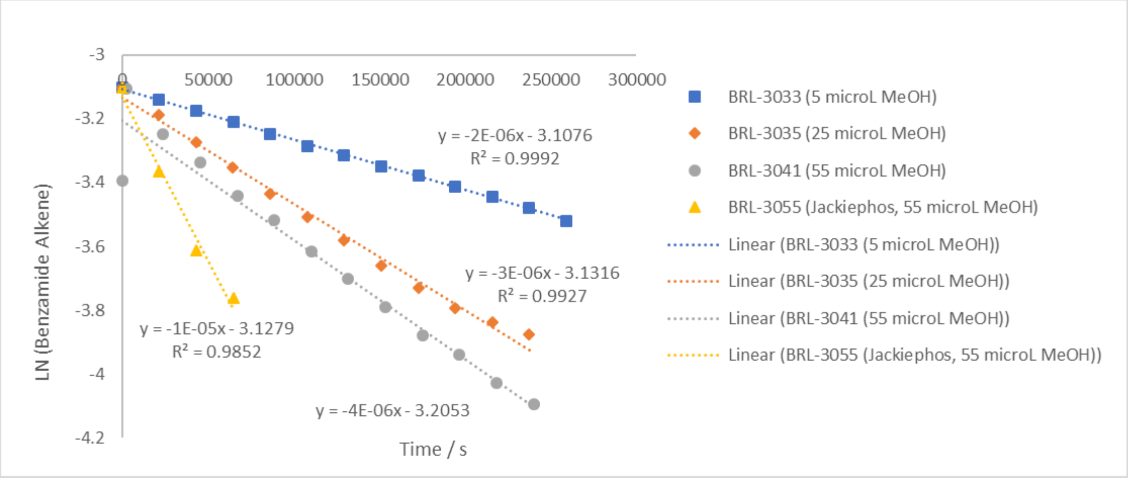

Figure S19. First order plots of alkene carbamate decay (0.05 M) in variable amounts of CH_2Cl_2 and CH_3OH (total volume 0.7 mL) and with 0.0009 mmol (2.5 mol %) of catalysts JackiephosAuNTf2 and **4a**.

Examining Decomposition in Reaction with carbamate (1b) and Catalysts 4c and 4a

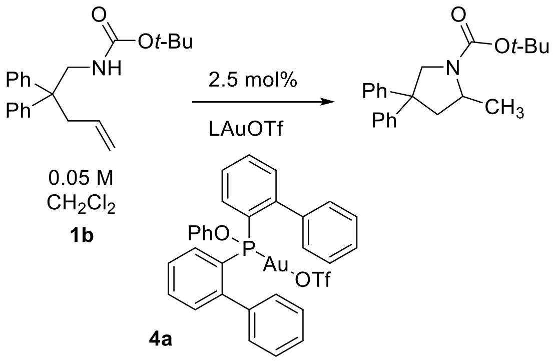

A stock solution of catalyst (0.036 M) was prepared by dissolving catalyst **4a** (776.52 g/mol, 99.7 mg, 0.128 mmol) in 3.67 mL of CH_2Cl_2. Carbamate alkene **1b** (337.46 g/mol, 12 mg, 0.036 mmol) was dissolved in variable amounts of CH_2Cl_2 and variable amounts of CH_3OH and transferred to an NMR tube. Catalyst solution (25 μL, 0.000889 mmol) was injected to initiate the reaction and to bring the total volume to 0.7mL (the substrate was at a concentration of 0.05 M); the sample was transferred to the NMR spectrometer and ^{1}H NMR spectra collected. The time from injection to spectrum #1 was approximately 5 minutes. Pseudo first order plots were linear up to at least 34% conversion (no methanol) and 80% conversion (with methanol).

	CH_2Cl_2	CH_3OH	Catalyst Solution	Catalyst Amount	Catalyst Loading	$10^5 \times k_{obs}/s^{-1}$
BRL 2071	0.675 mL	no	0.025 mL	0.0009 mmol	2.5 mol %	1.981 ± 0.007
BRL 2073	0.670 mL	5 μL	0.025 mL	0.0009 mmol	2.5 mol %	17.01 ± 0.08
BRL 2075	0.650 mL	25 μL	0.025 mL	0.0009 mmol	2.5 mol %	21.7 ± 0.1

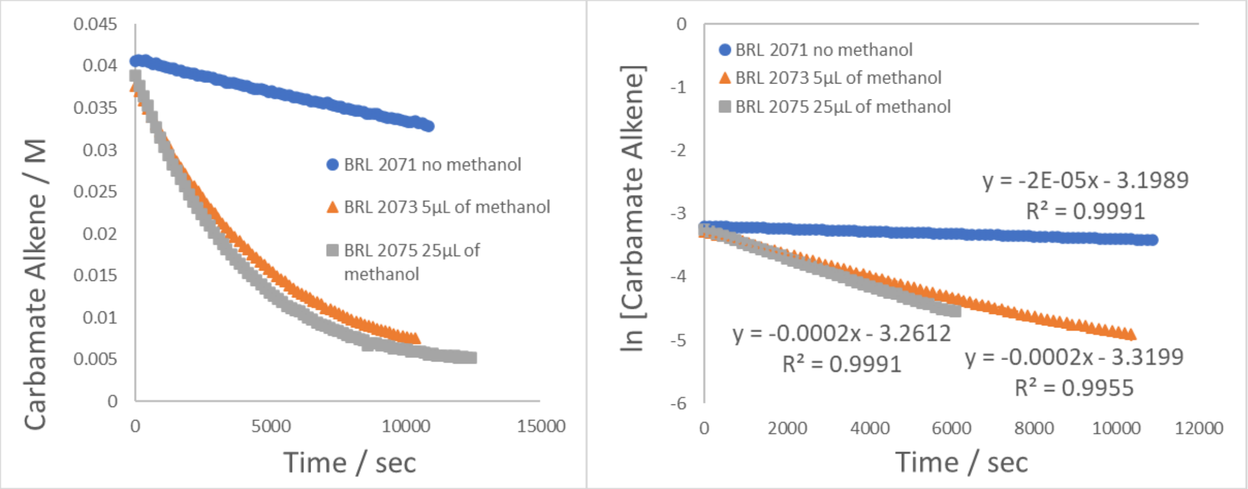

Figure S20. Raw plots of alkene carbamate decay (0.05 M) in variable amounts of CH_2Cl_2 and CH_3OH (total volume 0.7 mL) with 0.0009 mmol of **4a** (2.5 mol %) and the corresponding first order plots.

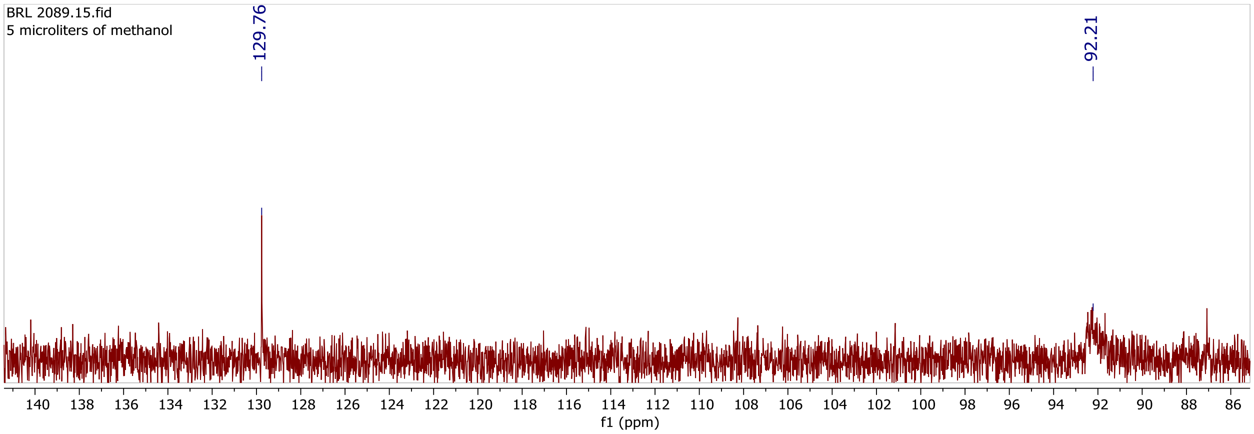

Figure S21. ^{31}P NMR Spectrum (161.98 MHz, CH_2Cl_2) at approximately 50% conversion during reaction of alkene carbamate (0.05 M) in CH_2Cl_2 with 5 μL CH_3OH (total volume 0.7mL) and **4a** (2.5mol %).

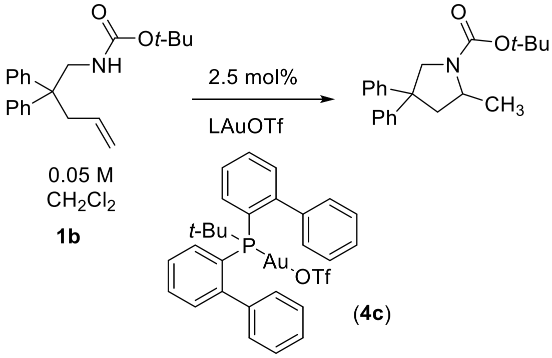

A stock solution of catalyst (0.036 M) was prepared by dissolving **4c** (740.53g/mol, 133.8 mg, 0.181 mmol) in 5.08 mL of CH_2Cl_2. Carbamate alkene **1b** (337.46g/mol, 12 mg, 0.036 mmol) was dissolved in variable amounts of CH_2Cl_2 and variable amounts of CH_3OH and transferred to an NMR tube. Catalyst solution (25 μL, 0.000892 mmol) was injected to initiate the reaction and to bring the total volume to 0.7 mL (the substrate was at a concentration of 0.05 M); the sample was transferred to the NMR spectrometer and ^1H NMR spectra collected. The time from injection to spectrum #1 was approximately 5 minutes. Pseudo first order plots were linear up to 20% conversion (no methanol) and ≈80% conversion (with methanol).

	CH_2Cl_2	CH_3OH	Catalyst Solution	Catalyst Amount	Catalyst Loading	$10^5 \times k_{obs}/s^{-1}$
BRL 2081	0.675 mL	0	0.025 mL	0.0009 mmol	2.5 mol %	0.205 ± 0.009
BRL 2083	0.670 mL	5 μL	0.025 mL	0.0009 mmol	2.5 mol %	8.58 ± 0.01
BRL 2085	0.650 mL	25 μL	0.025 mL	0.0009 mmol	2.5 mol %	12.68 ± 0.07

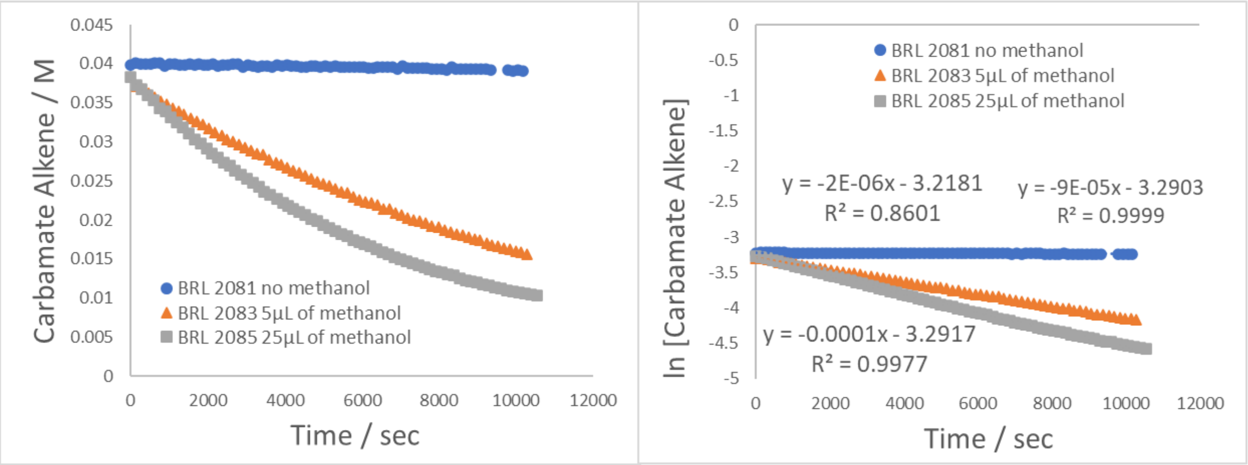

Figure S22. Raw and first order plots of carbamate alkene **1b** decay (0.05 M) in variable amounts of CH_2Cl_2 and CH_3OH (total volume 0.7mL) and with 0.0009 mmol of catalyst **4c** (2.5mol %).

Bisphos Syntheses, ^{31}P Chemical Shifts for Decomposition Byproducts

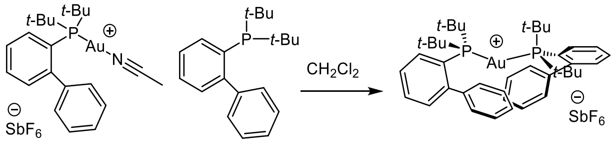

JPhos Gold Bisphos. Gold acetonitrile (0.3 g, 0.39 mmol) was weighed into a vial and ligand (0.156 g, 0.52 mmol) was weighed into a separate flask. DCM (6 mL) was added to the gold acetonitrile and the solution transferred to the ligand. Additional DCM was used to rinse the vial to complete the transfer of gold acetonitrile to the flask. The solution was stirred for 30 minutes. The solvent was removed in vacuo and NMR spectra collected. The solid was recrystallized with 2:1 Hexanes/DCM at −20 degrees C (yield not determined).

See manuscript for ^{31}P NMR spectrum (Figure 4).

Jackiephos Bisphos. The ^{31}P chemical shift for the decomposition product of Jackiephos gold was confirmed by treating L-AuNTf₂ l with 1 equivalent of free ligand in CD_2Cl_2.

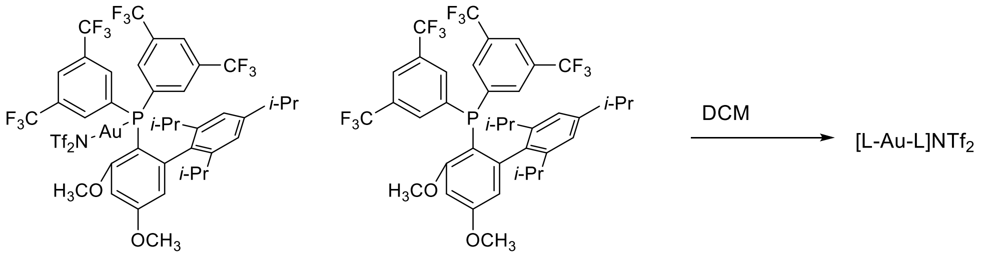

14.2 ppm **-15.8 ppm**

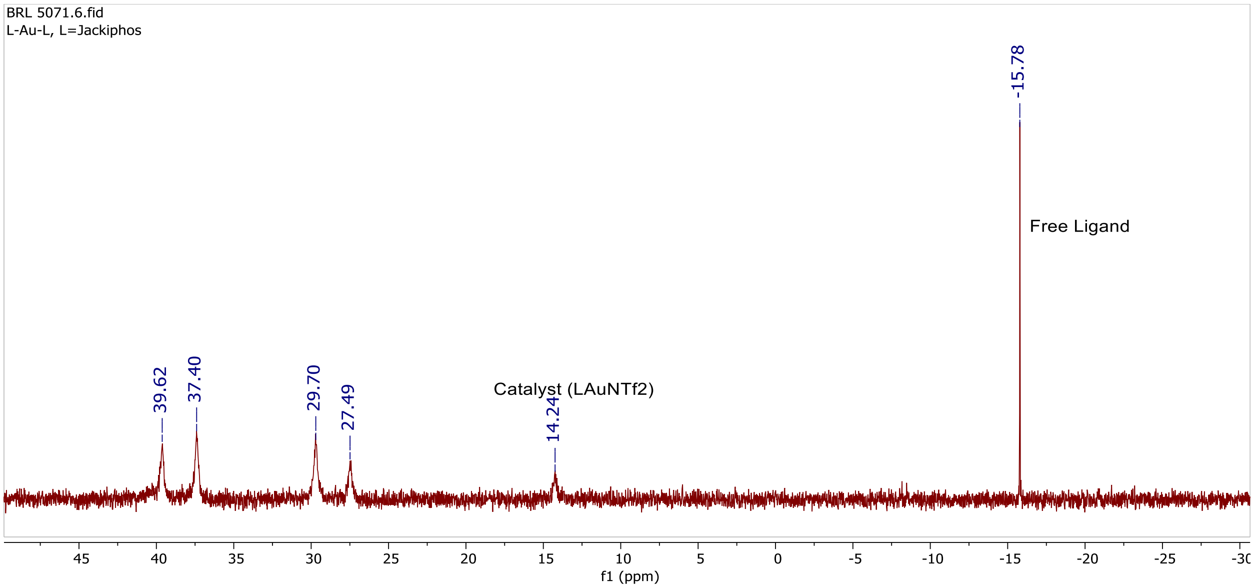

Figure S23. ^{31}P NMR Spectrum (161.98 MHz, CD_2Cl_2) after combining JackiephosAuNTf$_2$ with free Jackiephos ligand.

Bisphos (Catalyst 4a). The ^{31}P chemical shift for the decomposition product of **4a** was confirmed by treating L-Au-Cl with 1 equivalent of free ligand in the presence of $AgSb_6$ in CH_2Cl_2.

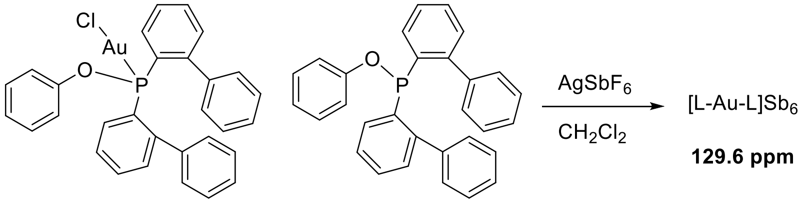

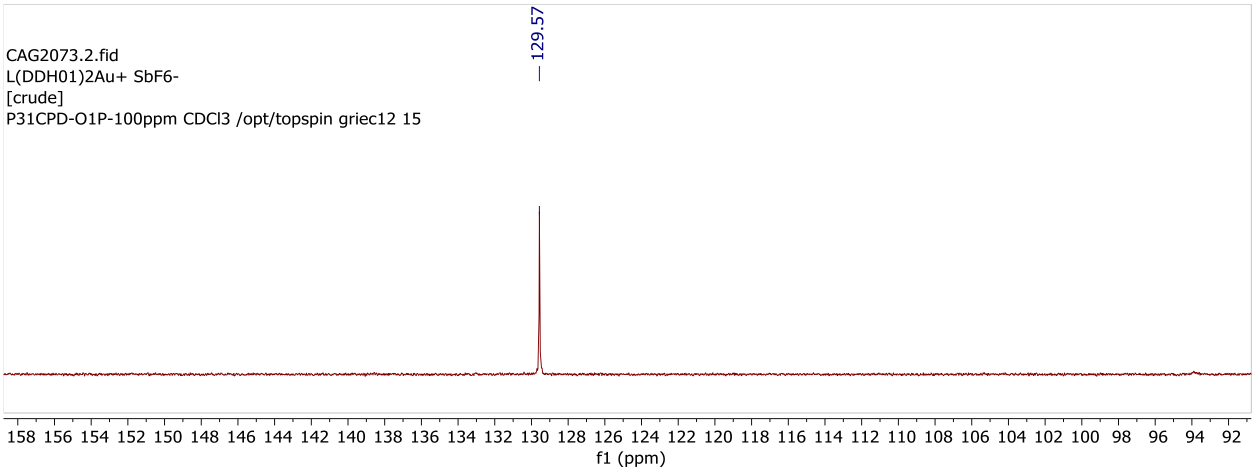

Figure S24. ^{31}P NMR Spectrum (121.51 MHz, CD_2Cl_2) after combining LAuCl, free ligand, and $AgSBF_6$.

Reactions with tris(2,4-di-tert-butylphenyl)phosphite ligand.

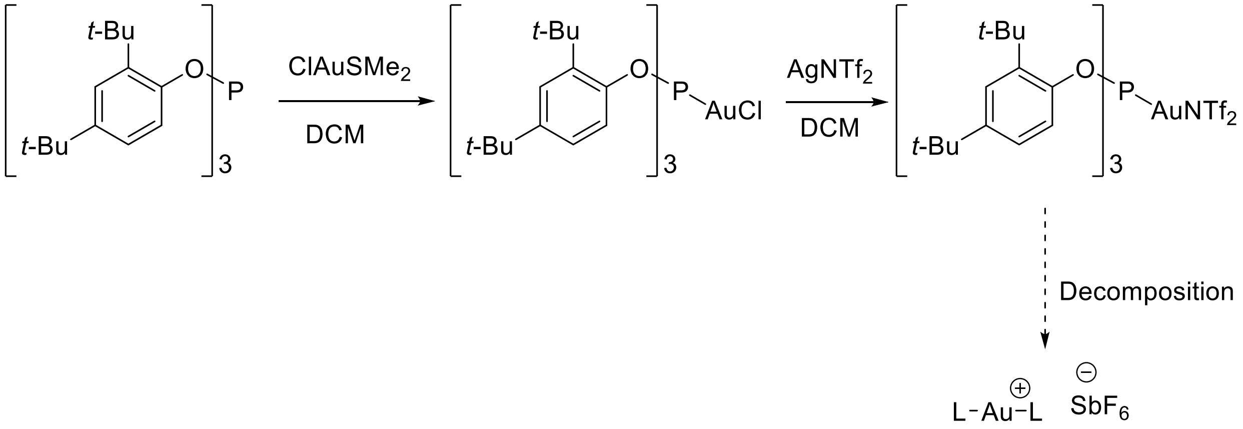

SbF_6 salt: 119 ppm
(Ref. S5)

The decomposition product $[(R_3P\text{-}Au\text{-}PR_3)]SbF_6$ was reported previously.[S5] The L-Au-NTf_2 catalyst was reported previously.[S6] The L-Au-Cl precatalyst was reported previously.[S7]

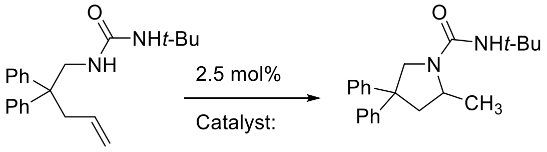

1a **3a**
0.05 M
Solvent Screen

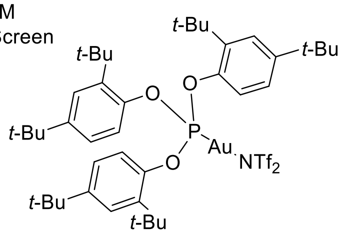

tris(2,4-di-tert-butylphenyl)phosphiteAuNTf_2 (**6b**)

When tris(2,4-di-tert-butylphenyl)phosphiteAuNTf2 (**6b**) was tested as a catalyst it showed moderate activity, but near complete formation of decomposed product $[(R_3P\text{-}Au\text{-}PR_3)]NTf_2$ as indicated by the near identical ^{31}P chemical shift to the SbF_6 variant reported previously.

A stock solution of catalyst (**6b**) was prepared by dissolving **6b** (90.2 mg, 1124.03 g/mol, 0.080 mmol) in 2.28 mL of CH_2Cl_2. Urea Alkene **1a** (336.48 g/mol, 12 mg, 0.0356 mmol) was dissolved in variable amounts of CH_2Cl_2 and variable amounts of CH_3OH and transferred to an NMR tube. Catalyst solution (25 µL, 0.00088 mmol) was injected to initiate the reaction and to bring the total volume to 0.7 mL (the substrate was at a concentration of 0.05 M); the sample was transferred to the NMR spectrometer and ^1H NMR spectra collected. The time from injection to spectrum #1 was approximately 5 minutes. Pseudo first order plots were linear up to ≈84% conversion.

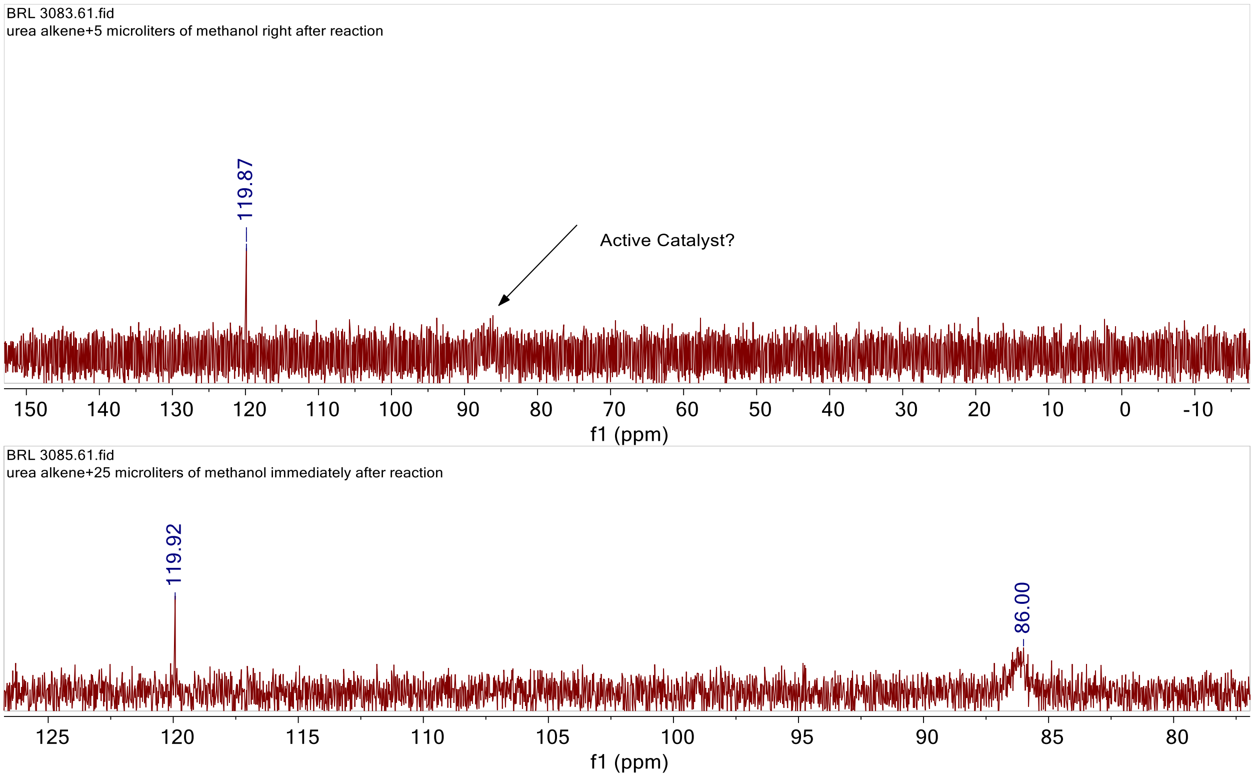

Figure S25. NMR spectra (^{31}P, 161.98 MHz) that show the decomposition of the catalyst **6b** (δ=119.9 ppm, 2.5 mol %) during the reaction of urea alkene (**1a**) (0.05 M, CH_2Cl_2) with 5 and 25 μL MeOH.

	CH_2Cl_2	CH_3OH	Catalyst Solution	Catalyst Amount	Catalyst Loading	$10^5 \times k_{obs}/s^{-1}$
BRL 3083	0.670 mL	5 μL	0.025 mL	0.00088 mmol	2.5 mol %	65 ± 1
BRL 3085	0.650mL	25 μL	0.025 mL	0.00088 mmol	2.5 mol %	69 ± 1

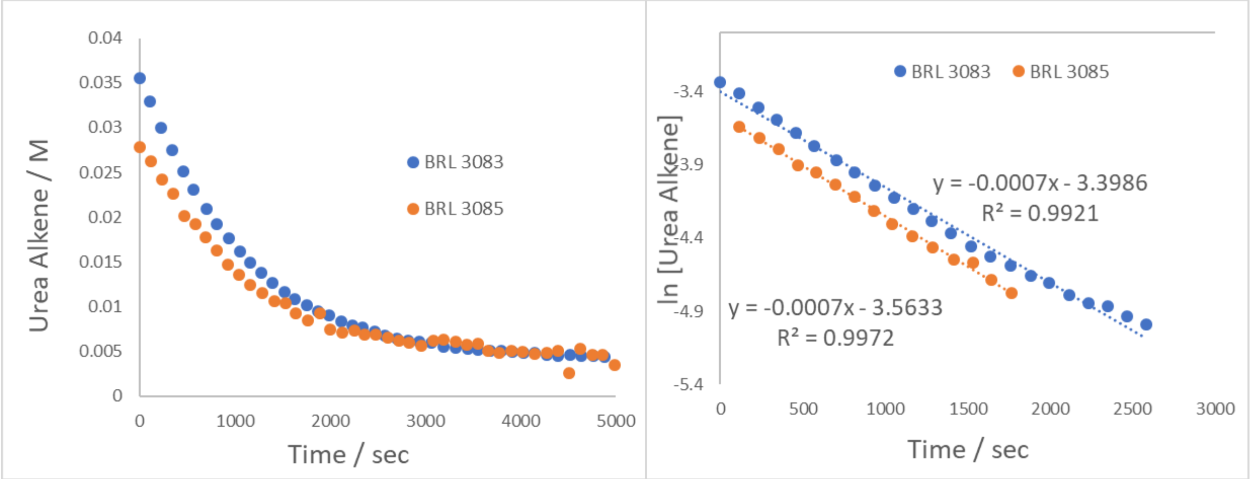

Figure S26. Raw plots of urea alkene **1a** decay (0.05 M) in 0.7 mL of CH_2Cl_2 with 0.00088 mmol of **6b** (2.5 mol %) and the corresponding first order plots.

Kinetic Isotope Effect Measurements (Table 7 and 8 In paper)

Urea with Titrated CH_3OD (Compare to Figure S13 for KIE)

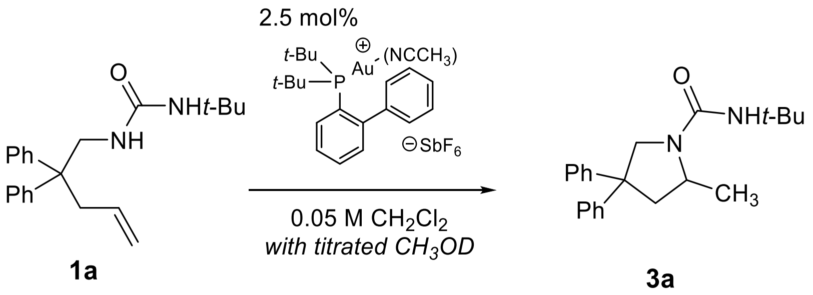

A stock solution of catalyst (0.036 M) was prepared by dissolving JPhosAu($NCCH_3$)SbF_6 (772.17 g/mol, 68.9 mg, 0.0892 mmol) in 2.5 mL of CH_2Cl_2. Urea alkene **1a** (336.48 g/mol, 12 mg, 0.036 mmol) was dissolved in variable amount of CH_2Cl_2 with variable amount of CH_3OD and transferred to an NMR tube. Catalyst solution (25 µL, 0.000892 mmol) was injected to initiate the reaction and to bring the total volume to 0.7mL (the substrate was at a concentration of 0.05 M); the sample was transferred to the NMR spectrometer and ^{1}H NMR spectra collected. The time from injection to spectrum #1 was approximately 5 minutes. Pseudo first order plots were linear up to at least 52% conversion.

	CH_2Cl_2	CH_3OD	Catalyst Solution	Catalyst Amount	Catalyst Loading	$10^5 \times k_{obs}/s^{-1}$
BRL 2047	0.67 mL	5 µL	0.025 mL	0.0009 mmol	2.5 mol %	70 ± 2
BRL 2049	0.65 mL	25 µL	0.025 mL	0.0009 mmol	2.5 mol %	130 ± 10
BRL 2051	0.62 mL	55 µL	0.025 mL	0.0009 mmol	2.5 mol %	81 ± 1

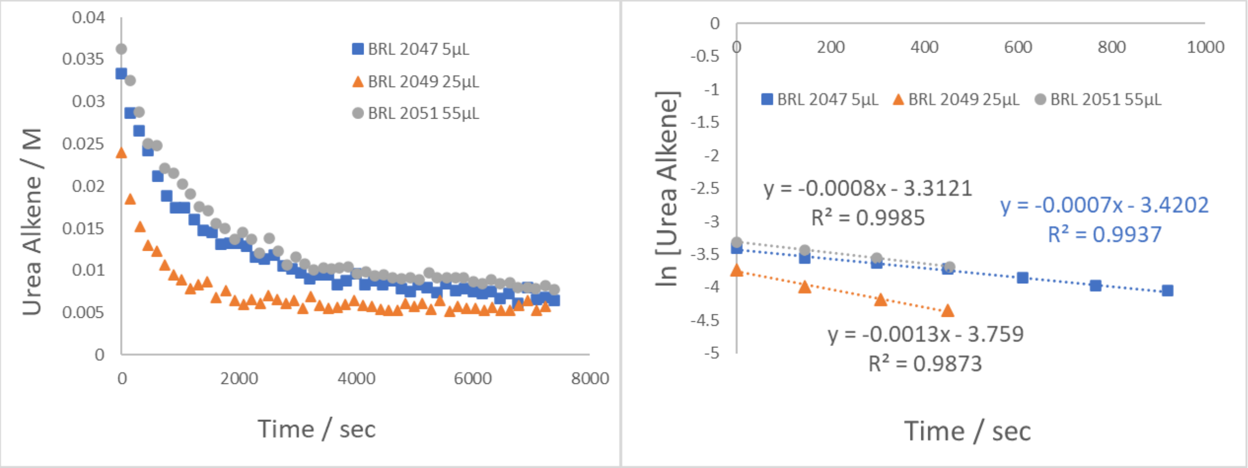

Figure S27. Raw plots of urea alkene decay (0.05 M) in variable amounts of CH_2Cl_2 and variable amounts of CH_3OD (total volume 0.7 mL) and with 0.0009 mmol of [JPhosAu($NCCH_3$)]SbF_6 (2.5 mol %) and first order plots of alkene decay.

Preparation of Partially Deuterated Urea and Rate

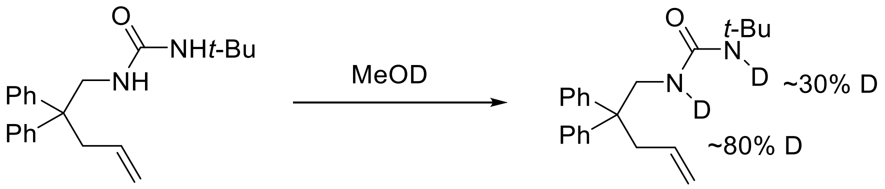

Partially deuterated urea alkene was prepared according to the following procedure. 75 mg of urea alkene (0.075 g, 336.48 g/mol, 0.22 mmol) was dissolved in 2.5 mL of CH_3OD and stirred for 24 hours before the solvent, CH_3OD, was removed via high vacuum. This was repeated 3 times. Partially deuterated **1a** was isolated (0.065 g, ≈86%). The N-H adjacent to the *t*-butyl group incorporated less deuterium than the N-H adjacent to the methylene as determined by ^1H NMR integration.

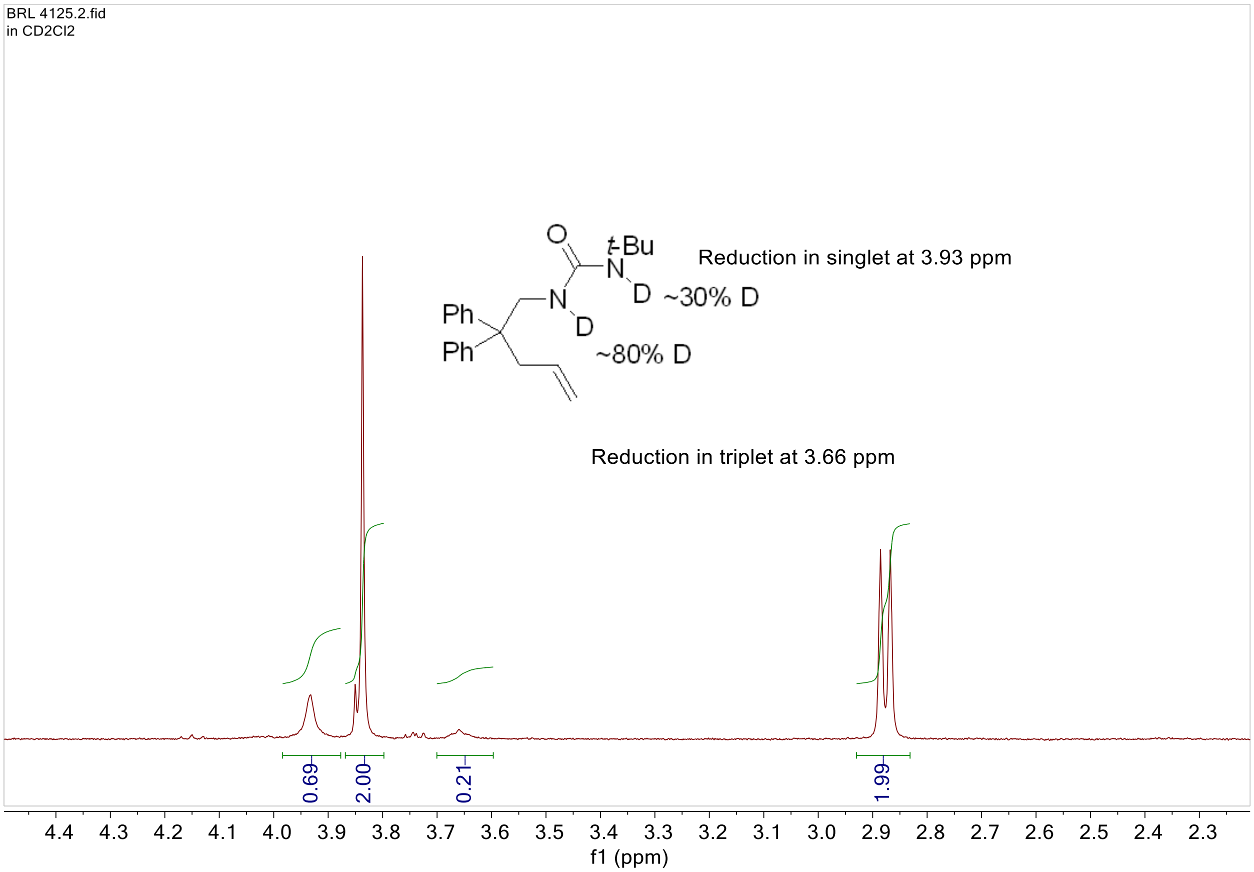

Figure S28. Expansion showing partial N-H deuterium incorporation (500 MHz, CD_2Cl_2).

Reaction of Partially Deuterated Urea with [JphosAu($NCCH_3$)]SbF_6

A stock solution of catalyst (0.036 M) was prepared by dissolving JPhosAu($NCCH_3$)SbF_6 (772.17 g/mol, 68.9 mg, 0.0892 mmol) in 2.5 mL of CD_2Cl_2. Deuterated urea Alkene (≈337.48g/mol, 12 mg, 0.036 mmol) was dissolved in 675 µL of CD_2Cl_2 and transferred to an NMR tube. Catalyst solution (25 µL, 0.000892 mmol) was injected to initiate the reaction and to bring the total volume to 0.7 mL (the substrate was at a concentration of 0.05 M); the sample was transferred to the NMR spectrometer and ^1H NMR spectra

collected. The time from injection to spectrum #1 was approximately 5 minutes. Pseudo first order plots were linear up to at least 60% conversion. In all cases, 30-60% deuterium incorporation was observed in the product methyl (δ 20.96 ppm, 1:1:1 triplet) and the incorporation amount estimated by ^{13}C NMR integration.

	CD_2Cl_2	Catalyst Solution	Catalyst Amount	Catalyst Loading	10^5 x k_{obs}/s^{-1}	*k**obs* average
BRL 4127	0.675 mL	0.025 mL	0.000892 mmol	2.5 mol %	41 ± 1	48±8
BRL 4129	0.675 mL	0.025 mL	0.000892 mmol	2.5 mol %	45 ± 1	
BRL 4131	0.675 mL	0.025 mL	0.000892 mmol	2.5 mol %	57 ± 2	

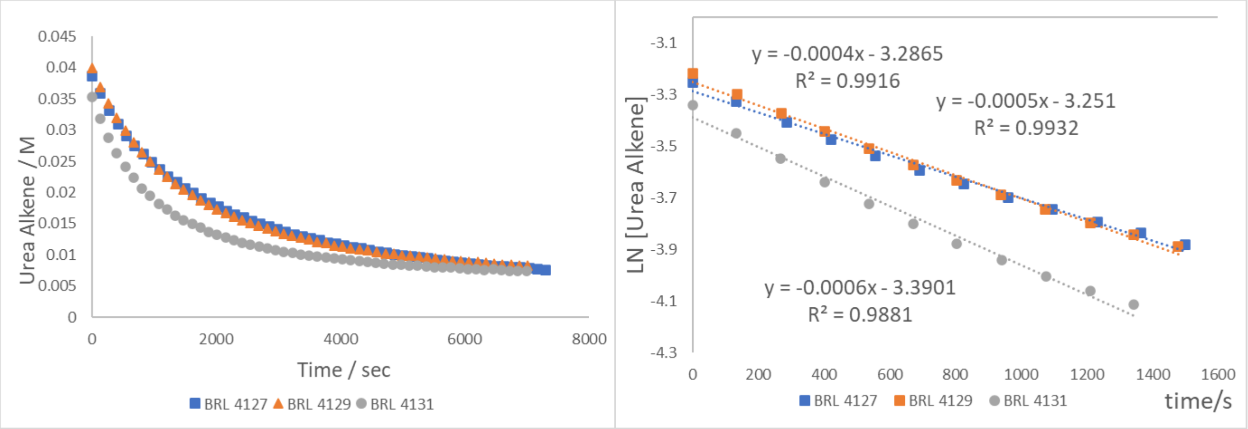

Figure S29. Raw and first order plots of monodeuterated urea alkene decay (0.05 M) in 0.7mL of CH_2Cl_2 and with 0.00089 mmol of [JPhosAu(NCCH$_3$)]SbF$_6$ (2.5 mol %).

Carbamate with Titrated CD_3OD (Compare to Figure S18 for KIE)

The same procedure applied to the kinetic experiments adopting CD_3OD as the additive. Pseudo first order plots were linear up to at least ≈20% conversion, but the reactions were not followed to completion.

	CH_2Cl_2	CD_3OD	Catalyst Solution	Catalyst Amount	Catalyst Loading	$10^5 \times k_{obs}$/s^{-1}
BRL 5075	0.67 mL	5 µL	0.025 mL	0.0009 mmol	2.5 mol %	0.54 ± 0.02
BRL 5077	0.65 mL	25 µL	0.025 mL	0.0009 mmol	2.5 mol %	2.15 ± 0.01
BRL 5079	0.62 mL	55 µL	0.025 mL	0.0009 mmol	2.5 mol %	1.76 ± 0.02

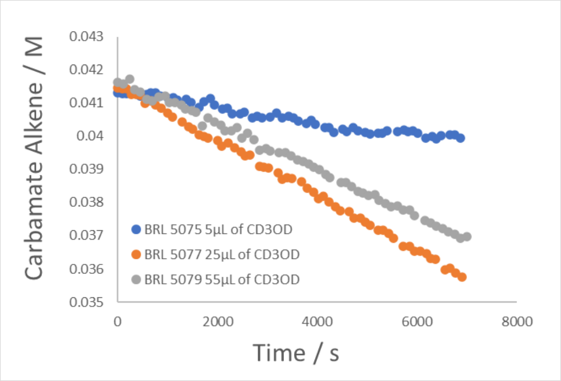

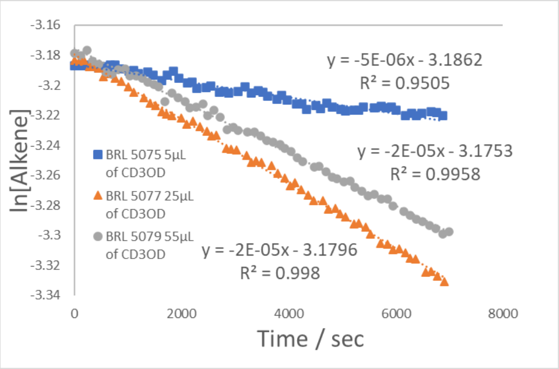

Figure S30. Raw plots of alkene decay (0.05 M) in variable amounts of CH_2Cl_2 and variable amounts of CD_3OD (total volume 0.7 mL) and with 0.0009 mmol of [JPhosAu($NCCH_3$)]SbF_6 (2.5 mol %) and the corresponding first order plots.

Solvent Deuterium Isotope Effects (Carbamate/Johnphos)

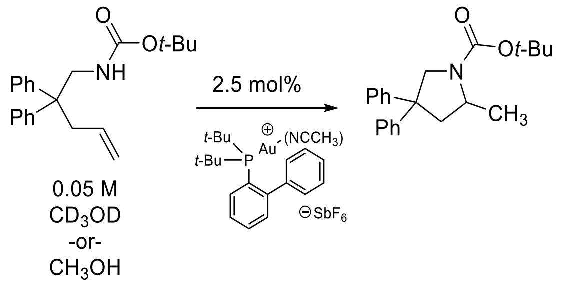

Solvent k(H/D) = 6.3

Pure CD_3OD Solvent

	Catalyst Solution	Catalyst Amount	Catalyst Loading	$10^5 \times k_{obs}/s^{-1}$	
BRL 4039	0.025 mL	0.0009 mmol	2.5 mol %	0.130 ± 0.002	Average<br>0.127 ± 0.003
BRL 4041	0.025 mL	0.0009 mmol	2.5 mol %	0.126 ± 0.002	
BRL 4043	0.025 mL	0.0009 mmol	2.5 mol %	0.125 ± 0.002	

Pure CH_3OH Solvent

	Catalyst Solution	Catalyst Amount	Catalyst Loading	$10^5 \times k_{obs}/s^{-1}$	
BRL 4045	0.025 mL	0.0009 mmol	2.5 mol %	0.9 ± 0.1	Average<br>0.8 ± 0.1
BRL 4047	0.025 mL	0.0009 mmol	2.5 mol %	0.7 ± 0.1	
BRL 4049	0.025 mL	0.0009 mmol	2.5 mol %	NA/peak overlap	

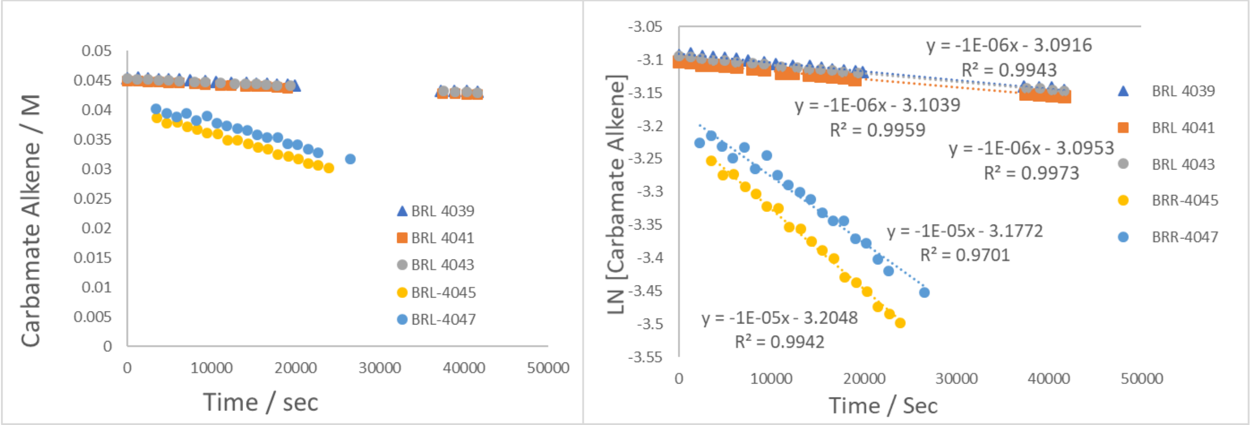

Figure S31. Raw and first order plots of carbamate alkene decay (0.05 M) in 0.7 mL of CH_3OH or CD_3OD and with 0.0009 mmol of $[JPhosAu(NCCH_3)]SbF_6$ (2.5 mol %).

Solvent Deuterium Isotope Effects (Urea/Jackiephos)

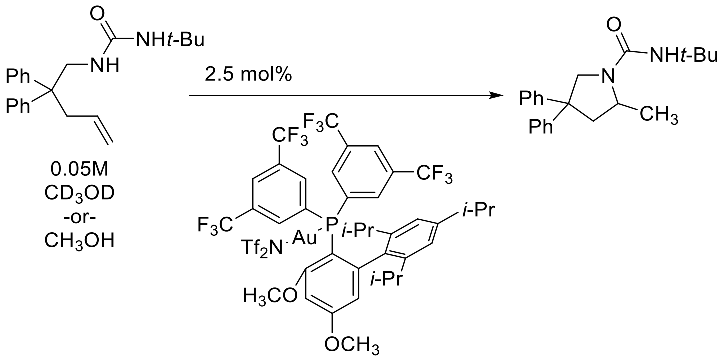

Solvent k(H/D) = 7.4

Pure CH_3OH Solvent

	CH_3OH	Catalyst Solution	Catalyst Amount	Catalyst Loading	$10^5 \times k_{obs}/s^{-1}$	
BRL 4057	0.65 mL	0.05 mL	0.00089 mmol	2.5 mol %	26.2 ± 0.6	Average 23 ± 5
BRL 4059	0.65 mL	0.05 mL	0.00089 mmol	2.5 mol %	16.7 ± 0.2	
BRL 4061	0.65 mL	0.05 mL	0.00089 mmol	2.5 mol %	25.9 ± 0.6	

See earlier Figure S12. for plots.

Pure CD_3OD Solvent

	CD_3OD	Catalyst Solution	Catalyst Amount	Catalyst Loading	$10^5 \times k_{obs}/s^{-1}$	
BRL 4065	0.65 mL	0.050 mL	0.0009 mmol	2.5 mol %	2.05 ± 0.02	Average 3.1 ± 0.9
BRL 4067	0.65 mL	0.050 mL	0.0009 mmol	2.5 mol %	3.57 ± 0.01	
BRL 4069	0.65 mL	0.050 mL	0.0009 mmol	2.5 mol %	3.686 ± 0.007	

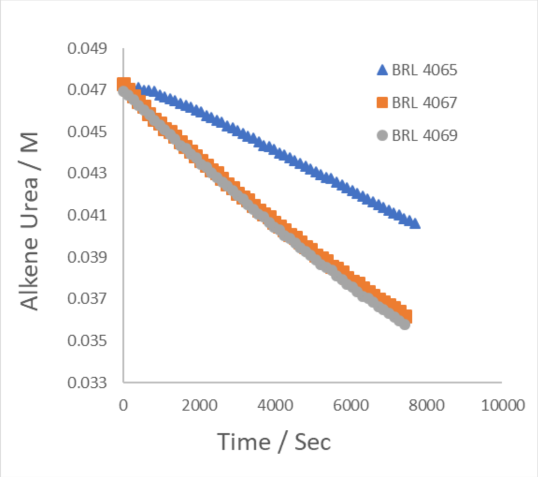

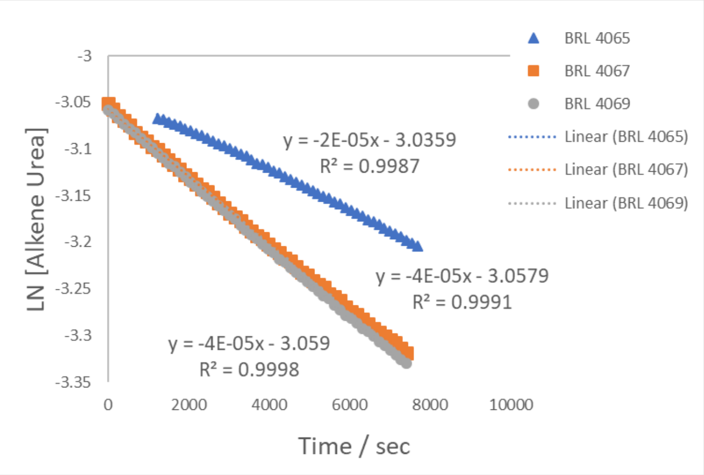

Figure S32. Raw and first order plots of urea alkene decay (0.05 M) in 0.7 mL of CD_3OD and with 0.0009 mmol of Jackiephos-Au-NTf_2 (2.5 mol %).

Other Additives (Figure 6 in Paper)

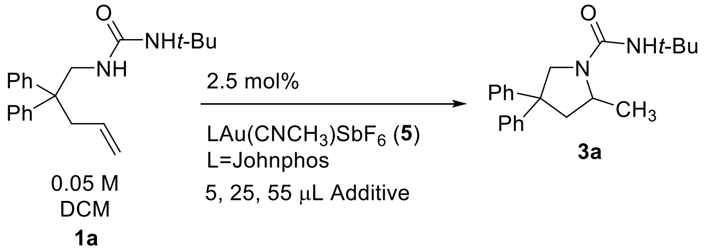

A stock solution of catalyst (0.036 M) was prepared by dissolving [JPhosAu(NCCH3)]SbF_6 (772.17 g/mol, 138 mg, 0.179 mmol) in 5mL of CH_2Cl_2. Urea alkene (336.48 g/mol, 12 mg,0.0356 mmol) was dissolved in variable amounts of CH_2Cl_2 and variable amounts of additives, including but not limited to ethanol, n-propanol, isopropanol, hexafluoroisopropanol (HFIP), etc., and then transferred to an NMR tube. Catalyst solution (25 µL, 0.00089 mmol) was injected to initiate the reaction and to bring the total volume to 0.7 mL (the substrate was at a concentration of 0.05 M); the sample was transferred to the NMR spectrometer and ^{1}H NMR spectra collected. The time from injection to spectrum #1 was approximately 5 minutes.

Notebook	Solvent	Additive	Amount	Conc./M	k_{obs} x 10^5
3087	CH_2Cl_2	1-PrOH	5 µL	0.095	58.2 ± 1.2
3089			25 µL	0.48	112.3 ± 4.6
3091			55 µL	1.05	184 ± 33
3093	CH_2Cl_2	2-PrOH (i-PrOH)	5 µL	0.094	57.9 ± 1.9
3095			25 µL	0.47	133.1 ± 5.8
3097			55 µL	1.03	270.5 ± 9.7
3099	CH_2Cl_2	CH_3CH_2OH (ethanol)	5 µL	0.12	63.5 ± 2.0
3101			25 µL	0.61	166.4 ± 9.2
3103			55 µL	1.4	353.0 ± 12.6
3107	CH_2Cl_2	1,1,1,3,3,3-hexafluoro-isopropanol (HFIP)	5 µL	0.068	12.6 ± 0.1
3109			25 µL	0.34	1.438 ± 0.009
3111			55 µL	0.75	0.95 ± 0.04
3113	CH_2Cl_2	acetic acid	5 µL	0.12	16.4 ± 0.3
3115			25 µL	0.62	13.4 ± 0.2
3117			55 µL	1.4	12.22 ± 0.07
3121	CH_2Cl_2	DMSO	5 µL	0.1	57 ± 2
3123			25 µL	0.5	66 ± 2
3125			55 µL	1.1	51 ± 1
3131	CH_2Cl_2	1,4-dioxane	5 µL	0.08	40 ± 1
3133			25 µL	0.34	39 ± 1
3135			55 µL	0.75	35.2 ± 0.9
3139	CH_2Cl_2	THF	5 µL	0.9	42 ± 1
3141			25 µL	0.44	43 ± 1
3143			55 µL	0.97	42 ± 1
3153	CH_2Cl_2	acetonitrile	5µ L	0.14	8.28 ± 0.08
3149			25 µL	0.69	4.76 ± 0.02
3151			55 µL	1.5	2.34 ± 0.09

Order in Catalyst (Figure 8 in Paper)

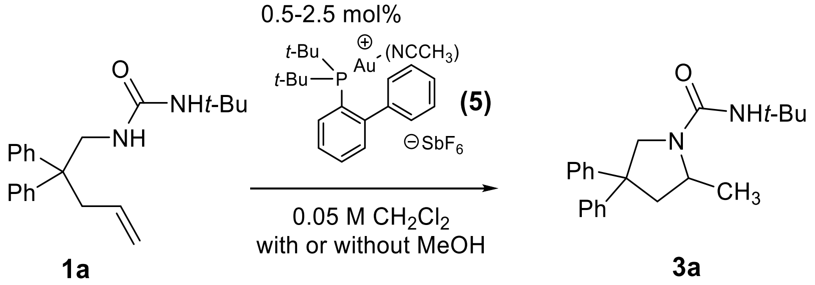

Procedure with 10 uL MeOH. A stock solution of catalyst (0.018 M) was prepared by dissolving JPhosAu($NCCH_3$)SbF_6 (772.17 g/mol, 68.9 mg, 0.0892 mmol) in 5.0 mL of CH_2Cl_2. Urea Alkene (336.48 g/mol, 12 mg, 0.036 mmol) was dissolved in 0.64 mL or 0.67 mL or 0.68 mL of CH_2Cl_2 with 10 µL of CH_3OH and the solution transferred to an NMR tube. Variable amounts of catalyst stock solution were added to initiate the reaction and bring the total volume to 0.7 mL (substrate concentration is 0.05 M). The sample was immediately transferred to the NMR spectrometer and ^{1}H NMR spectra collected. The time from injection to spectrum #1 was approximately 5 minutes. Pseudo first order plots were linear up to at least 80%.

	Catalyst Solution	Catalyst Amount	Catalyst Loading	$10^5 \times k_{obs}$/s^{-1}
BRL 2101	0.01 mL	0.00018 mmol	0.5 mol %	36.9 ± 0.7
BRL 2103	0.02 mL	0.00036 mmol	1 mol %	71 ± 2
BRL 2105	0.05 mL	0.0009 mmol	2.5 mol %	125 ± 7

Procedure without MeOH. A stock solution of catalyst (0.018 M) was prepared by dissolving JPhosAu($NCCH_3$)SbF_6 (772.17 g/mol, 68.9 mg, 0.0892 mmol) in 5.0 mL of CH_2Cl_2. Urea Alkene (336.48 g/mol, 12 mg, 0.036 mmol) was dissolved in 0.69 mL or 0.68 mL or 0.65 mL of CH_2Cl_2 and transferred to an NMR tube. Variable amounts of catalyst stock solution were added to initiate the reaction and bring the total volume to 0.7 mL (substrate concentration is 0.05 M). The sample was transferred to the NMR spectrometer and ^{1}H NMR spectra collected. The time from injection to spectrum #1 was approximately 5 minutes. Pseudo first order plots were linear up to at least 50%.

	Catalyst Solution	Catalyst Amount	Catalyst Loading	$10^5 \times k_{obs}$/s^{-1}
BRL 2107	0.01 mL	0.00018 mmol	0.5 mol %	6.39 ± 0.04
BRL 2109	0.02 mL	0.00036 mmol	1 mol %	12.9 ± 0.2
BRL 2111	0.05 mL	0.0009 mmol	2.5 mol %	29 ± 0.6

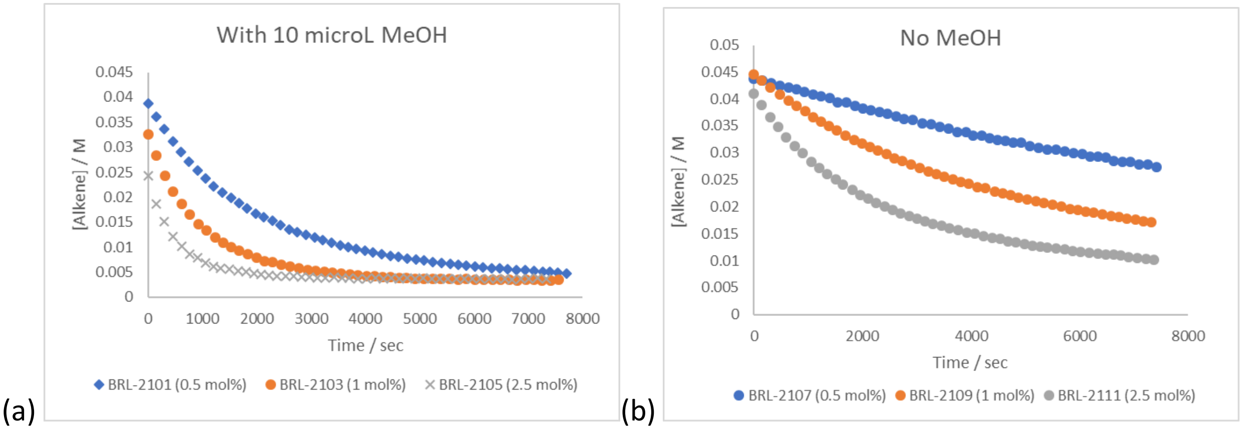

Figure S33. Raw plots showing urea alkene decay (0.05 M) in (a) 0.69 mL CH_2Cl_2/0.01 mL MeOH or (b) 0.7 mL CH_2Cl_2 with variable amounts of [JPhosAu(CNCH$_3$)]Sbf$_6$.

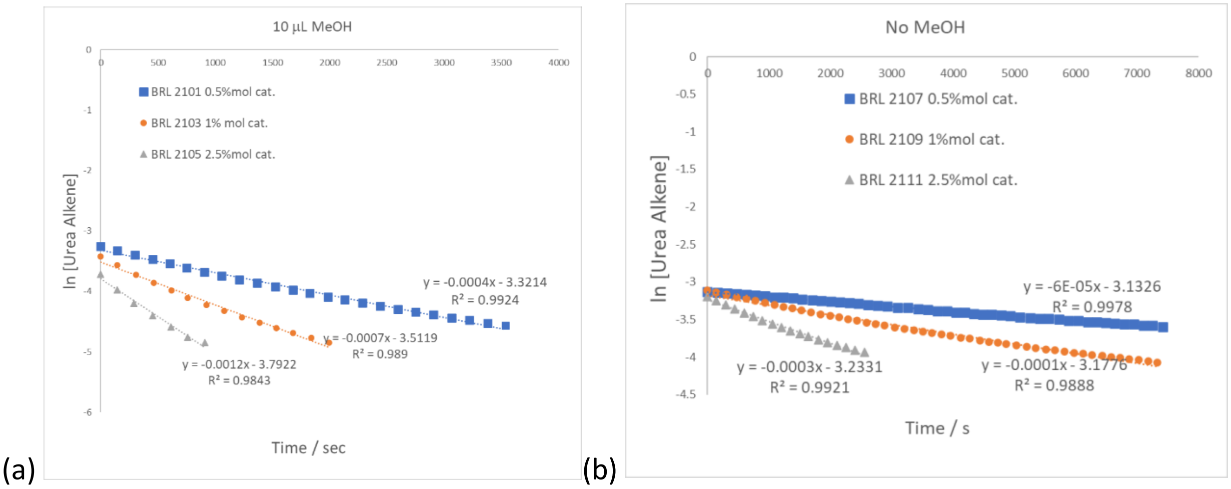

Figure S34. First order plots of urea alkene decay (0.05 M) in (a) 0.69 mL CH_2Cl_2/0.01 mL MeOH or (b) 0.7 mL CH_2Cl_2 with variable amounts of [JPhosAu(CNCH$_3$)]Sbf$_6$.

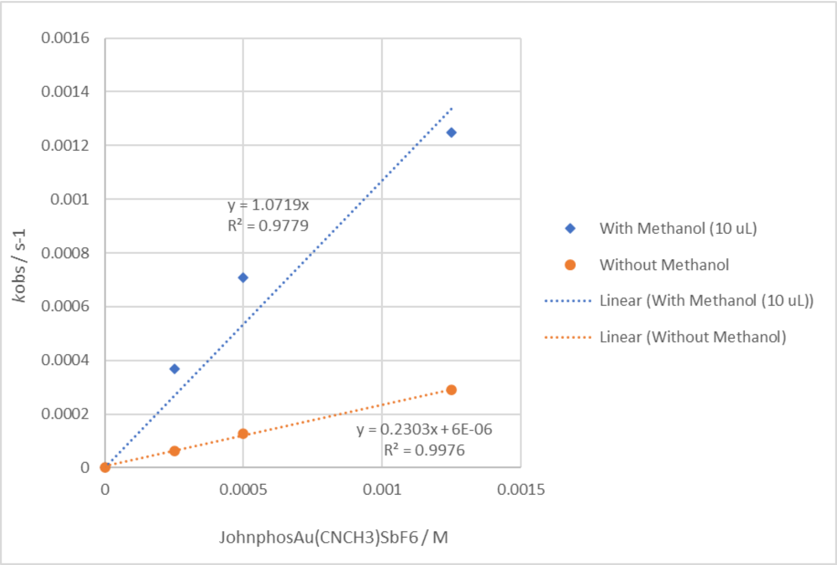

Figure S35. Plots of k_{obs} versus catalyst **5** concentration (0.5 mol %, 1 mol %, 2.5 mol % [JPhosAu($CNCH_3$)]SbF_6) for the urea alkene **1a** cyclization (0.05 M) in CH_2Cl_2 with or without 10 μL methanol additive (total volume 0.7 mL).

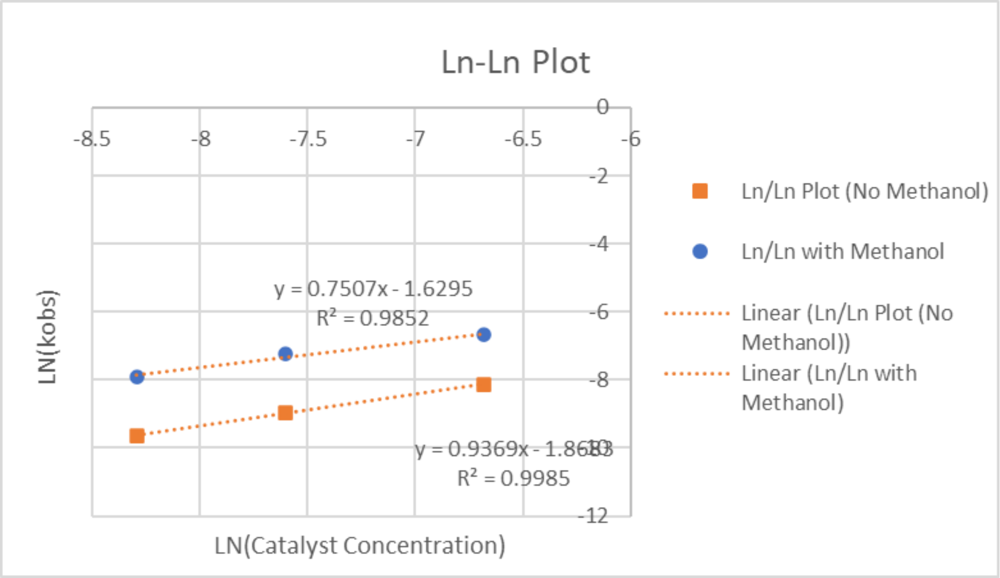

Figure S36. Plots of LN(kobs) versus LN(catalyst concentration) at 0.5 mol %, 1 mol %, 2.5 mol % [JPhosAu($CNCH_3$)]SbF_6 for the urea alkene **1a** cyclization (0.05 M) in CH_2Cl_2 with or without 10 μL methanol additive (total volume 0.7 mL).

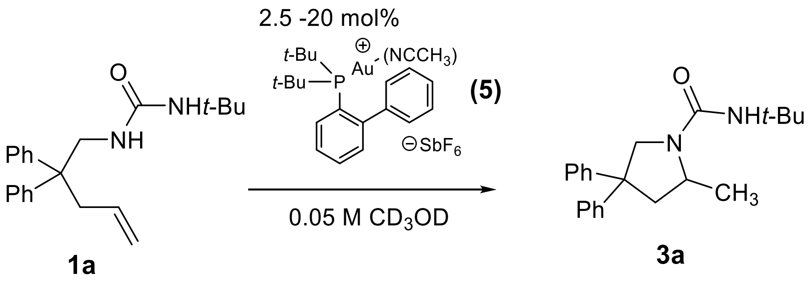

Order in Catalyst in CD_3OD

A similar procedure to the previous section was followed.

	$10^5 \times k_{obs}/s^{-1}$
BRL-4035	4.42 ± 0.06
BRL-6063	21.5 ± 0.6
BRL-6065	36 ± 1

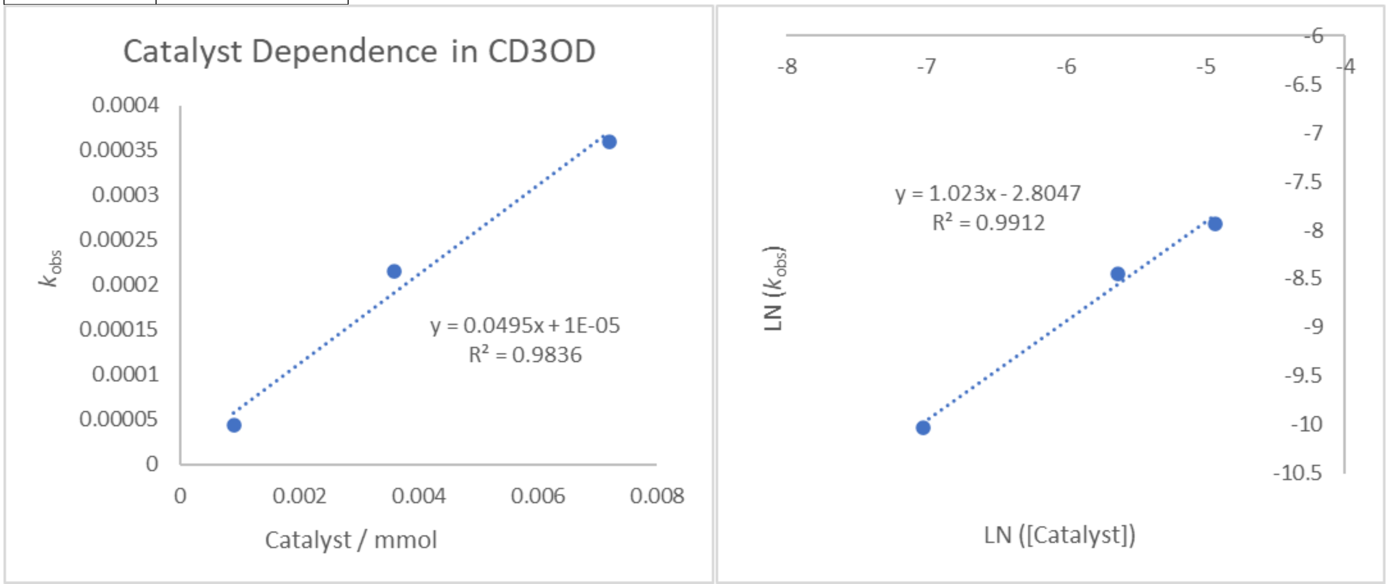

Figure S37. Plots of k_{obs} versus catalyst concentration (2.5 mol %, 10 mol %, 20 mol %) [JPhosAu($CNCH_3$)]SbF_6) for the urea alkene cyclization (0.05 M **1a**) in CD_3OD and corresponding plot of LN(kobs) versus LN(catalyst concentration).

Order in Urea

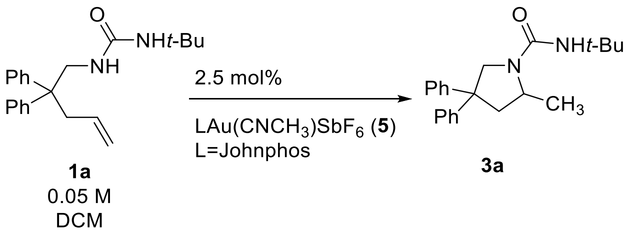

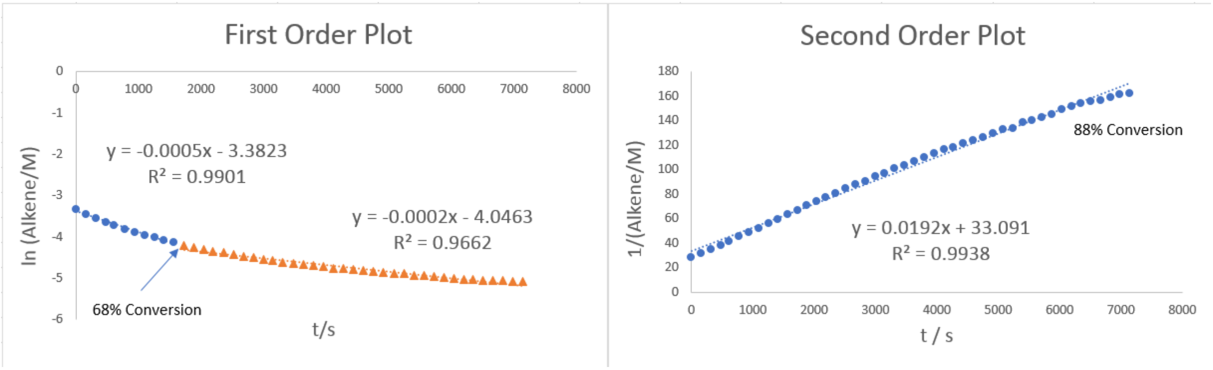

Figure S38. First and second order plots of urea alkene decay (0.05 M) in 0.7 mL of CH_2Cl_2 with [JPhosAu($NCCH_3$)]SbF_6 (0.0009 mmol, 2.5 mol %).

Variable Urea Concentration in $CDCl_3$: The t-butyl urea (0.005 g, 0.01 g, 0.03 g, 0.06 g) was dissolved in $CDCl_3$ (0.5 mL) with 1.5 μL 1,1,2,2-Tetrachloroethane. In a separate vial, the catalyst (0.0039 g, 0.005 mmol) was dissolved in $CDCl_3$ (0.1 mL). A ^{1}H NMR spectrum was collected to check the concentration of alkene by comparison to the TCE standard. The catalyst solution was added to the NMR tube and spectra collected immediately. Plots of Ln(Alkene) were linear up to 80% conversion. A plot of Ln(Alkene) versus Ln(kobs) gives a slope of 0.55.

	**1a** / M	**$10^5 \times k_{obs}$/s^{-1}**
YSJ1133	0.0105 M	42 ± 1
YSJ1137	0.016 M	65 ± 2
YSJ1141	0.054 M	105 ± 3
YSJ1145	0.075 M	139 ± 3

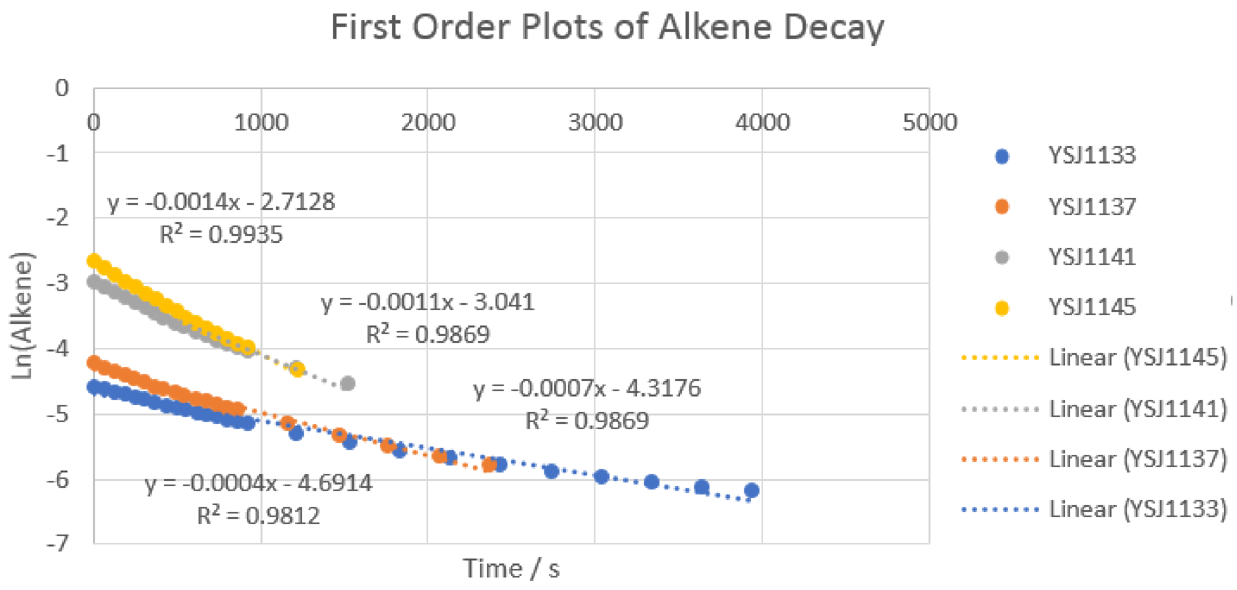

Figure S39. First order plots of urea alkene (**1a**) decay in $CDCl_3$ at various concentrations of **1a**.

Variable Urea Concentration in CH_2Cl_2. A stock solution of catalyst (0.036 M) was prepared by dissolving JPhosAu($NCCH_3$)SbF_6 (772.17 g/mol, 68.9 mg, 0.0892 mmol) in 2.5 mL of CH_2Cl_2. Variable amount of Urea Alkene (336.48 g/mol, 6 mg, 6 mg, 12 mg, 36 mg) was dissolved in 0.675 mL of CH_2Cl_2, with 1.5 µL of 1,1,2,2-tetrachloroethane infused as the internal standard, and then the solution was transferred to an NMR tube. Catalyst solution (25 µL, 0.000892 mmol) was injected to initiate the reaction and to bring the total volume to 0.7 mL (substrate was at a concentration of 0.05 M). The sample was immediately transferred to the NMR spectrometer and ^{1}H NMR spectra collected. The time from injection to spectrum #1 was approximately 5 minutes. Pseudo first order plots were linear up to at least 48% conversion. Concentrations calculated relative to TCE.

	**1a** / M	$10^5 \times k_{obs}/s^{-1}$
BRL-5023	0.036	49 ± 1
BRL-5049	0.038	31.1 ± 0.6
BRL-5047	0.056	37 ± 1
BRL-5025	0.15	33.1 ± 0.9

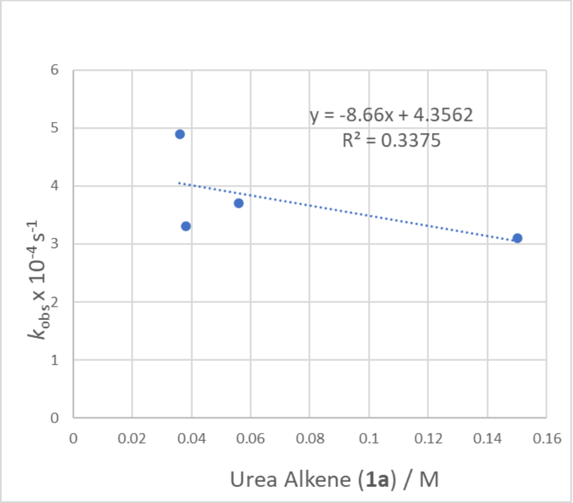

Figure S40. First order rate constants for urea alkene (**1a**) decay in (CH_2Cl_2) showing non-linear dependence on substrate concentration.

Order in Carbamate (Figure 7 in Paper)

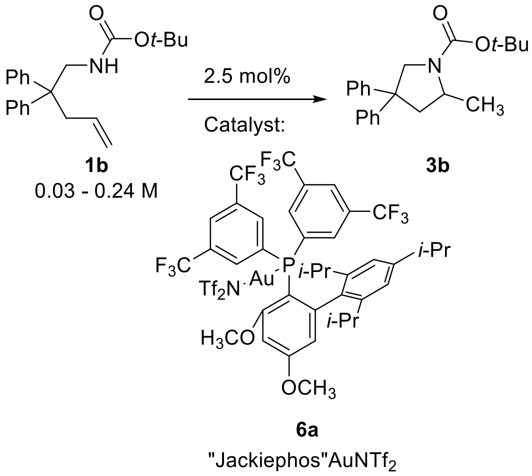

A stock solution of catalyst (0.022 M) was prepared by dissolving Jackiephos-Au-($NTf)_2$ (1273.78 g/mol, 56.7 mg, 0.045 mmol) in 2 mL of CH_2Cl_2. Alkene carbamate (337.46 g/mol), ranging in mass, was dissolved in 0.660 mL of CH_2Cl_2 and transferred to an NMR tube. Catalyst solution (40 µL, 0.000 89mmol) was injected to initiate the reaction and to bring the total volume to 0.7 mL; the sample was transferred to

the NMR spectrometer and ^{1}H NMR spectra collected. The time from injection to spectrum #1 was approximately 5 minutes. Pseudo first order plots maintained their linearity until the end of acquisition of data (80% conversion at the higher concentrations of carbamate).

	Alkene carbamate		Catalyst	Catalyst	Catalyst	$10^5 \times k_{obs}/s^{-1}$
	Mass(mg)	C(M)	Solution	Amount	Loading	
BRL 5051	12	0.05	0.040mL	0.00089 mmol	2.5 mol %	9.73 ± 0.02
BRL 5053	6	0.03				6.96 ± 0.02
BRL 5055	36	0.14				29.5 ± 0.1
BRL 5057	72	0.24				19.6 ± 0.1

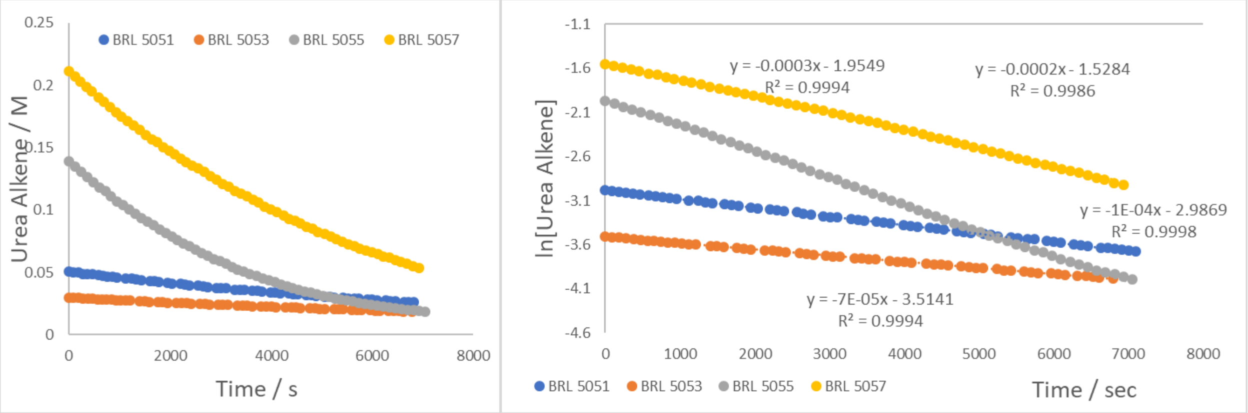

Figure S41. Raw plots of alkene carbamate decay at variable concentrations in 0.7 mL of CH_2Cl_2 with 0.00089 mmol of Jackiephos-Au-$(NTf)_2$ catalyst and the corresponding first order plots.

Activation Parameters

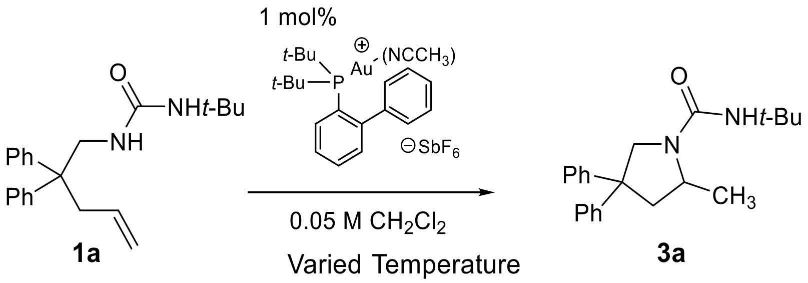

A stock solution of catalyst (0.036 M) was prepared by dissolving $JPhosAu(NCCH_3)SbF_6$ (772.17 g/mol, 68.9 mg, 0.0892 mmol) in 2.5 mL of CH_2Cl_2. Urea alkene (336.48 g/mol, 12 mg, 0.036 mmol) was dissolved 0.690 mL of CH_2Cl_2 and transferred to an NMR tube. If the necessary temperature of a reaction was lower than 20° C, the NMR sample was pre-cooled in a chiller set to the target temperature. Once the spectrometer temperature and chiller reached the desired temperature (after a 0.5-1 hr pre-cooling period), the catalyst solution (10µL, 0.00036mmol) was injected to the NMR tube to bring the total volume to 0.7mL (the substrate was at a concentration of 0.05M); the sample tube was immediately transferred to the NMR spectrometer and ^{1}H NMR spectra collected. If the reaction ought to be conducted at or over 20° C, the catalyst solution was injected directly into a room temperature NMR sample and transferred instantly to the spectrometer, pre-set to the desired temperature. The time from injection to spectrum #1 was

approximately 5 minutes, during which time temperature fluctuations were expected. The spectrometer also re-established the set temperature within that time. In the absence of MeOH, hydroamination was shown to be first order in catalyst. Thus, second order rate constants were calculated by dividing the observed rate constant by the catalyst concentration.

T/°C	T/K	$k_{obs}(s^{-1}) \times 10^5$	Cat. (M)	k_2 (s^{-1} M^{-1})		ln(k_2/T)	1000/T
-5	267	6.12 ± 0.04	0.00051	0.118	BRL 3021	−7.71	3.745
5	278.1	11.9 ± 0.2	0.00051	0.196	BRL 3023	−7.08	3.596
15	288.1	12.2 ± 0.2	0.00051	0.196	BRL 3017	−7.09	3.471
30	303.1	25.9 ± 0.8	0.00051	0.588	BRL 3015	−6.39	3.299

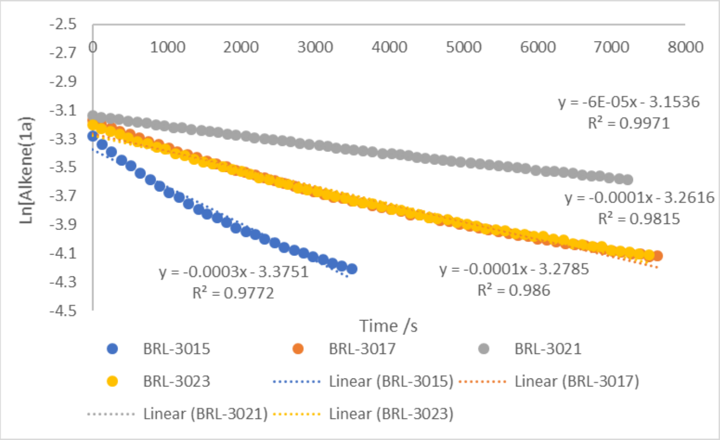

Figure S42. First order plots of urea alkene decay (0.05 M) in 0.7 mL of CH_2Cl_2 and with 0.00036 mmol of [JPhosAu($NCCH_3$)]SbF_6 (1 mol %) at variable temperatures.

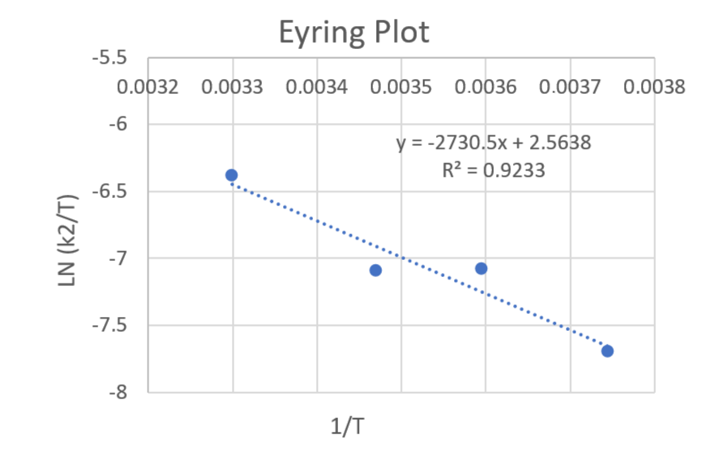

ΔH‡ = -(slope)R

ΔS‡ = R(intercept-k_B/h)

R=1.987 cal K^{-1} mol^{-1}

k_B/h = 23.760

ΔH‡ = 5.4±1.1 kcal/mol

ΔS‡ = -42.1±3.9 eu

Figure S43. The plot of LN(kobs) versus 1/T for urea alkene decay (0.05 M) in 0.7 mL of CH_2Cl_2 and with 0.00036 mmol of [JPhosAu($NCCH_3$)]SbF_6 (1 mol %)

References

S1. LaLonde, R. L.; Brenzovich, Jr., W. E.; Benitez, D.; Tkatchouk, E.; Kelley, K.; Goddard, III, W. A.; Toste, F. D. *Chem. Sci.* **2010**, *1*, 226–233.

S2. Michon, C., Abadie, M.-A., Medina, F., Agbossou-Niedercorn, F. *Catalysis Today*, **2014,** *235*, 2–13.

S3. Mizar, P.; Burrelli, A.; Günther, E.; Söftje, M.; Farooq, U.; Wirth, T. *Chem. Eur. J.* **2014**, *20*, 13113-13116.

S4. Black, D. St. C.; Doyle, J. E. *Aust. J. Chem.* **1978**, *31*, 2247–2257.

S5. Rist, P. A.; Grainger, R. S., Davies P. W. *Org. Lett.* **2021**, *23*, 642–646.

S6. Henrion, G.; Chavas, T. E. J.; Le Goff, X.; Gagosz, F. *Angew. Chem. Int. Ed.* **2013**, *52*, 6277–6282.

S7. Ochida, A.; Ito, H.; Sawamura, M. *J. Am. Chem. Soc.* **2006**, *128*, 16486–16487.
