# Supplementary material for: Ligand effects, solvent cooperation, and large kinetic solvent deuterium isotope effects in gold(I)-catalyzed intramolecular alkene hydroamination
Source: Beilstein J Org Chem. 2024 Feb 29;20:479–96. doi: 10.3762/bjoc.20.43 (PMC10910400; doi:10.3762/bjoc.20.43)
Supplement: File 2 — Crystallographic experimental details. [file Beilstein_J_Org_Chem-20-479-s002.pdf]

BEILSTEIN JOURNAL OF ORGANIC CHEMISTRY

Supporting Information

for

Ligand effects, solvent cooperation, and large kinetic solvent deuterium isotope effects in gold(I)-catalyzed intramolecular alkene hydroamination

Ruichen Lan, Brock Yager, Yoonsun Jee, Cynthia S. Day and Amanda C. Jones

Beilstein J. Org. Chem. **2024,** *20,* 479–496. doi:10.3762/bjoc.20.43

Crystallographic experimental details

License and Terms: This is a supporting information file under the terms of the Creative Commons Attribution License (https://creativecommons.org/licenses/by/4.0). Please note that the reuse, redistribution and reproduction in particular requires that the author(s) and source are credited and that individual graphics may be subject to special legal provisions.
The license is subject to the *Beilstein Journal of Organic Chemistry* terms and conditions: (https://www.beilstein-journals.org/bjoc/terms)

Crystallographic Experimental Details

A specimen of $C_{41}H_{56}AuCl_2F_6P_2Sb$, approximate dimensions 0.184 mm x 0.199 mm x 0.309 mm, was used for the X-ray crystallographic analysis. The X-ray intensity data were measured on a Bruker APEX CCD system equipped with a graphite monochromator and a MoKα sealed x-ray tube (λ = 0.71073 Å).

The frames were integrated with the Bruker SAINT software package using a narrow-frame algorithm. The integration of the data using a monoclinic unit cell yielded a total of 41056 reflections to a maximum θ angle of 30.09° (0.71 Å resolution), of which 12850 were independent (average redundancy 3.195, completeness = 99.8%, R_{int} = 3.58%, R_{sig} = 3.45%) and 12250 (95.33%) were greater than $2\sigma(F^2)$. The final cell constants of a = 13.9857 (9) Å, b = 10.9855 (7) Å, c = 15.0464 (10) Å, β = 108.222 (1)°, volume = 2195.8 (2) $Å^3$, are based upon the refinement of the XYZ-centroids of reflections above 20 σ(I). Data were corrected for absorption effects using the multi-scan method (SADABS). The ratio of minimum to maximum apparent transmission was 0.796. The calculated minimum and maximum transmission coefficients (based on crystal size) are 0.3570 and 0.5120.

The structure was solved and refined using the Bruker SHELXTL Software Package within the APEX3 software suite, using the space group P n, with Z = 2 for the formula unit, $C_{41}H_{56}AuCl_2F_6P_2Sb$. The final anisotropic full-matrix least-squares refinement on F^2 with 490 variables converged at R_1 = 2.56%, for the observed data and wR_2 = 6.35% for all data. The goodness-of-fit was 1.027. The largest peak in the final difference electron density synthesis was 1.533 e-/Å3 and the largest hole was -1.603 e-/Å3 with an RMS deviation of 0.082 e-/Å3. On the basis of the final model, the calculated density was 1.686 g/cm3 and F(000), 1100 e-.

Crystal data, data collection and structure refinement details are summarized in tables below.

Computing details

Data collection: *APEX3* (Bruker, 2017); cell refinement: *SAINT* (Bruker, 2014); data reduction: *SAINT* (Bruker, 2014); absorption correction: *SADABS* (Krause, 2015); program(s) used to solve structure: *SHELXT* (Sheldrick, 2015a); program(s) used to refine structure: *SHELXL* (Sheldrick, 2015b); molecular graphics: *XP* in *SHELXTL* (Sheldrick, 2008); software used to prepare material for publication: *SHELXL* (Sheldrick, 2015b) and *publCIF* (Westrip, 2010).

Acknowledgements

The WFU X-ray Facility thanks the National Science Foundation (grant CHE-0234489) for funds to purchase the X-ray instrument and computers.

References

Bruker (2017). *APEX3* (Version 2017.3). Bruker AXS Inc., Madison, WI, USA.

Bruker (2014). *SAINT* version 8.34A. Bruker AXS Inc., Madison, WI, USA.

Krause, L., Herbst-Irmer, R., Sheldrick, G. M. & Stalke, D. (2015). *J. Appl. Cryst.* **48**, 3–10.

Sheldrick, G. M. (2008). *Acta Cryst.* A**64**, 112–122.

Sheldrick, G. M. (2016). *SHELXL* (Version 2016/6). Bruker AXS Inc., Madison, Wisconsin, USA.

Sheldrick, G. M. (2015*a*). *Acta Cryst.* A**71**, 3–8.

Sheldrick, G. M. (2015*b*). *Acta Cryst.* C**71**, 3–8.

Westrip, S. P. (2010). *J. Appl. Cryst.* **43**, 920–925.

Crystal data

$C_{41}H_{56}AuCl_2F_6P_2Sb$	$F(000) = 1100$
$M_r = 1114.41$	$D_x = 1.686$ Mg m^{-3}
Monoclinic, *Pn*	Mo $K\alpha$ radiation, $\lambda = 0.71073$ Å
$a = 13.9857$ (9) Å	Cell parameters from 9780 reflections
$b = 10.9855$ (7) Å	$\theta = 3.5–30.0°$
$c = 15.0464$ (10) Å	$\mu = 4.20$ mm^{-1}
$\beta = 108.222$ (1)°	$T = 194$ K
$V = 2195.8$ (2) Å^3	Block, colourless
$Z = 2$	$0.31 \times 0.20 \times 0.18$ mm

Data collection

Bruker APEX CCD diffractometer	12850 independent reflections
Radiation source: sealed x-ray tube	12250 reflections with $I > 2\sigma(I)$
Graphite monochromator	$R_{int} = 0.036$
ϕ and ω scans	$\theta_{max} = 30.1°$, $\theta_{min} = 3.5°$
Absorption correction: multi-scan<br>Data were corrected for scaling and absorption effects using the multi-scan technique (*SADABS*). The ratio of minimum to maximum apparent transmission was 0.796. The calculated minimum and maximum transmission coefficients (based on crystal size) are 0.357 and 0.512.<br>(*SADABS*; Krause *et al.*, 2015)	$h = -19 \rightarrow 19$
$T_{min} = 0.594$, $T_{max} = 0.746$	$k = -15 \rightarrow 15$
41056 measured reflections	$l = -21 \rightarrow 21$

Refinement

Refinement on F^2	Secondary atom site location: difference Fourier map
Least-squares matrix: full	Hydrogen site location: inferred from neighbouring sites
$R[F^2 > 2\sigma(F^2)] = 0.026$	H-atom parameters constrained
$wR(F^2) = 0.064$	$w = 1/[\sigma^2(F_o^2) + (0.0343P)^2 + 1.0791P]$<br>where $P = (F_o^2 + 2F_c^2)/3$
$S = 1.03$	$(\Delta/\sigma)_{max} = 0.001$
12850 reflections	$\Delta\rangle_{max} = 1.53$ e Å^{-3}

490 parameters	$\Delta\rangle_{min}$ = -1.60 e Å^{-3}
2 restraints	Absolute structure: Flack x determined using 5795 quotients [(I+)-(I-)]/[(I+)+(I-)] (Parsons, Flack and Wagner, Acta Cryst. B69 (2013) 249-259).<br>Flack -0.010(3); Number Bijvoet Pairs = 6392 (99%), 5848 Selected for: Parsons -0.010(2) # P2(tr) 1.000, P3(tr) 1.000, P3(tw) 0.000, Student-T Nu = 28, Hooft -0.012(1)
Primary atom site location: structure-invariant direct methods	Absolute structure parameter: -0.010 (3)

Special details

Geometry. All esds (except the esd in the dihedral angle between two l.s. planes) are estimated using the full covariance matrix. The cell esds are taken into account individually in the estimation of esds in distances, angles and torsion angles; correlations between esds in cell parameters are only used when they are defined by crystal symmetry. An approximate (isotropic) treatment of cell esds is used for estimating esds involving l.s. planes.

Fractional atomic coordinates and isotropic or equivalent isotropic displacement parameters (Å^2)

	x	*y*	*z*	U_{iso}*/U_{eq}
Au1	0.21164 (2)	0.35458 (2)	0.47770 (2)	0.02118 (4)
P1	0.30025 (8)	0.43939 (10)	0.38145 (8)	0.0210 (2)
P2	0.17544 (9)	0.25062 (11)	0.60147 (8)	0.0219 (2)
C1	0.2711 (4)	0.5923 (4)	0.3312 (4)	0.0277 (9)
C2	0.2140 (5)	0.6799 (5)	0.3598 (5)	0.0375 (12)
C3	0.2090 (7)	0.7985 (6)	0.3222 (7)	0.065 (2)
H3	0.172530	0.859381	0.342785	0.078*
C4	0.2550 (8)	0.8284 (7)	0.2571 (8)	0.069 (3)
H4	0.249339	0.908764	0.232732	0.083*
C5	0.3086 (6)	0.7437 (6)	0.2274 (6)	0.0505 (16)
H5	0.339539	0.763388	0.181350	0.061*
C6	0.3174 (5)	0.6278 (5)	0.2656 (5)	0.0387 (12)
H6	0.356976	0.569650	0.246022	0.046*
C7	0.1510 (5)	0.6620 (5)	0.4222 (5)	0.0403 (13)
C8	0.0640 (5)	0.5923 (6)	0.3934 (6)	0.0472 (15)
H8	0.053022	0.540620	0.340447	0.057*
C9	-0.0074 (7)	0.5961 (8)	0.4400 (8)	0.066 (2)
H9	-0.067027	0.548746	0.418669	0.079*
C10	0.0095 (9)	0.6699 (9)	0.5178 (9)	0.079 (3)

H10	-0.039545	0.675045	0.549306	0.094*
C11	0.0960 (11)	0.7349 (9)	0.5493 (8)	0.085 (3)
H11	0.108058	0.782587	0.604391	0.102*
C12	0.1675 (7)	0.7328 (6)	0.5023 (6)	0.061 (2)
H12	0.227331	0.779663	0.524903	0.073*
C13	0.2855 (4)	0.3330 (5)	0.2792 (4)	0.0271 (10)
C14	0.3725 (5)	0.3251 (6)	0.2370 (5)	0.0391 (12)
H14A	0.352994	0.271723	0.182076	0.059*
H14B	0.432396	0.291971	0.283764	0.059*
H14C	0.387493	0.406579	0.218378	0.059*
C15	0.2694 (5)	0.2042 (5)	0.3104 (4)	0.0344 (11)
H15A	0.258618	0.148014	0.257608	0.052*
H15B	0.210284	0.203269	0.332237	0.052*
H15C	0.328894	0.178832	0.361439	0.052*
C16	0.1884 (5)	0.3745 (6)	0.2033 (4)	0.0374 (12)
H16A	0.166475	0.311056	0.155439	0.056*
H16B	0.201612	0.449893	0.174316	0.056*
H16C	0.135391	0.388967	0.231981	0.056*
C17	0.4341 (3)	0.4542 (4)	0.4589 (4)	0.0269 (9)
C18	0.5068 (4)	0.5183 (6)	0.4155 (5)	0.0385 (12)
H18A	0.574758	0.518538	0.460572	0.058*
H18B	0.484471	0.602363	0.399509	0.058*
H18C	0.507366	0.474915	0.358778	0.058*
C19	0.4295 (4)	0.5312 (6)	0.5433 (4)	0.0414 (13)
H19A	0.496946	0.536824	0.589061	0.062*
H19B	0.383662	0.492654	0.572532	0.062*
H19C	0.405059	0.613096	0.521981	0.062*
C20	0.4760 (4)	0.3278 (6)	0.4922 (4)	0.0360 (11)
H20A	0.540020	0.336002	0.542629	0.054*
H20B	0.486947	0.282972	0.439923	0.054*
H20C	0.427849	0.283393	0.515428	0.054*
C21	0.0632 (4)	0.1549 (4)	0.5815 (3)	0.0236 (8)
C22	0.0036 (3)	0.1114 (4)	0.4936 (3)	0.0232 (8)
C23	-0.0718 (4)	0.0247 (5)	0.4891 (4)	0.0320 (10)
H23	-0.110803	-0.006125	0.429903	0.038*
C24	-0.0907 (4)	-0.0166 (5)	0.5688 (4)	0.0342 (11)
H24	-0.142162	-0.074991	0.563973	0.041*

C25	-0.0347 (4)	0.0269 (5)	0.6554 (4)	0.0333 (10)
H25	-0.047869	-0.000056	0.710400	0.040*
C26	0.0416 (4)	0.1112 (5)	0.6611 (3)	0.0303 (10)
H26	0.080537	0.140111	0.720966	0.036*
C27	0.0092 (4)	0.1517 (4)	0.4008 (3)	0.0242 (9)
C28	0.0340 (4)	0.0689 (5)	0.3417 (4)	0.0316 (10)
H28	0.059264	-0.008916	0.365096	0.038*
C29	0.0227 (4)	0.0977 (6)	0.2493 (4)	0.0361 (11)
H29	0.042307	0.041044	0.210561	0.043*
C30	-0.0171 (5)	0.2091 (6)	0.2139 (4)	0.0390 (13)
H30	-0.028408	0.227752	0.149818	0.047*
C31	-0.0399 (5)	0.2917 (6)	0.2712 (5)	0.0471 (15)
H31	-0.065342	0.369162	0.246879	0.056*
C32	-0.0270 (5)	0.2657 (5)	0.3650 (4)	0.0359 (11)
H32	-0.042696	0.325127	0.404153	0.043*
C33	0.1717 (5)	0.3670 (5)	0.6934 (4)	0.0315 (12)
C34	0.2406 (5)	0.4727 (6)	0.6854 (5)	0.0419 (13)
H34A	0.240986	0.534924	0.732204	0.063*
H34B	0.215528	0.508388	0.622668	0.063*
H34C	0.309119	0.442201	0.696233	0.063*
C35	0.0633 (5)	0.4133 (6)	0.6677 (5)	0.0442 (14)
H35A	0.061275	0.487393	0.703303	0.066*
H35B	0.020814	0.350708	0.682748	0.066*
H35C	0.038316	0.431293	0.600588	0.066*
C36	0.2072 (5)	0.3252 (7)	0.7964 (4)	0.0443 (14)
H36A	0.209910	0.395371	0.837294	0.066*
H36B	0.274260	0.288687	0.811079	0.066*
H36C	0.159859	0.264807	0.806233	0.066*
C37	0.2817 (4)	0.1374 (4)	0.6458 (4)	0.0291 (10)
C38	0.3810 (4)	0.2055 (6)	0.6869 (5)	0.0401 (13)
H38A	0.437268	0.148863	0.695600	0.060*
H38B	0.383438	0.240706	0.747461	0.060*
H38C	0.385849	0.270721	0.644106	0.060*
C39	0.2832 (4)	0.0640 (5)	0.5589 (4)	0.0348 (11)
H39A	0.333731	-0.000729	0.577757	0.052*
H39B	0.299992	0.118203	0.514238	0.052*
H39C	0.216717	0.027810	0.529421	0.052*

C40	0.2677 (5)	0.0464 (6)	0.7193 (5)	0.0419 (13)
H40A	0.326644	-0.006904	0.740259	0.063*
H40B	0.207315	-0.002842	0.691294	0.063*
H40C	0.260133	0.091430	0.772982	0.063*
Sb1	0.57627 (4)	-0.06445 (4)	0.46314 (3)	0.04642 (11)
F1	0.6766 (4)	-0.0229 (6)	0.4092 (4)	0.0787 (15)
F2	0.5277 (5)	-0.1752 (5)	0.3660 (4)	0.0697 (14)
F3	0.4920 (6)	0.0509 (6)	0.3880 (5)	0.096 (2)
F4	0.4783 (7)	-0.1150 (10)	0.5135 (6)	0.129 (3)
F5	0.6263 (10)	0.0514 (10)	0.5549 (6)	0.173 (5)
F6	0.6639 (7)	-0.1780 (11)	0.5341 (8)	0.172 (5)
Cl1	0.6733 (3)	0.2696 (3)	0.3297 (2)	0.0899 (8)
Cl2	0.7881 (2)	0.4015 (3)	0.4955 (2)	0.0857 (7)
C41	0.7333 (7)	0.2609 (9)	0.4508 (8)	0.074 (3)
H41A	0.786096	0.197402	0.464112	0.089*
H41B	0.683624	0.237397	0.482284	0.089*

Atomic displacement parameters (Å^2)

	U^{11}	U^{22}	U^{33}	U^{12}	U^{13}	U^{23}
Au1	0.02046 (6)	0.02158 (7)	0.02277 (7)	0.00012 (8)	0.00856 (5)	-0.00092 (7)
P1	0.0208 (5)	0.0190 (5)	0.0252 (5)	-0.0007 (4)	0.0098 (4)	-0.0020 (4)
P2	0.0212 (5)	0.0246 (5)	0.0205 (5)	-0.0017 (4)	0.0073 (4)	-0.0015 (4)
C1	0.027 (2)	0.020 (2)	0.035 (2)	0.0001 (17)	0.0094 (19)	0.0042 (18)
C2	0.038 (3)	0.027 (2)	0.053 (3)	0.009 (2)	0.023 (3)	0.008 (2)
C3	0.083 (6)	0.033 (3)	0.097 (6)	0.027 (3)	0.052 (5)	0.023 (4)
C4	0.085 (6)	0.037 (3)	0.101 (7)	0.020 (4)	0.052 (6)	0.035 (4)
C5	0.055 (4)	0.042 (3)	0.061 (4)	-0.003 (3)	0.028 (3)	0.017 (3)
C6	0.044 (3)	0.032 (3)	0.046 (3)	0.002 (2)	0.022 (3)	0.008 (2)
C7	0.045 (3)	0.029 (3)	0.054 (4)	0.014 (2)	0.026 (3)	0.007 (2)
C8	0.039 (3)	0.041 (3)	0.067 (4)	0.014 (3)	0.025 (3)	0.016 (3)
C9	0.059 (5)	0.056 (4)	0.102 (7)	0.023 (4)	0.054 (5)	0.031 (5)
C10	0.097 (8)	0.058 (5)	0.114 (8)	0.038 (5)	0.081 (7)	0.027 (5)
C11	0.141 (10)	0.053 (5)	0.087 (7)	0.031 (6)	0.074 (7)	-0.003 (5)
C12	0.089 (6)	0.029 (3)	0.078 (5)	0.008 (3)	0.047 (5)	0.001 (3)
C13	0.029 (3)	0.029 (2)	0.025 (2)	-0.003 (2)	0.012 (2)	-0.0062 (19)
C14	0.041 (3)	0.038 (3)	0.048 (3)	-0.006 (2)	0.028 (3)	-0.011 (2)

C15	0.044 (3)	0.024 (2)	0.040 (3)	-0.010 (2)	0.021 (2)	-0.012 (2)
C16	0.037 (3)	0.044 (3)	0.027 (2)	-0.006 (2)	0.005 (2)	-0.005 (2)
C17	0.0209 (19)	0.028 (2)	0.031 (2)	-0.0026 (17)	0.0083 (17)	-0.0049 (18)
C18	0.028 (2)	0.042 (3)	0.049 (3)	-0.009 (2)	0.017 (2)	-0.003 (2)
C19	0.030 (3)	0.047 (3)	0.041 (3)	-0.007 (2)	0.003 (2)	-0.017 (3)
C20	0.029 (2)	0.038 (3)	0.041 (3)	0.000 (2)	0.010 (2)	-0.001 (2)
C21	0.022 (2)	0.025 (2)	0.026 (2)	-0.0004 (15)	0.0097 (17)	-0.0005 (16)
C22	0.0229 (19)	0.025 (2)	0.0216 (19)	0.0014 (16)	0.0068 (16)	-0.0003 (16)
C23	0.032 (2)	0.032 (2)	0.026 (2)	-0.007 (2)	0.002 (2)	-0.0014 (19)
C24	0.031 (2)	0.031 (2)	0.041 (3)	-0.010 (2)	0.011 (2)	-0.001 (2)
C25	0.036 (3)	0.038 (3)	0.030 (2)	-0.003 (2)	0.017 (2)	0.004 (2)
C26	0.032 (2)	0.038 (2)	0.023 (2)	-0.006 (2)	0.0107 (19)	-0.0019 (19)
C27	0.0203 (19)	0.029 (2)	0.022 (2)	-0.0018 (16)	0.0041 (16)	0.0017 (16)
C28	0.034 (2)	0.034 (2)	0.027 (2)	0.000 (2)	0.0104 (19)	-0.0013 (19)
C29	0.040 (3)	0.043 (3)	0.029 (2)	-0.014 (2)	0.015 (2)	-0.009 (2)
C30	0.040 (3)	0.051 (3)	0.023 (2)	-0.015 (3)	0.004 (2)	0.002 (2)
C31	0.056 (4)	0.043 (3)	0.038 (3)	0.008 (3)	0.009 (3)	0.016 (3)
C32	0.044 (3)	0.031 (3)	0.034 (3)	0.008 (2)	0.014 (2)	0.005 (2)
C33	0.035 (3)	0.034 (3)	0.030 (3)	-0.008 (2)	0.016 (2)	-0.012 (2)
C34	0.054 (3)	0.036 (3)	0.045 (3)	-0.014 (3)	0.029 (3)	-0.017 (2)
C35	0.048 (3)	0.040 (3)	0.053 (4)	0.002 (3)	0.029 (3)	-0.014 (3)
C36	0.050 (3)	0.058 (4)	0.026 (2)	-0.016 (3)	0.014 (2)	-0.011 (2)
C37	0.026 (2)	0.030 (2)	0.029 (2)	0.0051 (17)	0.0057 (19)	0.0083 (18)
C38	0.027 (2)	0.046 (3)	0.042 (3)	-0.001 (2)	0.004 (2)	0.004 (2)
C39	0.035 (3)	0.031 (3)	0.041 (3)	0.010 (2)	0.014 (2)	0.002 (2)
C40	0.037 (3)	0.047 (3)	0.040 (3)	0.007 (2)	0.010 (2)	0.018 (3)
Sb1	0.0578 (3)	0.0511 (2)	0.02958 (18)	-0.0051 (2)	0.01254 (17)	0.00285 (17)
F1	0.064 (3)	0.092 (4)	0.088 (4)	-0.023 (3)	0.034 (3)	0.010 (3)
F2	0.094 (4)	0.056 (3)	0.070 (3)	-0.024 (3)	0.040 (3)	-0.021 (2)
F3	0.112 (5)	0.077 (4)	0.103 (5)	0.054 (4)	0.039 (4)	0.018 (3)
F4	0.134 (7)	0.202 (8)	0.088 (5)	-0.035 (6)	0.088 (5)	-0.004 (5)
F5	0.242 (12)	0.193 (11)	0.073 (5)	-0.076 (9)	0.036 (6)	-0.080 (6)
F6	0.117 (7)	0.208 (9)	0.163 (9)	0.029 (6)	0.003 (6)	0.151 (8)
Cl1	0.113 (2)	0.0731 (15)	0.0934 (18)	0.0028 (14)	0.0457 (16)	0.0277 (14)
Cl2	0.0645 (13)	0.0943 (17)	0.0992 (19)	0.0009 (13)	0.0269 (13)	0.0250 (16)
C41	0.061 (5)	0.072 (6)	0.101 (7)	0.024 (4)	0.043 (5)	0.040 (5)

Geometric parameters (Å, º)

Au1—P2	2.3703 (12)	C21—C22	1.408 (6)
Au1—P1	2.3715 (12)	C22—C23	1.408 (7)
P1—C1	1.835 (5)	C22—C27	1.490 (7)
P1—C17	1.878 (5)	C23—C24	1.383 (8)
P1—C13	1.892 (5)	C23—H23	0.9500
P2—C21	1.835 (5)	C24—C25	1.378 (8)
P2—C37	1.892 (5)	C24—H24	0.9500
P2—C33	1.897 (5)	C25—C26	1.394 (7)
C1—C6	1.396 (8)	C25—H25	0.9500
C1—C2	1.401 (7)	C26—H26	0.9500
C2—C3	1.412 (9)	C27—C28	1.389 (7)
C2—C7	1.488 (9)	C27—C32	1.394 (7)
C3—C4	1.370 (12)	C28—C29	1.385 (7)
C3—H3	0.9500	C28—H28	0.9500
C4—C5	1.355 (12)	C29—C30	1.380 (10)
C4—H4	0.9500	C29—H29	0.9500
C5—C6	1.386 (8)	C30—C31	1.357 (10)
C5—H5	0.9500	C30—H30	0.9500
C6—H6	0.9500	C31—C32	1.395 (8)
C7—C8	1.388 (10)	C31—H31	0.9500
C7—C12	1.392 (11)	C32—H32	0.9500
C8—C9	1.389 (10)	C33—C35	1.529 (9)
C8—H8	0.9500	C33—C34	1.537 (8)
C9—C10	1.382 (16)	C33—C36	1.542 (9)
C9—H9	0.9500	C34—H34A	0.9800
C10—C11	1.356 (17)	C34—H34B	0.9800
C10—H10	0.9500	C34—H34C	0.9800
C11—C12	1.394 (13)	C35—H35A	0.9800
C11—H11	0.9500	C35—H35B	0.9800
C12—H12	0.9500	C35—H35C	0.9800
C13—C15	1.530 (8)	C36—H36A	0.9800
C13—C14	1.541 (8)	C36—H36B	0.9800
C13—C16	1.545 (8)	C36—H36C	0.9800
C14—H14A	0.9800	C37—C38	1.528 (8)
C14—H14B	0.9800	C37—C39	1.542 (8)
C14—H14C	0.9800	C37—C40	1.548 (8)

C15—H15A	0.9800	C38—H38A	0.9800
C15—H15B	0.9800	C38—H38B	0.9800
C15—H15C	0.9800	C38—H38C	0.9800
C16—H16A	0.9800	C39—H39A	0.9800
C16—H16B	0.9800	C39—H39B	0.9800
C16—H16C	0.9800	C39—H39C	0.9800
C17—C20	1.529 (8)	C40—H40A	0.9800
C17—C18	1.540 (7)	C40—H40B	0.9800
C17—C19	1.544 (8)	C40—H40C	0.9800
C18—H18A	0.9800	Sb1—F6	1.839 (7)
C18—H18B	0.9800	Sb1—F4	1.846 (6)
C18—H18C	0.9800	Sb1—F5	1.847 (7)
C19—H19A	0.9800	Sb1—F3	1.855 (6)
C19—H19B	0.9800	Sb1—F2	1.859 (5)
C19—H19C	0.9800	Sb1—F1	1.883 (5)
C20—H20A	0.9800	Cl1—C41	1.754 (12)
C20—H20B	0.9800	Cl2—C41	1.761 (12)
C20—H20C	0.9800	C41—H41A	0.9900
C21—C26	1.408 (7)	C41—H41B	0.9900
P2—Au1—P1	161.79 (4)	C23—C22—C27	114.5 (4)
C1—P1—C17	103.1 (2)	C21—C22—C27	126.2 (4)
C1—P1—C13	106.1 (3)	C24—C23—C22	121.6 (5)
C17—P1—C13	113.1 (2)	C24—C23—H23	119.2
C1—P1—Au1	121.34 (17)	C22—C23—H23	119.2
C17—P1—Au1	105.36 (16)	C25—C24—C23	120.0 (5)
C13—P1—Au1	108.01 (17)	C25—C24—H24	120.0
C21—P2—C37	102.6 (2)	C23—C24—H24	120.0
C21—P2—C33	106.8 (2)	C24—C25—C26	119.1 (5)
C37—P2—C33	112.6 (3)	C24—C25—H25	120.5
C21—P2—Au1	121.98 (16)	C26—C25—H25	120.5
C37—P2—Au1	104.87 (17)	C25—C26—C21	122.5 (5)
C33—P2—Au1	108.03 (19)	C25—C26—H26	118.7
C6—C1—C2	117.4 (5)	C21—C26—H26	118.7
C6—C1—P1	117.1 (4)	C28—C27—C32	118.3 (5)
C2—C1—P1	125.2 (4)	C28—C27—C22	120.1 (4)
C1—C2—C3	118.2 (6)	C32—C27—C22	120.6 (5)

C1—C2—C7	127.5 (5)	C29—C28—C27	121.3 (5)
C3—C2—C7	114.2 (5)	C29—C28—H28	119.3
C4—C3—C2	122.1 (7)	C27—C28—H28	119.3
C4—C3—H3	118.9	C30—C29—C28	119.7 (5)
C2—C3—H3	118.9	C30—C29—H29	120.2
C5—C4—C3	120.2 (6)	C28—C29—H29	120.2
C5—C4—H4	119.9	C31—C30—C29	119.7 (5)
C3—C4—H4	119.9	C31—C30—H30	120.2
C4—C5—C6	118.8 (7)	C29—C30—H30	120.2
C4—C5—H5	120.6	C30—C31—C32	121.6 (6)
C6—C5—H5	120.6	C30—C31—H31	119.2
C5—C6—C1	123.3 (6)	C32—C31—H31	119.2
C5—C6—H6	118.4	C27—C32—C31	119.4 (6)
C1—C6—H6	118.4	C27—C32—H32	120.3
C8—C7—C12	118.1 (7)	C31—C32—H32	120.3
C8—C7—C2	120.7 (7)	C35—C33—C34	109.0 (5)
C12—C7—C2	120.1 (7)	C35—C33—C36	109.6 (5)
C7—C8—C9	121.6 (8)	C34—C33—C36	106.9 (5)
C7—C8—H8	119.2	C35—C33—P2	106.9 (4)
C9—C8—H8	119.2	C34—C33—P2	106.9 (4)
C10—C9—C8	119.1 (9)	C36—C33—P2	117.3 (4)
C10—C9—H9	120.4	C33—C34—H34A	109.5
C8—C9—H9	120.4	C33—C34—H34B	109.5
C11—C10—C9	120.2 (8)	H34A—C34—H34B	109.5
C11—C10—H10	119.9	C33—C34—H34C	109.5
C9—C10—H10	119.9	H34A—C34—H34C	109.5
C10—C11—C12	121.1 (9)	H34B—C34—H34C	109.5
C10—C11—H11	119.4	C33—C35—H35A	109.5
C12—C11—H11	119.4	C33—C35—H35B	109.5
C7—C12—C11	119.8 (9)	H35A—C35—H35B	109.5
C7—C12—H12	120.1	C33—C35—H35C	109.5
C11—C12—H12	120.1	H35A—C35—H35C	109.5
C15—C13—C14	106.6 (5)	H35B—C35—H35C	109.5
C15—C13—C16	108.7 (5)	C33—C36—H36A	109.5
C14—C13—C16	109.7 (5)	C33—C36—H36B	109.5
C15—C13—P1	108.1 (4)	H36A—C36—H36B	109.5
C14—C13—P1	118.1 (4)	C33—C36—H36C	109.5

C16—C13—P1	105.4 (4)	H36A—C36—H36C	109.5
C13—C14—H14A	109.5	H36B—C36—H36C	109.5
C13—C14—H14B	109.5	C38—C37—C39	110.4 (5)
H14A—C14—H14B	109.5	C38—C37—C40	109.2 (5)
C13—C14—H14C	109.5	C39—C37—C40	107.9 (5)
H14A—C14—H14C	109.5	C38—C37—P2	109.5 (4)
H14B—C14—H14C	109.5	C39—C37—P2	105.2 (3)
C13—C15—H15A	109.5	C40—C37—P2	114.5 (4)
C13—C15—H15B	109.5	C37—C38—H38A	109.5
H15A—C15—H15B	109.5	C37—C38—H38B	109.5
C13—C15—H15C	109.5	H38A—C38—H38B	109.5
H15A—C15—H15C	109.5	C37—C38—H38C	109.5
H15B—C15—H15C	109.5	H38A—C38—H38C	109.5
C13—C16—H16A	109.5	H38B—C38—H38C	109.5
C13—C16—H16B	109.5	C37—C39—H39A	109.5
H16A—C16—H16B	109.5	C37—C39—H39B	109.5
C13—C16—H16C	109.5	H39A—C39—H39B	109.5
H16A—C16—H16C	109.5	C37—C39—H39C	109.5
H16B—C16—H16C	109.5	H39A—C39—H39C	109.5
C20—C17—C18	108.6 (4)	H39B—C39—H39C	109.5
C20—C17—C19	109.8 (5)	C37—C40—H40A	109.5
C18—C17—C19	107.5 (4)	C37—C40—H40B	109.5
C20—C17—P1	109.2 (3)	H40A—C40—H40B	109.5
C18—C17—P1	116.0 (4)	C37—C40—H40C	109.5
C19—C17—P1	105.6 (3)	H40A—C40—H40C	109.5
C17—C18—H18A	109.5	H40B—C40—H40C	109.5
C17—C18—H18B	109.5	F6—Sb1—F4	90.2 (5)
H18A—C18—H18B	109.5	F6—Sb1—F5	90.3 (6)
C17—C18—H18C	109.5	F4—Sb1—F5	93.2 (5)
H18A—C18—H18C	109.5	F6—Sb1—F3	177.2 (4)
H18B—C18—H18C	109.5	F4—Sb1—F3	92.5 (4)
C17—C19—H19A	109.5	F5—Sb1—F3	90.3 (5)
C17—C19—H19B	109.5	F6—Sb1—F2	91.5 (5)
H19A—C19—H19B	109.5	F4—Sb1—F2	89.4 (3)
C17—C19—H19C	109.5	F5—Sb1—F2	176.8 (4)
H19A—C19—H19C	109.5	F3—Sb1—F2	87.8 (3)
H19B—C19—H19C	109.5	F6—Sb1—F1	87.9 (4)

C17—C20—H20A	109.5	F4—Sb1—F1	176.4 (4)
C17—C20—H20B	109.5	F5—Sb1—F1	89.8 (4)
H20A—C20—H20B	109.5	F3—Sb1—F1	89.4 (3)
C17—C20—H20C	109.5	F2—Sb1—F1	87.6 (3)
H20A—C20—H20C	109.5	Cl1—C41—Cl2	111.1 (5)
H20B—C20—H20C	109.5	Cl1—C41—H41A	109.4
C26—C21—C22	117.5 (4)	Cl2—C41—H41A	109.4
C26—C21—P2	117.2 (4)	Cl1—C41—H41B	109.4
C22—C21—P2	124.9 (4)	Cl2—C41—H41B	109.4
C23—C22—C21	119.2 (4)	H41A—C41—H41B	108.0
C17—P1—C1—C6	71.1 (5)	C37—P2—C21—C26	71.9 (4)
C13—P1—C1—C6	-48.0 (5)	C33—P2—C21—C26	-46.7 (5)
Au1—P1—C1—C6	-171.5 (4)	Au1—P2—C21—C26	-171.4 (3)
C17—P1—C1—C2	-102.5 (5)	C37—P2—C21—C22	-100.9 (4)
C13—P1—C1—C2	138.4 (5)	C33—P2—C21—C22	140.5 (4)
Au1—P1—C1—C2	14.9 (6)	Au1—P2—C21—C22	15.8 (5)
C6—C1—C2—C3	-1.4 (10)	C26—C21—C22—C23	-1.8 (7)
P1—C1—C2—C3	172.1 (6)	P2—C21—C22—C23	170.9 (4)
C6—C1—C2—C7	174.5 (7)	C26—C21—C22—C27	175.7 (5)
P1—C1—C2—C7	-11.9 (9)	P2—C21—C22—C27	-11.5 (7)
C1—C2—C3—C4	2.3 (14)	C21—C22—C23—C24	1.6 (8)
C7—C2—C3—C4	-174.2 (9)	C27—C22—C23—C24	-176.2 (5)
C2—C3—C4—C5	-0.8 (17)	C22—C23—C24—C25	-0.2 (9)
C3—C4—C5—C6	-1.3 (16)	C23—C24—C25—C26	-1.0 (9)
C4—C5—C6—C1	2.1 (12)	C24—C25—C26—C21	0.8 (9)
C2—C1—C6—C5	-0.7 (10)	C22—C21—C26—C25	0.7 (8)
P1—C1—C6—C5	-174.8 (6)	P2—C21—C26—C25	-172.7 (4)
C1—C2—C7—C8	-67.8 (9)	C23—C22—C27—C28	-63.8 (6)
C3—C2—C7—C8	108.3 (8)	C21—C22—C27—C28	118.6 (6)

C1—C2—C7—C12	124.4 (7)	C23—C22—C27—C32	104.9 (6)
C3—C2—C7—C12	-59.5 (9)	C21—C22—C27—C32	-72.7 (7)
C12—C7—C8—C9	2.8 (10)	C32—C27—C28—C29	-0.5 (8)
C2—C7—C8—C9	-165.2 (6)	C22—C27—C28—C29	168.6 (5)
C7—C8—C9—C10	-1.0 (11)	C27—C28—C29—C30	-2.2 (8)
C8—C9—C10—C11	-1.7 (13)	C28—C29—C30—C31	3.4 (9)
C9—C10—C11—C12	2.6 (15)	C29—C30—C31—C32	-1.9 (10)
C8—C7—C12—C11	-1.9 (11)	C28—C27—C32—C31	1.9 (8)
C2—C7—C12—C11	166.2 (7)	C22—C27—C32—C31	-167.1 (6)
C10—C11—C12—C7	-0.8 (14)	C30—C31—C32—C27	-0.7 (10)
C1—P1—C13—C15	-158.9 (4)	C21—P2—C33—C35	-43.5 (5)
C17—P1—C13—C15	88.8 (4)	C37—P2—C33—C35	-155.3 (4)
Au1—P1—C13—C15	-27.4 (4)	Au1—P2—C33—C35	89.3 (4)
C1—P1—C13—C14	80.1 (5)	C21—P2—C33—C34	-160.1 (4)
C17—P1—C13—C14	-32.2 (5)	C37—P2—C33—C34	88.0 (5)
Au1—P1—C13—C14	-148.4 (4)	Au1—P2—C33—C34	-27.3 (5)
C1—P1—C13—C16	-42.8 (4)	C21—P2—C33—C36	80.0 (5)
C17—P1—C13—C16	-155.1 (4)	C37—P2—C33—C36	-31.9 (6)
Au1—P1—C13—C16	88.7 (4)	Au1—P2—C33—C36	-147.2 (4)
C1—P1—C17—C20	-169.7 (4)	C21—P2—C37—C38	-167.3 (4)
C13—P1—C17—C20	-55.6 (4)	C33—P2—C37—C38	-52.9 (5)
Au1—P1—C17—C20	62.2 (4)	Au1—P2—C37—C38	64.4 (4)
C1—P1—C17—C18	-46.6 (4)	C21—P2—C37—C39	74.1 (4)
C13—P1—C17—C18	67.5 (4)	C33—P2—C37—C39	-171.5 (4)
Au1—P1—C17—C18	-174.7 (3)	Au1—P2—C37—C39	-54.3 (4)
C1—P1—C17—C19	72.3 (4)	C21—P2—C37—C40	-44.2 (5)
C13—P1—C17—C19	-173.6 (4)	C33—P2—C37—C40	70.2 (5)
Au1—P1—C17—C19	-55.8 (4)	Au1—P2—C37—C40	-172.6 (4)

Least-squares planes (x,y,z in crystal coordinates) and deviations from them

(* indicates atom used to define plane)

- 1.0309 (0.0220) x + 9.4614 (0.0057) y + 7.5325 (0.0091) z = 6.7256 (0.0066)

*   0.0093 (0.0001) Au1

*   -0.0046 (0.0001) P1

*   -0.0046 (0.0000) P2

Rms deviation of fitted atoms =   0.0066

8.6435 (0.0382) x + 3.3304 (0.0216) y + 7.4589 (0.0411) z = 6.8419 (0.0120)

Angle to previous plane (with approximate esd) = 54.532 (0.169)

*   0.0618 (0.0036) P1

*   -0.0557 (0.0043) C1

*   -0.0442 (0.0060) C2

*   0.0271 (0.0072) C3

*   0.0390 (0.0087) C4

*   -0.0014 (0.0062) C5

*   -0.0267 (0.0055) C6

-0.2686 (0.0082) Au1

Rms deviation of fitted atoms =   0.0411

- 4.1335 (0.0435) x + 8.3880 (0.0253) y - 6.8151 (0.0455) z = 2.0352 (0.0338)

Angle to previous plane (with approximate esd) = 68.430 (0.239)

* 0.0160 (0.0047) C7

* -0.0127 (0.0049) C8

* -0.0029 (0.0057) C9

* 0.0153 (0.0066) C10

* -0.0118 (0.0068) C11

* -0.0040 (0.0057) C12

Rms deviation of fitted atoms = 0.0117

- 8.8497 (0.0118) x + 8.4717 (0.0073) y + 1.9770 (0.0338) z = 1.8370 (0.0193)

Angle to previous plane (with approximate esd) = 35.311 (0.263)

* -0.0774 (0.0025) P2

* 0.0656 (0.0039) C21

* 0.0505 (0.0038) C22

* -0.0248 (0.0039) C23

* -0.0508 (0.0044) C24

* -0.0064 (0.0041) C25

* 0.0432 (0.0043) C26

Rms deviation of fitted atoms = 0.0506

- 13.7578 (0.0086) x - 1.6641 (0.0358) y + 6.0125 (0.0196) z = 1.4317 (0.0067)

Angle to previous plane (with approximate esd) = 60.076 (0.174)

* 0.5991 (0.0045) C27

* 0.0397 (0.0043) C28

```
*   -0.4080 (0.0046)  C29

*   -0.2585 (0.0053)  C30

*    0.2629 (0.0042)  C31

*   -0.2351 (0.0018)  C33

Rms deviation of fitted atoms =   0.3460
```

Figure 1. shows a perspective drawing of crystalline $[Au(P(C_4H_9)_2(C_{12}H_9))_2](SbF_6)$ - CH_2Cl_2. P, F and Cl atoms are represented by dotted spheres, Au atoms are represented by cross-hatched spheres, Sb atoms are represented by shaded spheres and carbon and hydrogen atoms are represented by medium and small open spheres, respectively. All nonhydrogen atoms are labeled.

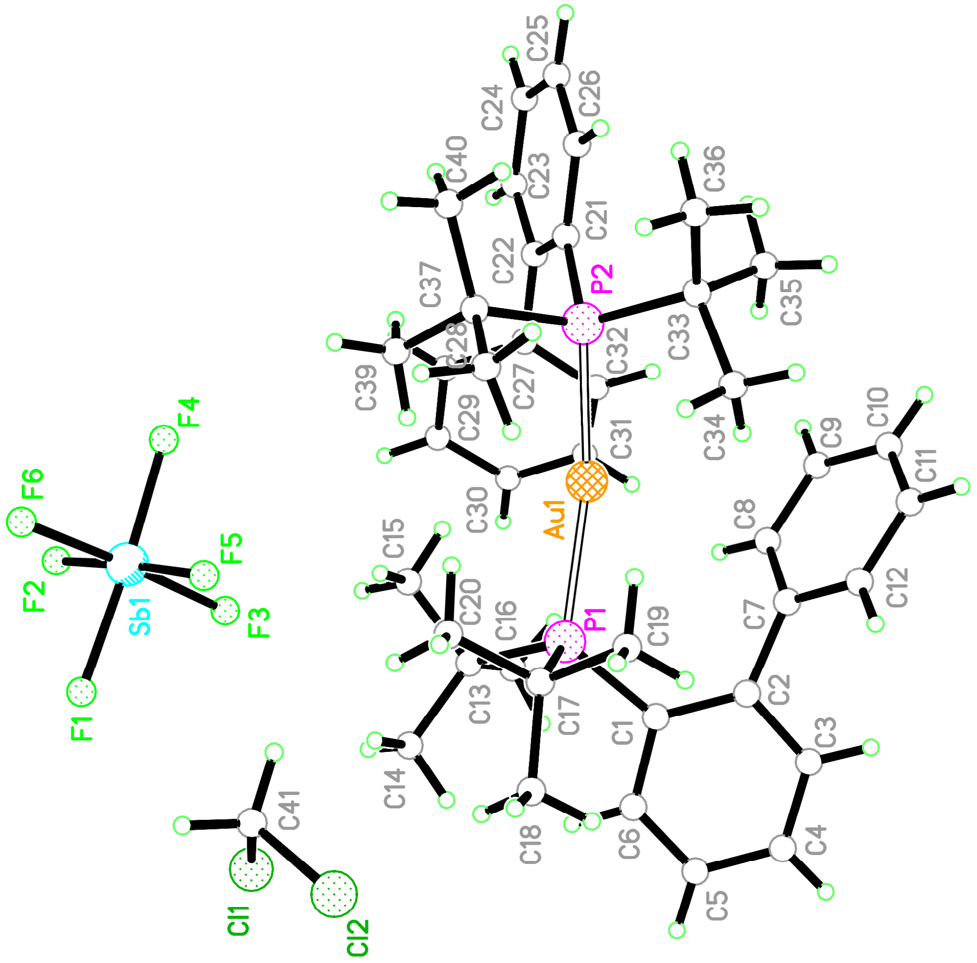

Figure 2. shows a perspective drawing of the cation in crystalline $[Au(P(C_4H_9)_2(C_{12}H_9))_2]$- (SbF_6) - CH_2Cl_2. Nonhydrogen atoms are represented by 50% probability ellipsoids. Hydrogen atoms are represented by arbitrarily-small spheres, which are in no way representative of their true thermal motion.

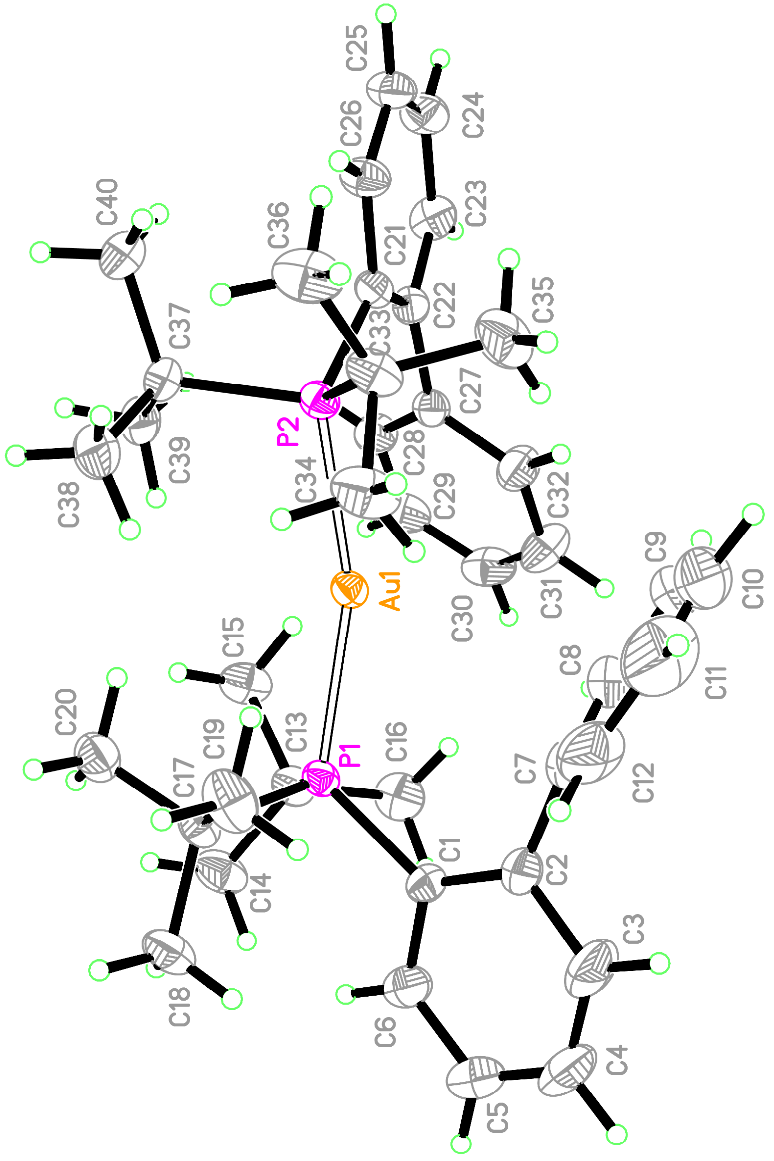

Figure 3. shows a perspective drawing of the cation in crystalline $[Au(P(C_4H_9)_2(C_{12}H_9))_2]$-$(SbF_6)$ - CH_2Cl_2 with all atoms represented as described in Figure 1.

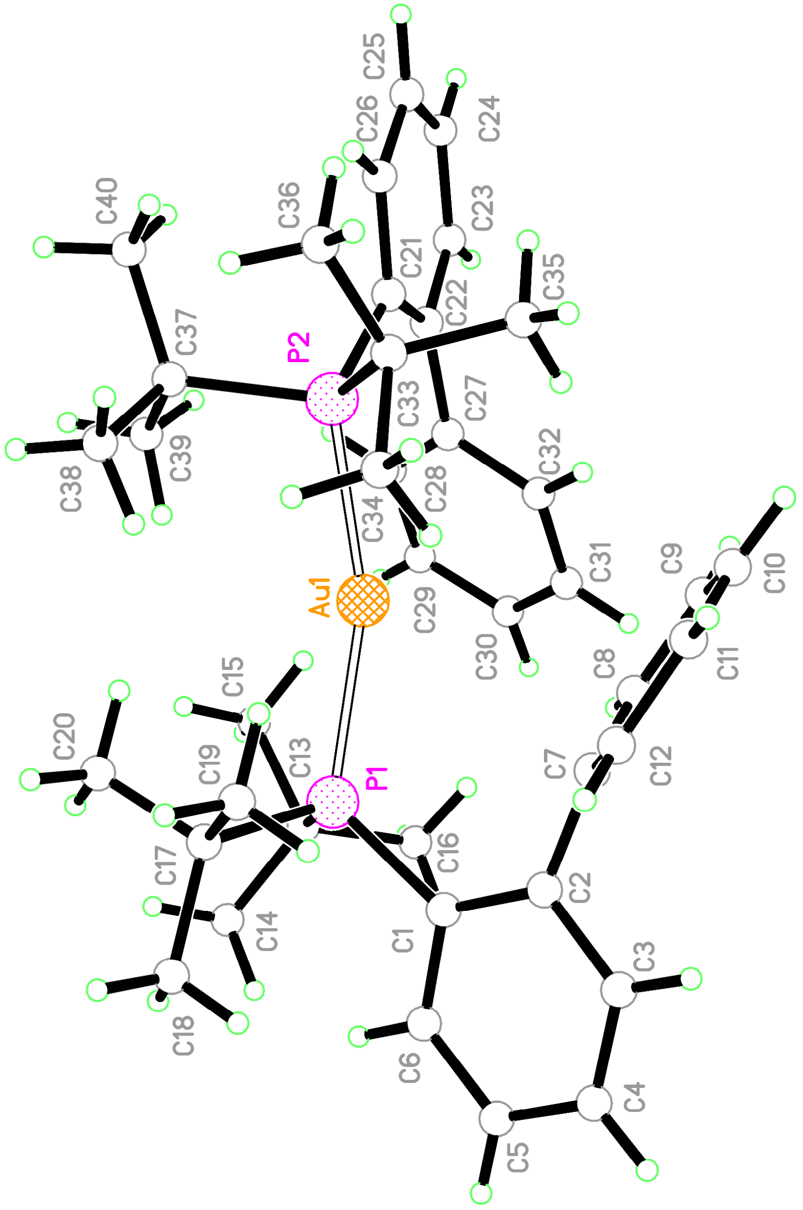

Figure 4. shows a perspective drawing of the cation in crystalline $[Au(P(C_4H_9)_2(C_{12}H_9))_2]$-$(SbF_6)$ - CH_2Cl_2 viewed down the P···P vector. Nonhydrogen atoms are represented by 50% probability ellipsoids. Hydrogen atoms have been omitted for clarity.

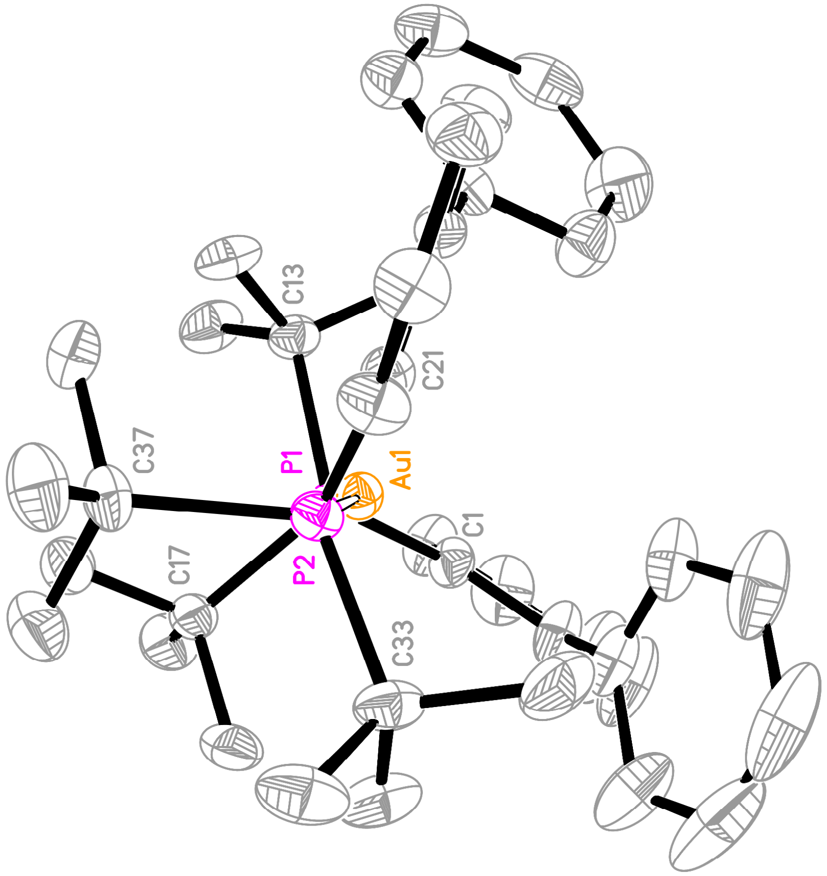

Figure 5. shows a perspective drawing of the anion and solvent molecule of crystallization present in the solid state structure of $[Au(P(C_4H_9)_2(C_{12}H_9))_2](SbF_6)$ - CH_2Cl_2. Nonhydrogen atoms are represented by 50% probability ellipsoids. Hydrogen atoms are represented by arbitrarily-small spheres.

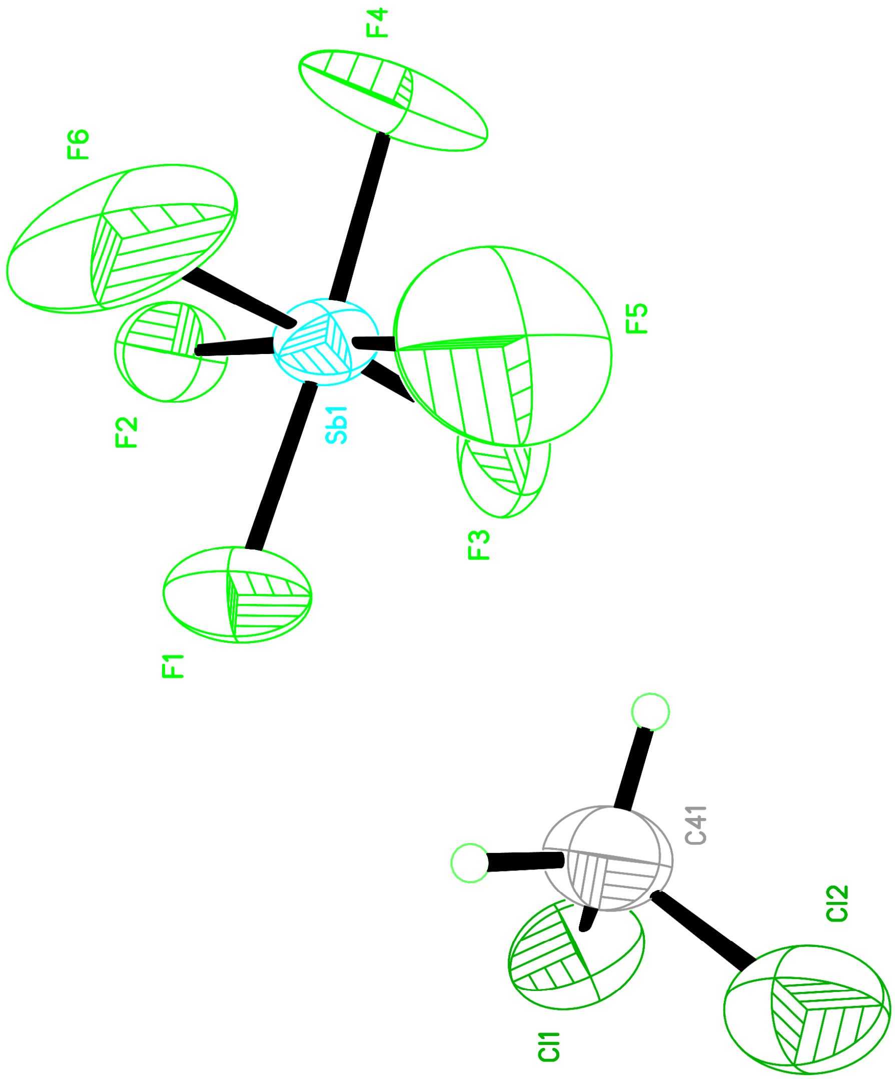

Figure 6. shows a projection of the unit cell when viewed down the $\vec{b}$ axis in crystalline $[Au(P(C_4H_9)_2(C_{12}H_9))_2](SbF_6) - CH_2Cl_2$ with atoms represented as in Figure 1. Hydrogen atoms have been omitted for purposes of clarity.

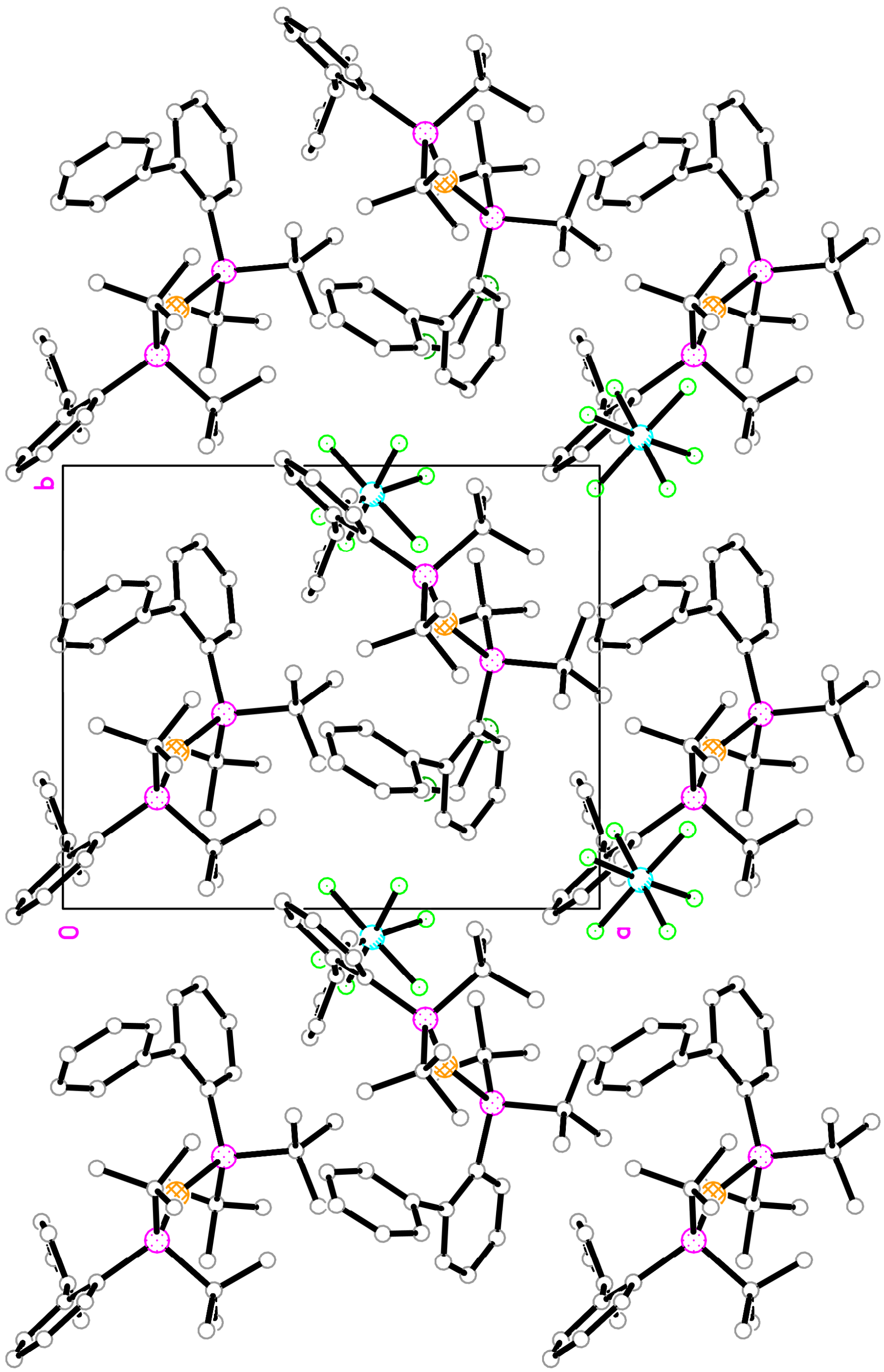

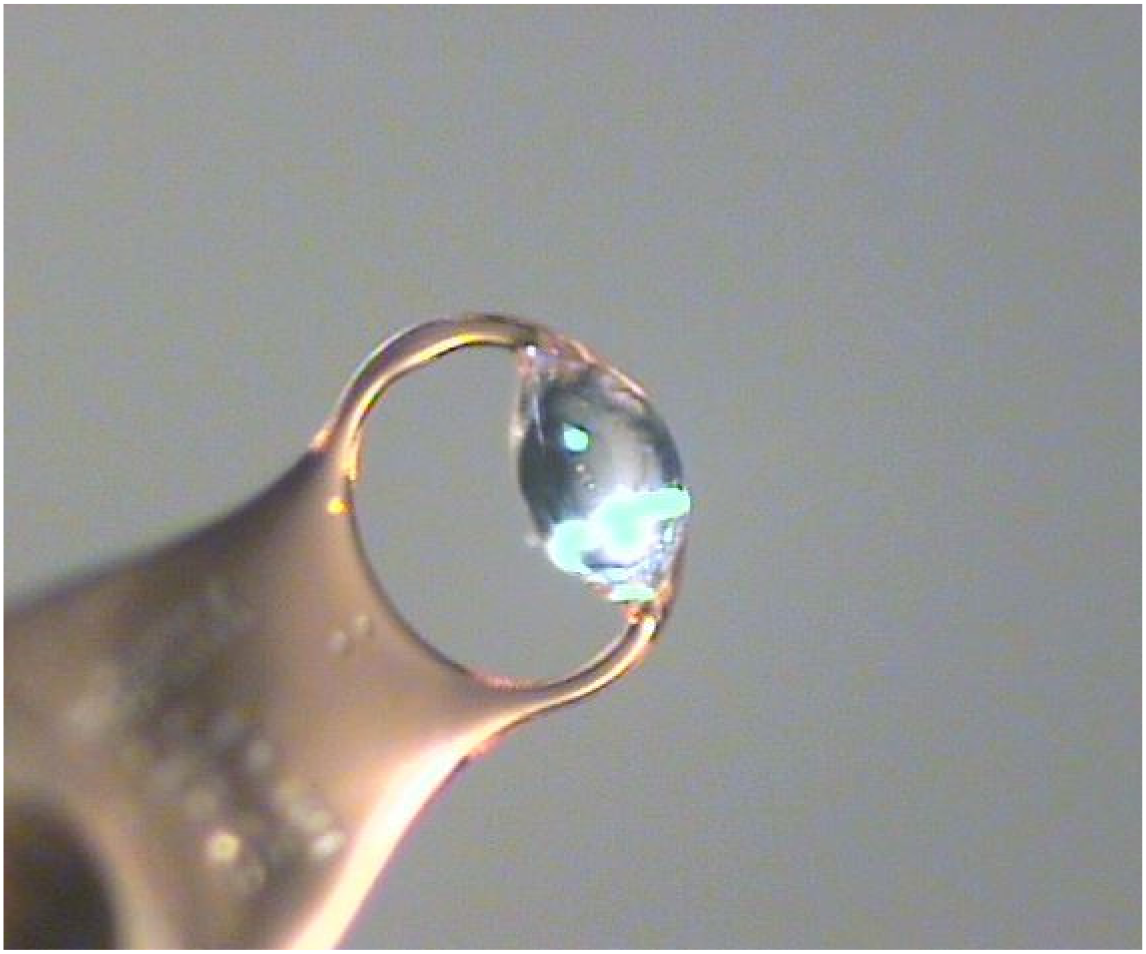

Data Crystal
